# Organocatalyst-mediated, pot-economical total synthesis of latanoprost†

**DOI:** 10.1039/d3sc02978f

**Published:** 2023-08-01

**Authors:** Genki Kawauchi, Yurina Suga, Shunsuke Toda, Yujiro Hayashi

**Affiliations:** a Department of Chemistry, Graduate School of Science, Tohoku University Sendai 980-8578 Japan yujiro.hayashi.b7@tohoku.ac.jp

## Abstract

The enantioselective total synthesis of latanoprost, an antiglaucoma agent, has been accomplished with excellent diastereo- and enantioselectivities in a pot-economical manner using six reaction vessels. An enantioselective Krische allylation was conducted in the first pot. In the second pot, olefin metathesis, silyl protection, and hydrogenolysis proceeded efficiently. In the third pot, an organocatalyst-mediated Michael reaction proceeded with excellent diastereoselectivity. The fourth pot involved a substrate-controlled Mukaiyama intramolecular aldol reaction and elimination of HNO_2_ to afford a methylenecyclopentanone, also with excellent diastereoselectivity. The fifth pot involved a Michael reaction of vinyl cuprate. In the sixth pot, three reactions, a *cis*-selective olefin metathesis, diastereoselective reduction, and deprotection, afforded latanoprost. Nearly optically pure latanoprost was obtained, and the total yield was 24%.

## Abstract 2

The six-pots synthesis of latanoprost has been accomplished with excellent diastereo- and enantioselectivities through an organocatalyst-mediated Michael reaction, a substrate-controlled intramolecular Mukaiyama aldol reaction as key steps.

## Graphical abstract

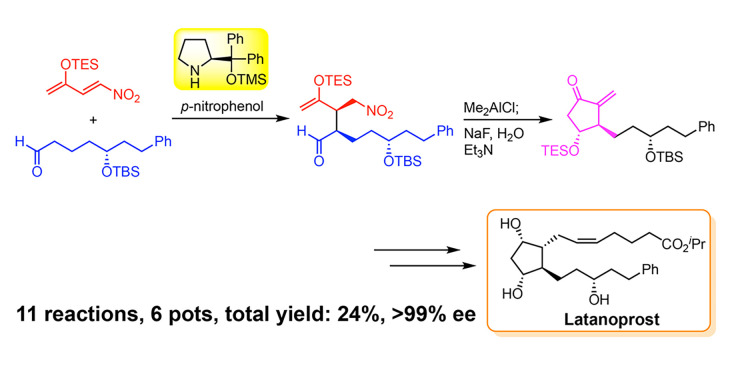

## Introduction

Prostaglandins are an important class of molecules with potent biological activities, and many related drugs have been developed.^1^ Many methods have been developed for the synthesis of prostaglandins,^2^ including Corey's famous syntheses *via* the Corey lactone^3^ and Noyori's three-component coupling process.^4^ Because of the importance of prostaglandins, the development of new synthetic methods for prostaglandins is still a significant topic in synthetic organic chemistry. Latanoprost (1) is an antiglaucoma agent and an analog of the prostaglandin PGF_2α_.^5^ As it is a blockbuster drug developed by Pharmacia, it is one of the important targets in the synthesis of prostaglandins.

Recently, the field of organocatalysis has developed rapidly^6^ and organocatalyst-mediated reactions have been successfully employed in the synthesis of prostaglandins. A proline-mediated aldol reaction of succinaldehyde was a key step in Aggarwal's synthesis.^7^ An organocatalytic Baeyer–Villiger oxidation was used by Peng and Chen,^8^ while Oger and Galano employed an organocatalyst-mediated intramolecular Michael reaction of a formyl-enal derivative.^9^

We propose the importance of “pot economy” because one-pot operations are efficient methods for making several bonds and can generate complex molecules in a single reaction vessel with several sequential reactions.^10^ Moreover, one-pot operations circumvent purification steps *via in situ* quenching, thereby minimizing chemical waste and saving time. Based on this concept, our group has investigated the synthesis of drugs and natural products in a small number of pots.^11^

Our group also has an interest in the organocatalyst-mediated synthesis of prostaglandins.^12^ In 2013, we reported the three-pot synthesis of prostaglandin E_1_ methyl ester.^12*a*^ Recently we reported a one-pot, 152-minute synthesis of the Corey lactone,^12*d*^ in which the key step was a formal asymmetric [3 + 2] cycloaddition reaction of ethyl 4-oxo-2-pentenoate and an α,β-unsaturated aldehyde catalyzed by diphenylprolinol silyl ether^13^ (eqn (1)). We synthesized latanoprost^12*e*^ and clinprost^12*f*^ based on this strategy.

## Results and discussion

**Scheme 1:**
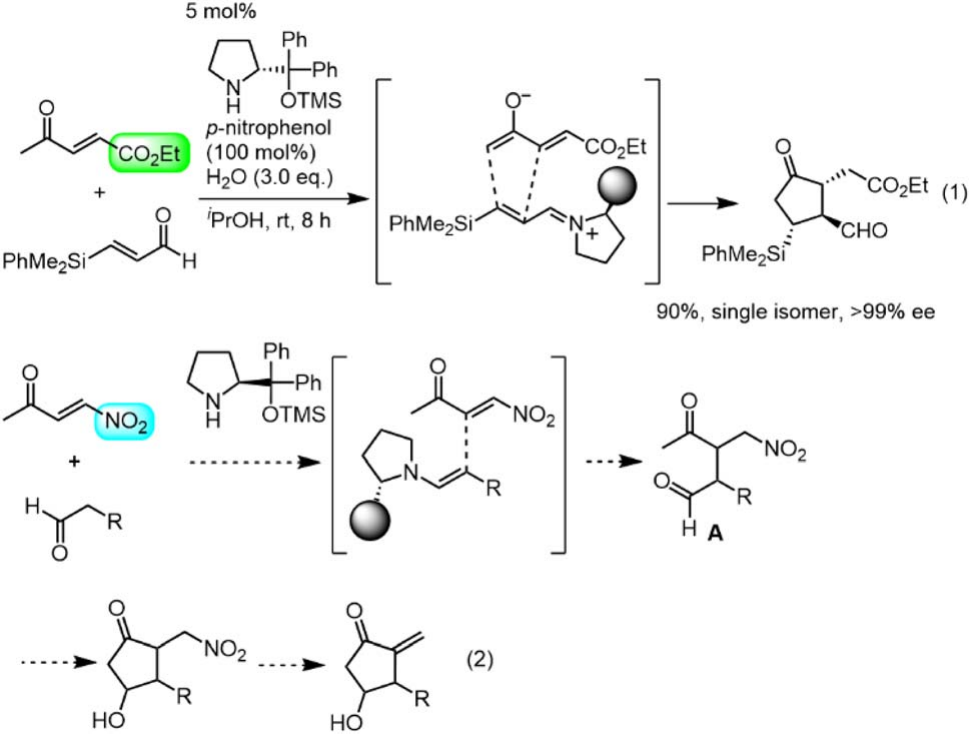
New idea for the synthesis of methylenecyclopentanone.

Our new idea for the synthesis of latanoprost was to use 4-nitro-3-butene-2-one instead of ethyl 4-oxo-2-pentenoate (eqn (2), Scheme 1): the ethyl ester was changed to a nitro group, which is not only a good electron-withdrawing group but also an excellent leaving group. The expected reactions were as follows: the Michael reaction of 4-nitrobut-3-ene-2-one and the aldehyde would proceed *via* an enamine intermediate to afford keto aldehyde A, according to our diphenylprolinol silyl ether-mediated Michael reaction of aldehydes and nitroalkenes.^13^ A substituted cyclopentanone would be synthesized by a subsequent intramolecular aldol reaction. As NO_2_ is a good leaving group, a methylenecyclopentanone, which is a key intermediate of prostaglandin F_2α_ reported by Stork and Isobe,^14^ would be formed by an E1cB reaction.

We examined the reaction of 4-nitrobut-3-en-2-one and 3-phenylpropanal as a model reaction (eqn (3)). Although the first Michael reaction proceeded, the second aldol reaction did not proceed under many different conditions. This was because the keto aldehyde A′ and the generated product underwent facile elimination of HNO_2_ and/or H_2_O. Next, we investigated the Mukaiyama aldol reaction^15^ in the second step. As it is difficult to prepare a silyl enol ether from A′ in the presence of an aldehyde, 4-nitro-2-siloxybuta-1,3-diene 6 was selected as the nitroalkene in the first step. Based on this reasoning, our retrosynthetic analysis is shown in Scheme 2.3
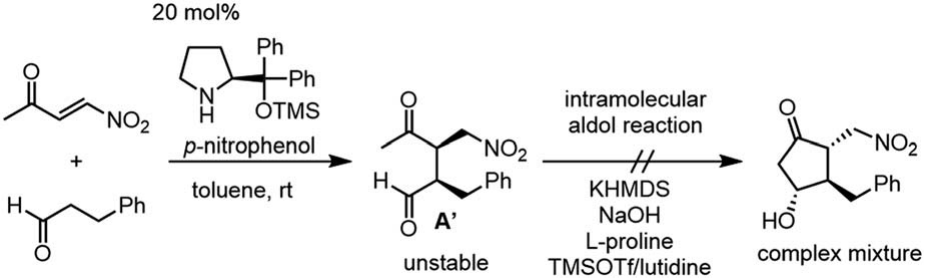


**Scheme 2:**
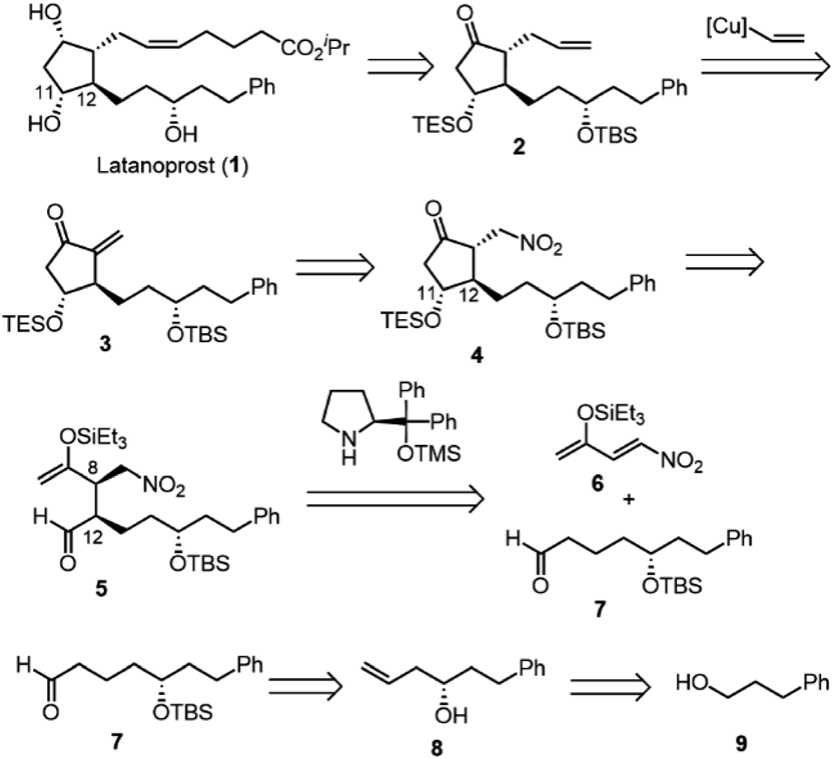
The retrosynthesis of latanoprost (1).

Latanoprost (1) would be synthesized from alkene 2*via* a *cis*-selective olefin metathesis, stereoselective reduction of the ketone, and deprotection. 2 would be prepared by a 1,4-addition of vinyl cuprate into methylenecyclopentanone 3. 3 would be synthesized by the elimination of the nitro group from 4, which would be prepared from 5 by an intramolecular Mukaiyama aldol reaction. An organocatalyst-mediated Michael reaction of 6 and 7 would afford 5. 7 would be prepared from 8, which would be synthesized by a Krische allylation from alcohol 9.

There are several concerns with this retrosynthesis. One is the reactivity of 6 as a Michael acceptor. Nitroalkene 6 has an electron-donating group, which would decrease its reactivity as a Michael acceptor. The other concern is the diastereoselectivity at C11 and C12. The C12 position has a chance to epimerize during the Mukaiyama aldol reaction. It was also a concern whether high diastereoselectivity at C11 would be obtained in the Mukaiyama aldol reaction.

**Scheme 3:**
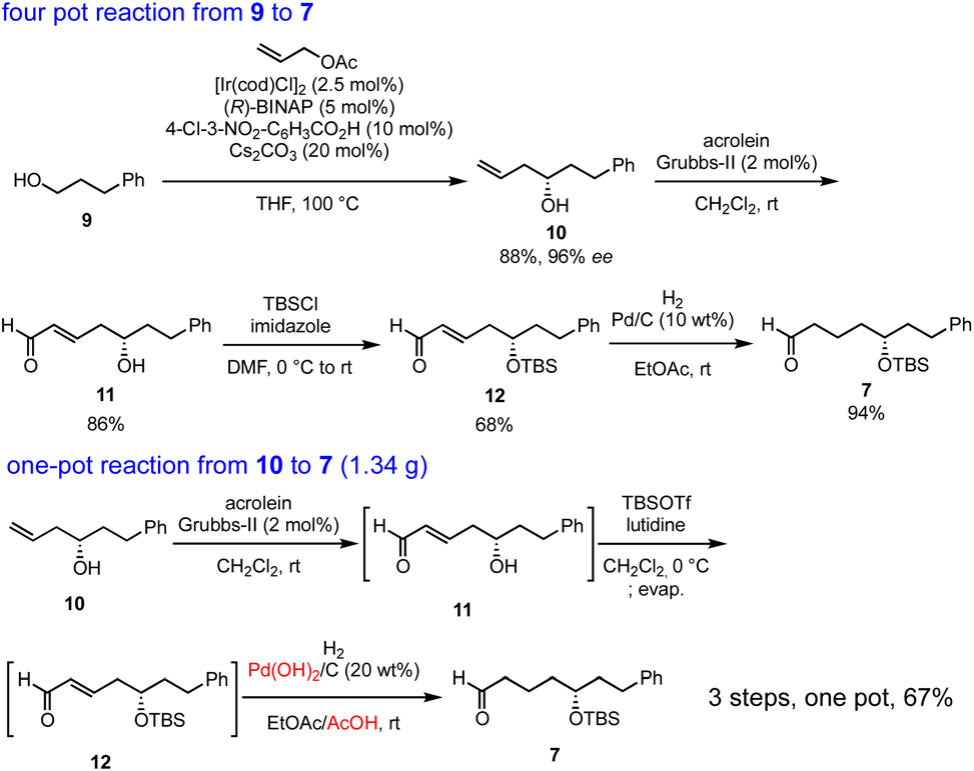
Four-pot reaction from 9 to 7 and one-pot reaction from 10 to 7.

Our synthesis commenced with Krische allylation^16^ of 3-phenylpropanol (9) to afford the allyl alcohol 10 in 88% yield with 96% ee (Scheme 3). Olefin metathesis of 10 with acrolein catalyzed by the Grubbs second generation catalyst^17^ proceeded to afford 11 in 86% yield. Alcohol protection with *tert*-butyldimethylsilyl chloride (TBSCl) provided 12. Hydrogenolysis using Pd/C gave aldehyde 7 in 94% yield.

The transformation from 10 into 7 could be conducted in a single vessel. After the olefin metathesis, the addition of TBSOTf and lutidine afforded 12. After evaporation and the addition of EtOAc, AcOH, and Pd(OH)_2_/C, hydrogenolysis proceeded under an H_2_ atmosphere to afford 7 in 67% yield over three steps in one pot. The use of Perlman's catalyst^18^ under acidic conditions (AcOH) is key to the success of the one-pot reaction. The reaction proceeded on a gram scale. Notably, the yield in the one-pot reaction (67%, 10 → 7) was higher than that of the stop-and-go method (55%, three steps).

Next was one of the key reactions. First, a nitroalkene with *tert*-butyldimethylsilyl enol ether 13 was used as a Michael acceptor. Despite our concern about the decrease in the reactivity of 13 as a Michael acceptor (*vide supra*), the reaction of nitroalkene 13 and aldehyde 7 proceeded efficiently using 20 mol% of the catalyst in the presence of *p*-nitrophenol (eqn (4)). The reaction was completed within 45 minutes at 0 °C to afford the Michael product 14 in 72% yield with good diastereoselectivity (dr = 88 : 12).4
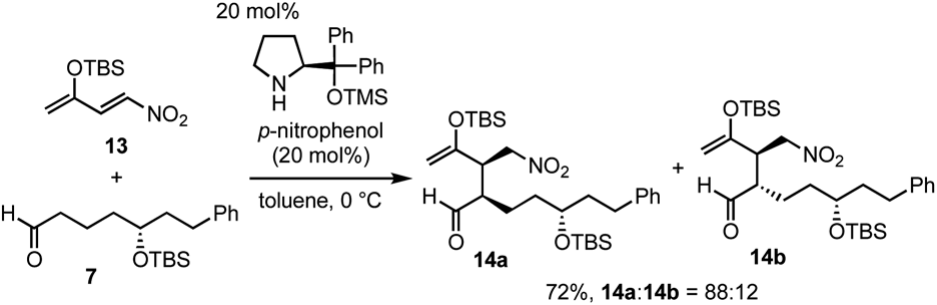


We then investigated the intramolecular aldol reaction. We found that the aldol product 15 was unstable. Thus, after the treatment of the Michael product 14 with a Lewis acid, the aldol product 15 was converted into methylene-cyclopentanone 16 using NaF and Et_3_N in the same reaction vessel. The yield and diastereoselectivity of 16 were determined (Table 1). Several Lewis acids are known to catalyze the Mukaiyama aldol reaction. The reaction did not proceed in the presence of Sc(OTf)_3_ ^19^ (entry 1). A combination of trimethylsilyl chloride (TMSCl) and SnCl_2_ ^20^ or trityl trifluoromethanesulfonate (TrOTf)^21^ gave a complex mixture (entries 2 and 3). A combination of TrCl and SnCl_2_ ^22^ afforded the product 16 in 20% yield, along with the deprotected alcohol 17 in 51% yield (entry 4). Me_2_AlCl^23^ afforded 16 in 46% yield with a good diastereoselectivity (dr = 6 : 1) and alcohol 17 in 10% (entry 5). To increase the yield of 16, we tried to suppress the deprotection of the TBS group, but there was no success.

**Table 1:** The effect of Lewis acid on the aldol reaction of 14^a^

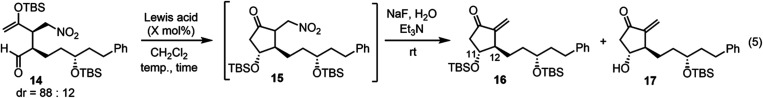						
Entry	Lewis acid	*X* [mol%]	Temp. [°C]	Time [h]	Yield^b^ [%]	dr^c^
1	Sc(OTf)_3_	20	−78 to 23	13	NR^d^	
2	TMSCl + SnCl_2_	20 + 20	−78 to 0	4	CM^e^	
3	TrOTf	20	−78	1	CM^e^	
4^f^	TrCl + SnCl_2_	20 + 20	−20	1	20	3 : 1
5^g^	Me_2_AlCl	70	−78	2	46	6 : 1

a Unless otherwise shown, reactions were performed by employing 14 (0.20 mmol) and a Lewis acid (0.040 mmol) in CH_2_Cl_2_ (4.0 mL) at the indicated temperature and time.

b Isolated yield of 16.

c The diastereomer ratio (C11 : C12) was determined by ^1^H-NMR analysis.

d NR = no reaction.

e CM = complex mixture.

f 17 was obtained in 51% yield.

g 17 was obtained in 10% yield.

**Fig. 1:**
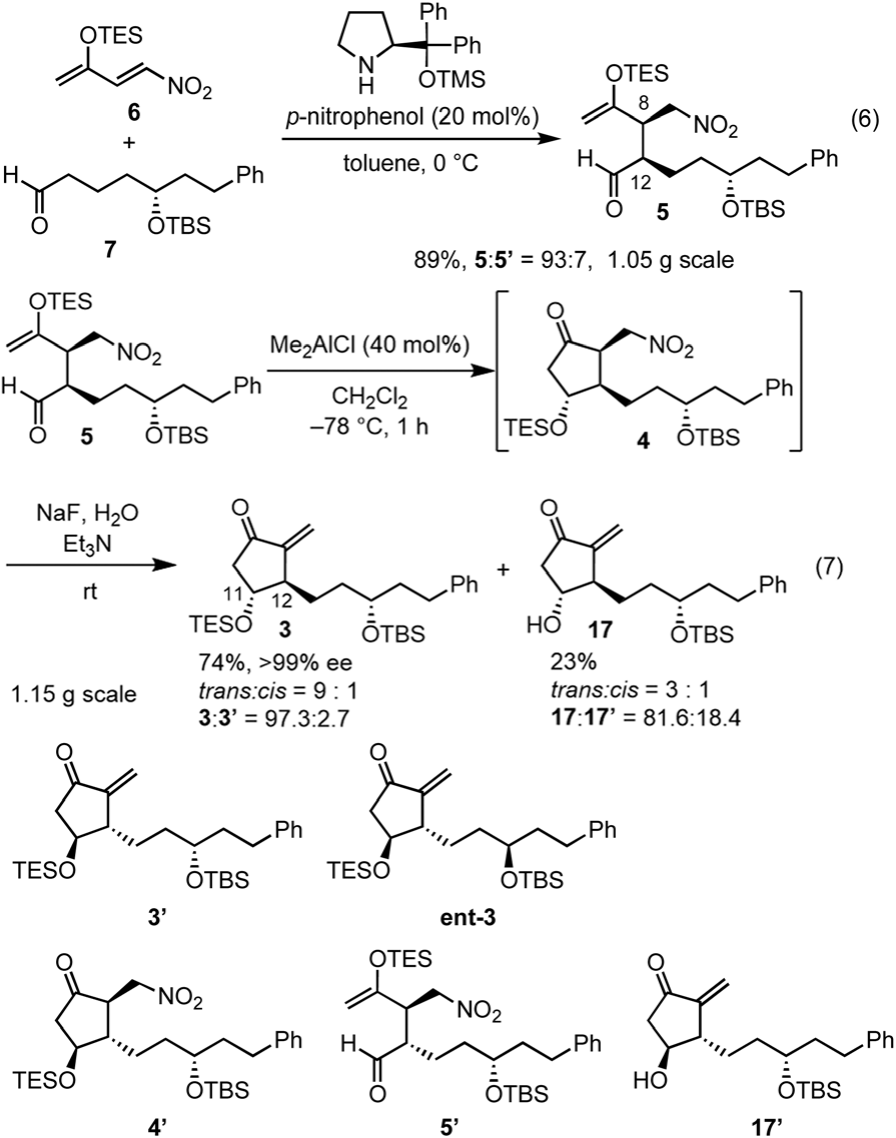
The other intermediates 3′, ent-3, **4′**, 5′ and 17′.

In the Mukaiyama aldol reaction, triethylsilyl enol ethers are more reactive than *tert*-butyldimethylsilyl enol ethers. Thus, we examined the reaction of the nitroalkene triethylsilyl enol ether 6. The first Michael reaction of 6 and 7 proceeded with a much higher yield (89%) and diastereoselectivity (5 : 5′ = 93 : 7, eqn (6), Fig. 1) than those of the reaction using the TBS enol ether 13 (eqn (4)). The aldol reaction and elimination of HNO_2_ proceeded efficiently using sequential treatment with Me_2_AlCl followed by NaF and Et_3_N to afford 3 in good yield (74%) along with alcohol 17 in 23% yield (eqn (7)). 3 possesses good diastereoselectivity: the *trans* : *cis* selectivity is 9 : 1, and the diastereomer ratio of 3 : 3′ is excellent (97.3 : 2.7). We also synthesized the enantiomer of 3 (ent-3), and prepared the racemic (±)-3 by mixing 3 and ent-3. The HPLC analysis of 3 and racemic (±)-3 using a chiral phase column indicated that the optical purity of 3 is over 99%.^24^

It was found that the diastereoselectivity of 17 : 17′ (81.6 : 18.4) is lower than that of 3 : 3′. As the epimerization from 5 to 5′ would proceed during the next Mukaiyama aldol reaction, the selectivity of 5 : 5′ would be much worse. Even though, the ratio of 3 : 3′ is higher than those of 5 : 5′ and 17 : 17′, which is synthetically useful. As 4 and 4′ are diastereomers, the reaction speed of the deprotection of the TES group would be different. It is very difficult to check the diastereoselectivity of 4 : **4′** because of the facile elimination of HNO_2_. The deprotection from **4′** would be faster than that from 4. Thus, kinetic resolution would occur to afford the higher diastereoselectivity in 3 with lower diastereoselectivity in 17 than in the parent 5.

It should be noted that TES enol ether 6 is superior to its TBS counterpart in terms of yield and selectivity in both the organocatalyst-mediated Michael reaction and the Mukaiyama aldol reaction. Both reactions proceeded efficiently on a gram scale. The stereochemistry at C12 was controlled by the diphenylprolinol silyl ether. At this stage, we could not definitively determine the stereochemistry at C11 by NMR analysis. However, we continued the total synthesis, hoping that 3 would possess the correct configuration (*vide infra*).

**Fig. 2:**
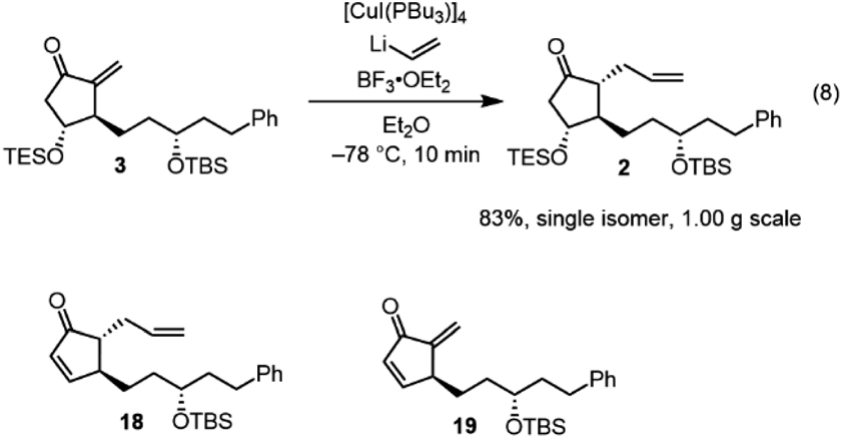
The side products in the Michael reaction of 3.

Next, we investigated the Michael addition of a vinyl anion to 3. Stork and Isobe reported the Michael reaction of a similar methylenecyclopentanone with a dialkenyl cuprate bearing a long alkyl chain.^14^ We found that the choices of the copper reagent and additive were important for the success of the reaction, as the siloxy elimination was a side reaction to afford products analogous to 18 and 19 (Fig. 2). For instance, when TMSCl was employed as an additive, 18 and 19 were generated at about 20% and 28%, respectively.^24^ The addition product 2 was obtained in good yield (83%) as a single isomer on a gram scale when [CuI(PBu_3_)]_4_ ^25^ and vinyl lithium in the presence of BF_3_·OEt_2_ ^26^ were employed (eqn (8)).

**Scheme 4:**
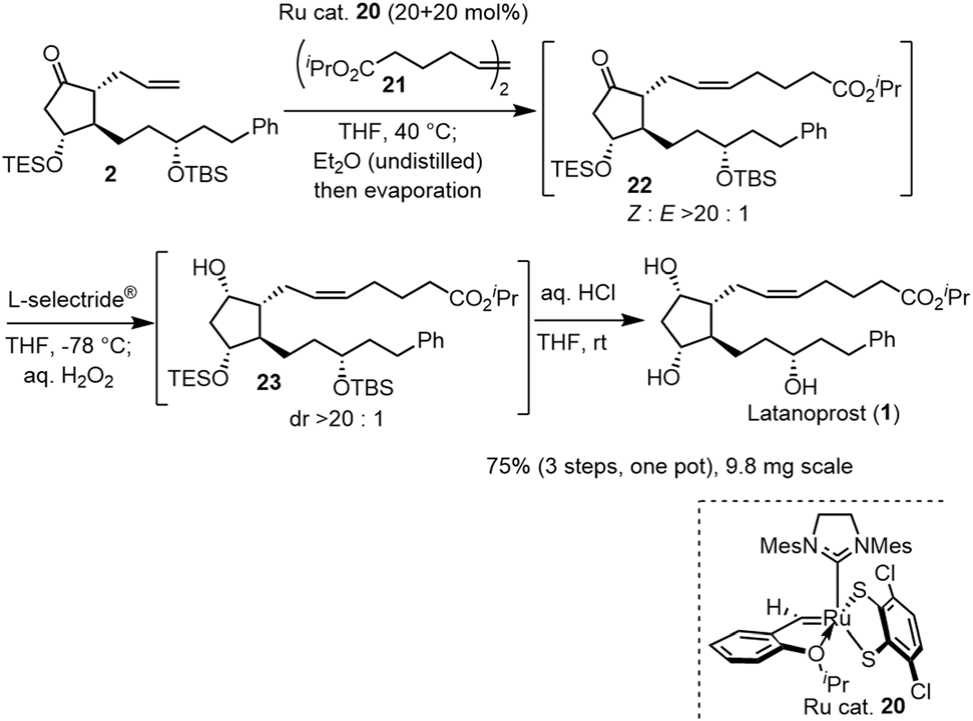
One-pot reaction from 2 to latanoprost (1).

The last three steps (2 → 1) were olefin metathesis, reduction, and deprotection, which could be conducted in a single vessel (Scheme 4). First, the *cis*-selective olefin metathesis^27^ proceeded when 2 was treated with alkene 21 ^27c^ in the presence of the Ru catalyst 20, which was developed by Grubbs and coworkers and used in the prostaglandin synthesis to afford 22 with excellent diastereoselectivity. The next reaction was the stereoselective reduction of the ketone employing L-selectride®. After olefin metathesis, the remaining Ru catalyst was deactivated by the addition of undistilled Et_2_O.^27*a*^ Then, after evaporation, the reduction proceeded efficiently with the addition of L-selectride® and THF in the same reaction vessel. The addition of aqueous H_2_O_2_ decomposed the remaining L-selectride®. With the further addition of aqueous HCl, deprotection of the two silyl groups afforded latanoprost (1). This was a one-pot reaction, and the yield of 1 from 2 was 75%. As latanoprost possesses very potent biological activity, the final step and purification must be carried out with great care. For the safety of the experimenters, the reaction was conducted on a 9.8 mg scale. The physical properties of the synthetic latanoprost (1) were identical in all respects to those in the reported data,^5*g*^ which confirmed the stereochemistry at C11 generated by the Mukaiyama aldol reaction (5 → 4).

## Conclusions

**Scheme 5:**
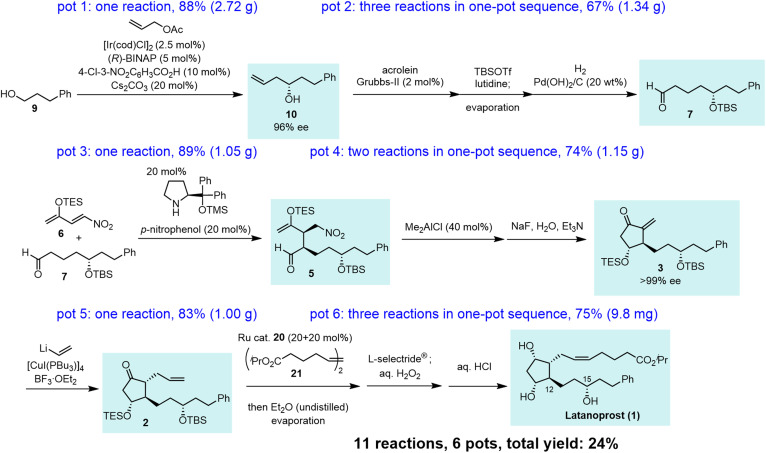
Six-pot total synthesis of latanoprost (1).

In summary, this is a highly diastereo- and enantioselective, six-pot total synthesis of latanoprost (1) with a total yield of 24% (Scheme 5). The first pot consists of a Krische allylation. The second pot involves three reactions: olefin metathesis, TBS protection, and hydrogenolysis. The third pot involves an organocatalyst-mediated Michael reaction of an aldehyde and nitroalkene. The fourth pot involves an intramolecular Michael reaction and an E1cB reaction with the elimination of HNO_2_. The fifth pot reaction is a Michael addition of vinyl cuprate. The sixth pot involves olefin metathesis, reduction, and deprotection. The present synthesis possesses several noteworthy features: (1) the stereochemistry at C15 was controlled by an asymmetric allylation developed by Krische and coworkers. (2) The stereochemistry at C12 was controlled by an organocatalytic reaction developed by our group. (3) A key substituted cyclopentanone was synthesized by an organocatalyst-mediated Michael reaction and a substrate-controlled intramolecular Mukaiyama aldol reaction, both of which proceeded with high diastereoselectivity. (4) The α-side chain was introduced to the methylenecyclopentanone *via* vinyl addition, followed by the *cis*-selective olefin metathesis. (5) This is the total synthesis of latanoprost with the fewest number of pots. (6) Nearly optically pure latanoprost was obtained.

## Data availability

General information, detailed experimental procedures, characterization data for compounds, and NMR, HPLC, IR spectra are available in the ESI.†

## Author contributions

G. K., Y. S., and S. T. performed the experiments. Y. H. conceived the concept and prepared the manuscript with feedback from G. K., Y. S., and S. T.

## Conflicts of interest

There are no conflicts to declare.

## Supplementary Material

SC-014-D3SC02978F-s001
