# Supplementary material for: Organocatalyst-mediated, pot-economical total synthesis of latanoprost
Source: Chem Sci. 2023 Aug 1;14(37):10081–6. doi: 10.1039/d3sc02978f (PMC10530343; doi:10.1039/d3sc02978f)
Supplement: SC-014-D3SC02978F-s001 [file SC-014-D3SC02978F-s001.pdf]

Electronic Supplementary Material (ESI) for Chemical Science.
This journal is © The Royal Society of Chemistry 2023

Materials and Methods

General Methods

General Remark: All vinylogous Michael reactions were carried out under atmosphere and monitored by NMR and thin-layer chromatography using Merck 60 F254 precoated silica gel plates (0.25 mm thickness). Specific optical rotations were measured using a JASCO P-1020 polarimeter and a JASCO DIP-370 polarimeter. FT-IR spectra were recorded on a JASCO FT/IR-410 spectrometer and a Perkin Elmer spectrum BX FT-IP spectrometer. ^{1}H and ^{13}C NMR spectra were recorded on an Agilent-400 MR (400 MHz for ^{1}H NMR, 100 MHz for ^{13}C NMR) instrument. Data for ^{1}H NMR are reported as chemical shift (δ ppm), integration multiplicity (s = singlet, d = doublet, t = triplet, q = quartet, dd = doublet of doublets, ddd = doublet of doublet of doublets, dt = double of triplets, m = multiplet), coupling constant (Hz), Data for ^{13}C NMR are reported as chemical shift. High resolution ESI-TOF mass spectra were measured by Themo Orbi-trap instrument. HPLC analysis was performed on a HITACHI Elite LaChrom Series HPLC, UV detection monitored at appropriate wavelength respectively, using CHIRALPACK® OD-H (0.46 cm × 25 cm) and CHIRALPACK® AS-H (0.46 cm × 25 cm). Melting point apparatus was Yanaco MP-J3. All the reagents were purchased from commercial sources (ALDRICH, Combi-Blocks, FUJIFILM Wako Chemicals, KANTO CHEMICAL, TCI), used without further purification.

All reactions were carried out in oven-dried (120 °C) or flame-dried glassware under an inert atmosphere of dry argon unless otherwise stated.

Solvents were purged with argon and purified under a positive pressure of dry argon by a modified Innovative Technologies purification system: toluene, THF, DMF, Et_2O, and CH_2Cl_2 were passed through activated alumina columns. Dehydrated EtOAc was used for the reaction without further purification (FUJIFILM Wako Chemicals, catalog No. 057-08175).

Preparation of nitroalkene

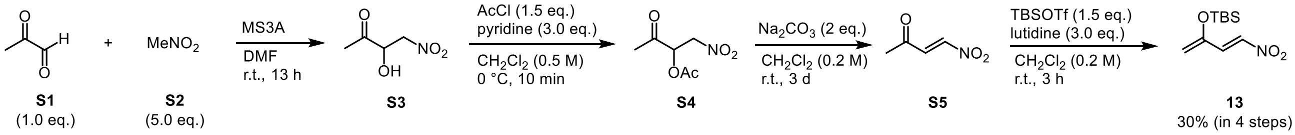

To a solution of methylglyoxal (**S1**) (40 wt% in H_2O, 4.6 mL, 30 mmol) in DMF (60 mL), nitromethane (**S2**) (8.0 mL, 0.15 mol, 5.0 eq.) and activated MS3Å (16 g) were added. After stirring at room temperature for 4 h, the reaction mixture was filtered through Celite®, washed with Et_2O, and concentrated in vacuo at 45 °C. Compound **S3** was obtained without column purification.

The crude was dissolved in anhydrous CH_2Cl_2 (121 mL) and added pyridine (5.3 mL, 66 mmol, 3.0 eq.) and acetyl chloride (2.4 mL, 33 mmol, 1.5 eq.) at 0 °C. After stirring at 0 °C for 10 min, the reaction mixture was quenched with 1N HCl (50 mL), extracted with CH_2Cl_2 (3×50 mL). The combined organic layer was washed with 1N HCl, water, and brine, dried with Na_2SO_4, filtered and concentrated in vacuo. Compound **S4** was obtained without column purification.

The crude was dissolved to unhydrous CH_2Cl_2 (108 mL) and added mashed Na_2CO_3 (4.6 g, 43 mol, 2 eq.) at 0 °C. After stirring at room temperature for 3 days, the reaction mixture was filtered through silica pad on Celite®, washed with CH_2Cl_2, and concentrated in vacuo. Compound **S5** was obtained without column purification.

The crude was dissolved to anhydrous CH_2Cl_2 (71 mL) and added 2,6-lutidine (4.9 mL, 42 mmol, 3.0 eq.) and TBSOTf (4.6 mL, 21 mmol, 1.5 eq.) at 0 °C. After stirring at room temperature for 3 h, the reaction mixture was quenched with sat. $NaHCO_3$ aq. (50 mL) and extracted with CH_2Cl_2 (3×50 mL). The combined organic layer was washed with water and brine, dried with Na_2SO_4, filtered and concentrated in vacuo. Purification by silica gel chromatography (*n*-Hexane: EtOAc = 15:1) gave compound **13** in 30% yield (4 steps, 2.1 g).

(*E*)-4-nitrobut-3-en-2-one

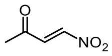

Physical State: yellow liquid

^{1}H NMR (400 MHz, $CDCl_3$) δ 7.53 (d, J = 14.0 Hz, 1H), 7.25 (d, J = 14.0 Hz, 1 H), 2.45 (s, 3H)

^{13}C NMR (100 MHz, $CDCl_3$) δ 194.9, 147.3, 132.2, 29.5

IR (neat) ν 2254, 1711, 1697, 1632, 1542, 1350, 1285, 1240, 912, 741, 651

R_f (*n*-Hexane: EtOAc = 1:1, color reagent: $KMnO_4$ reagent): 0.60

(*E*)-*tert*-butyldimethyl((4-nitrobuta-1,3-dien-2-yl)oxy)silane

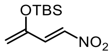

Physical State: orange liquid

^{1}H NMR (400 MHz, $CDCl_3$) δ 7.37 (d, J = 12.8 Hz, 1H), 7.22 (d, J = 12.8 Hz, 1H), 4.88 (d, J =1.6 Hz, 1H), 4.83 (d, J =1.6 Hz, 1H), 0.98 (s, 9H), 0.22 (s, 6H)

^{13}C NMR (100 MHz, $CDCl_3$) δ 232.0, 137.1, 133.9, 106.9, 25.6 (3C), 18.2, -4.7 (2C)

IR (neat) ν 2957, 2932, 2887, 2860, 1633, 1594, 1520, 1472, 1345, 1263, 1211, 1026, 954, 842, 785, 742
HRMS (ESI): $[M+Na]^+$ calcd for $C_{10}H_{19}O_3SiNa^+$: 252.1026, found: 252.1027
$\mathbf{R_f}$ (*n*-Hexane: EtOAc = 3:1, color reagent: *p*-anisaldehyde): 0.70

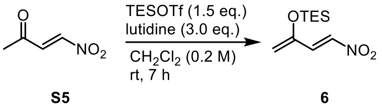

S5 (1.05 g, 9.12 mmol) was dissolved to anhydrous CH_2Cl_2 (46 mL) and added 2,6-lutidine (4.23 mL, 36.5 mmol, 3.0 eq.) and TESOTf (4.12 mL, 18.2 mmol, 2 eq.) at 0 ℃. After stirring at room temperature for 7 h, the reaction mixture was quenched with sat. aq. $NaHCO_3$ (40 mL) and extracted with CH_2Cl_2 (3 × 50 mL). The combined organic layer was washed with water and brine, dried with Na_2SO_4, filtered and added toluene (4 mL) and concentrated in vacuo (200 mm Hg, 25 °C). Purification by silica gel chromatography (*n*-Hexane/ EtOAc = 15/1) then combined organic layers were added toluene (4 mL) and concentration in vacuo (100 mmHg, 25 °C) gave compound **6** as a toluene solution.
(Compound **6** was unstable in concentration.)

(*E*)-triethyl((4-nitrobuta-1,3-dien-2-yl)oxy)silane

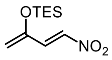

toluene solution
^{1}H NMR (400 MHz, $CDCl_3$) δ 7.36 (d, *J* = 12.8 Hz, 1H), 7.26-7.24 (m, 1H), 4.84 (dd, *J* = 11.2, 1.6 Hz, 2H), 1.02-0.97 (m, 9H), 0.78-0.74 (m, 6H)
$\mathbf{R_f}$ (*n*-hexane/ EtOAc = 3:1, color reagent: *p*-anisaldehyde): 0.70

Preparation of aldehyde

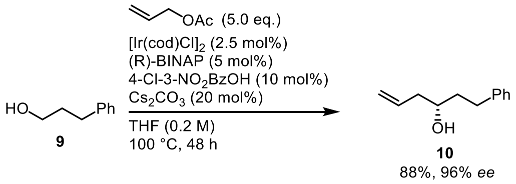

A pressure tube was charged with 3-phenylpropanol **9** (2.7 mL, 20 mmol), $[Ir(cod)Cl]_2$ (0.34 g, 0.50 mmol, 2.5 mol%), (*R*)-BINAP (0.62 g, 1.0 mmol, 5.0 mol%), 4-chloro-3-nitrobenzoic acid (0.40 g, 2.0 mmol, 10 mol%), cesium carbonate (1.3 g, 4.0 mmol, 20 mol%), anhydrous THF (40 mL), and allyl acetate (11 mL, 100 mmol, 5.0 eq.). The pressure tube was purged with argon, sealed and heated 100 °C (oil bath temperature) and stirred for 48 h. The reaction mixture was allowed to cool to room temperature and filtered through Celite® pad with the aid of CH_2Cl_2. The crude reaction mixture was concentrated in vacuo. Purification by silica gel chromatography (*n*-Hexane: EtOAc = 6 : 1) gave compound **10** in 88% yield (3.1 g).

(*S*)-1-phenylhex-5-en-3-ol

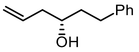

Physical State: pale yellow solid

^{1}H NMR (400 MHz, CDCl$_3$) δ 7.31-7.27 (m, 2H), 7.22-7.19 (m, 3H), 5.87-5.77 (m, 1H), 5.16-5.16 (m, 2H), 5.13-5.13 (m, 1H), 3.71-3.65 (m, 1H), 2.85-2.78 (m, 1H), 2.73-2.65 (m, 1H), 2.36-2.30 (m, 1H), 2.22-2.15 (m, 1H), 1.82-1.77 (m, 2H)

^{13}C NMR (100 MHz, CDCl$_3$) δ 141.9, 134.5, 128.3 (4C), 125.7, 118.0, 69.9, 41.9, 38.3, 31.9.

IR (neat) ν 3564, 3352, 3063, 3027, 3003, 2977, 2930, 2861, 1712, 1641, 1603, 1496, 1454, 1266, 1154, 1126, 1048, 995, 916, 865, 747, 700, 646

HRMS (ESI): $[M+Na]^+$ calcd for $C_{12}H_{16}ONa^+$: 199.1093, found: 199.1099

$[\alpha]_D^{22}$ -23.5 (*c* 0.68, $CHCl_3$)

mp. 33-34 ℃

R_f (*n*-Hexane: EtOAc = 3:1, color reagent: *p*-anisaldehyde): 0.48

The enantiomeric ratio was determined by HPLC using CHIRALPACK® OD-H (*n*-Hexane: *i*-PrOH = 50:1; flow rate 1.0 mL/min, major isomer t_R = 12 min, minor isomer t_R = 18 min) (96% *ee*)

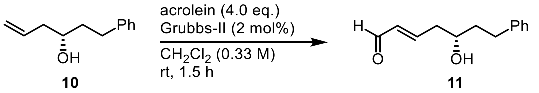

10 **11** 86%

A solution of compound **10** (0.56 g, 3.2 mmol) in anhydrous CH_2Cl_2 (9.6 mL) was freeze-pump-thaw for three times. Acrolein (0.85 mL, 13 mmol, 4.0 eq.) and Grubbs-Ⅱ (54 mg, 0.064 mmol, 2.0 mol%) were added at 0 °C then warmed to room temperature. After stirring at room temperature for 1.5 h, the reaction mixture was concentrated in vacuo. Purification by silica gel chromatography (*n*-Hexane: EtOAc = 4:1 to 2:1) gave compound **11** in 86% yield (0.56 g).

(*S*,*E*)-5-hydroxy-7-phenylhept-2-enal

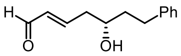

Physical State: colorless liquid

^{1}H NMR (400 MHz, CDCl$_3$) δ 9.53 (d, *J* = 8.0 Hz, 1H), 7.32-7.28 (m, 2H), 7.23-7.19 (m, 3H), 6.89 (dt, *J* = 15.2, 7.2 Hz, 1H), 6.18 (dd, *J* = 15.6 Hz, 1H), 3.88-3,82 (m, 1H), 2.82 (ddd, *J* = 14.4, 7.6, 7.6 Hz, 1H), 2.75-2.68 (m, 1H), 2.60-2.46 (m, 2H), 1.87-1.82 (m, 2H)

^{13}C NMR (100 MHz, CDCl$_3$) δ 193.9, 154.8, 141.3, 134.8, 128.4 (2C), 128.3 (2C), 126.0, 69.6, 40.6, 38.8, 31.8.

IR (neat) ν 3447, 3056, 3029, 2986, 2936, 2861, 2744, 1685, 1638, 1603, 1496, 1454, 1421, 1266, 1134, 1049, 1011, 976, 929, 897, 698

HRMS (ESI): $[M+Na]^+$ calcd for $C_{13}H_{16}O_2Na^+$: 227.1043, found: 227.1051

$[\alpha]_D^{25}$ -8.3 (*c* 1.06, $CHCl_3$)

R_f (*n*-Hexane: EtOAc = 2:1, color reagent: *p*-anisaldehyde): 0.29

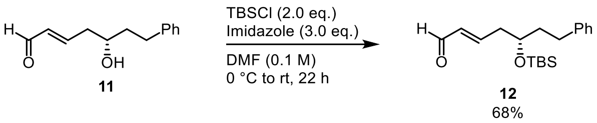

To a solution of compound **11** (0.54 g, 2.6 mmol) in anhydrous DMF (13 mL), imidazole (0.54 g, 7.9 mmol, 3.0 eq.) and TBSCl (0.80 g, 5.3 mmol, 2.0 eq.) were added at 0 °C. After stirring room temperature for 22 h, the reaction mixture was quenched with sat. NH_4Cl aq. at 0 °C and extracted with *n*-hexane/ EtOAc (3/1, 3×50 mL). The combined organic layer was washed with water and brine, dried with anhydrous Na_2SO_4, filtered and concentrated in vacuo. Purification by silica gel chromatography (*n*-Hhexane: EtOAc = 20:1 to 15:1) gave compound **12** in 68% yield (0.57 g).

(*S*,*E*)-5-((*tert*-butyldimethylsilyl)oxy)-7-phenylhept-2-enal

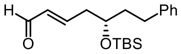

Physical State: yellow liquid

^{1}H NMR (400 MHz, $CDCl_3$) δ 9.52 (d, *J* = 8.0 Hz, 1H), 7.31-7.29 (m, 2H), 7.21-7.16 (m, 3H), 6.89 (dt, *J* = 15.6, 7.6 Hz, 1H), 6.14 (ddt, *J* = 15.6, 7.6, 1.2 Hz, 1H), 3.94-3.89 (m, 1H), 2.74-2.45 (m, 4H), 1.82-1.76 (m, 2H), 0.91 (s, 9 H), 0.08 (s, 3H), 0.06 (s, 3H)

^{13}C NMR (100 MHz, $CDCl_3$) δ 193.8, 154.9, 141.8, 134.9, 128.5 (2C), 128.3 (2C), 125.9, 70.6, 40.4, 39.1, 31.7, 25.8 (3C), 18.1, -4.4, -4.5.

IR (neat) ν 3028, 2952, 2929, 2857, 1694, 1638, 1496, 1472, 1462, 1362, 1256, 1137, 1072, 1031, 1007, 978, 938, 837, 776, 747, 700

HRMS (ESI): $[M+Na]^+$ calcd for $C_{19}H_{30}O_2SiNa^+$:341.1907, found: 341.1925

$[\alpha]_D^{25}$ -4.2 (*c* 0.82, $CHCl_3$)

R_f (*n*-Hexane: EtOAc = 5:1, color reagent: *p*-anisaldehyde): 0.58

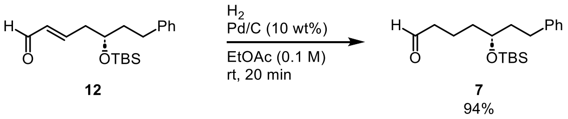

To a solution of compound **12** (0.56 g, 1.8 mmol) in EtOAc (18 mL), Pd/C (10 wt%, 0.056 g) was added at room temperature. The reaction mixture was purged with H_2. After stirring for 20 min under H_2 (balloon pressure), the reaction mixture was purged with N_2 and filtered through Celite®, washed with EtOAc, and concentrated in vacuo. Purification by silica gel chromatography (*n*-Hexane: EtOAc = 20:1 to 10:1) gave compound **7** in 94% yield (0.53 g).

(*R*)-5-((*tert*-butyldimethylsilyl)oxy)-7-phenylheptanal

Physical State: colorless liquid

^{1}H NMR (400 MHz, CDCl$_3$) δ 9.76 (t, *J* = 1.6 Hz, 1H), 7.30-7.27 (m, 2H), 7.19-7.16 (m, 3H), 3.74 (tt, *J* = 4.2, 4.2 Hz, 1H), 2.71-2.58 (m, 2H), 2.44 (td, *J* = 7.2, 1.6 Hz, 2H), 1.79-1.65 (m, 4H), 1.53-1.49 (m, 2H), 0.91 (s, 9H), 0.06 (s, 3H), 0.05 (s, 3H)

^{13}C NMR (100 MHz, CDCl$_3$) δ 202.5, 142.5, 128.3 (2C), 128.3 (2C), 125.7, 71.4, 43.9, 38.8, 36.2, 31.6, 25.9 (3C), 18.1, 17.8, -4.4 (2C)

IR (neat) ν 2952, 2929, 2857, 1727, 1472, 1461, 1255, 1097, 1065, 1006, 836, 774, 748, 699

HRMS (ESI): $[M+Na]^+$ calcd for $C_{19}H_{32}O_2SiNa^+$: 343.2064, found: 343.2017

$[\alpha]_D^{25}$ +2.1 (*c* 0.96, $CHCl_3$)

R$_f$ (*n*-Hexane: EtOAc = 7:1, color reagent: *p*-anisaldehyde): 0.38

One-pot operation

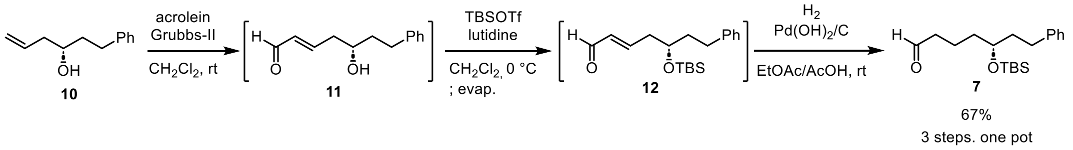

A solution of compound **10** (1.34 g, 7.60 mmol) in anhydrous CH_2Cl_2 (22.8 mL) was freeze-pump-thaw for three times. Acrolein (2.03 mL, 30.4 mmol, 4.0 eq.) and Grubbs-Ⅱ (129 mg, 0.152 mmol, 2.0 mol%) were added at 0 °C then warmed to room temperature. After stirring at room temperature for 4.5 h, reaction mixture was cooled to 0 °C. 2,6-lutidine (2.64 mL, 22.8 mmol, 3.0 eq.) and TBSOTf (3.49 mL, 15.2 mmol, 2.0 eq.) were added at 0 °C. After stirring at this temperature for 15 min, the reaction mixture was concentrated in vacuo. The resulting mixture was added EtOAc (38.0 mL), AcOH (38.0 mL) and $Pd(OH)_2/C$ (10 wt%, wet, 485 mg) and reaction vessel was purged with H_2. After stirring for 30 min at room temperature under H_2 (balloon pressure), the reaction mixture was purged with N_2 and filtered through Celite® and washed with EtOAc. The organic layer was washed with water and brine, dried with anhydrous Na_2SO_4, filtered and concentrated in vacuo. Purification by silica gel chromatography (*n*-Hexane: EtOAc = 30:1) gave compound **7** in 67% yield (1.63 g).

Asymmetric Michael reaction

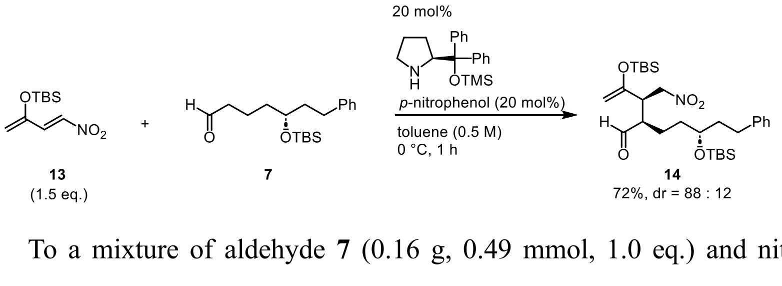

To a mixture of aldehyde **7** (0.16 g, 0.49 mmol, 1.0 eq.) and nitroalkene **13** (0.17 g, 0.74 mmol, 1.5 eq.) in anhydrous toluene (0.98 mL), *p*-nitrophenol (14 mg, 0.098 mmol, 20 mol%) and catalyst (32 mg, 0.098 mmol, 20 mol%) were added at 0 ℃. After stirring for 1 h at 0 ℃, the reaction mixture was purified by silica gel chromatography (*n*-Hexane/ EtOAc = 15/1). Compound **14** was obtained in 72% yield (0.19 g) as a diastereomixture.

(2*R*,5*R*)-5-((*tert*-butyldimethylsilyl)oxy)-2-((*R*)-3-((*tert*-butyldimethylsilyl)oxy)-1-nitrobut-3-en-2-yl)-7-phenylheptanal

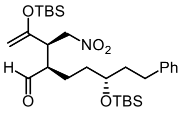

dr = 88 : 12

Physical State: yellow liquid

^{1}H NMR (400 MHz, CDCl$_3$) δ 9.65 (d, *J* = 2.8 Hz, 1 H), 7.30-7.28 (m, 2H), 7.20-7.14 (m, 3H), 4.52-4.41 (m, 2H), 3.72-3.66 (m, 1H), 3.22-3.16 (m, 1H), 2.61-2.56 (m, 2H), 2.53-2.47 (m, 1H), 1.80-1.67 (m, 4H), 1.59-1.50 (m, 2H), 1.43-1.35 (m, 1H), 0.93 (s, 9H), 0.90 (s, 9H), 0.19 (s, 6H), 0.05 (s, 3H), 0.02 (s, 3H)

^{13}C NMR (100 MHz, CDCl$_3$) δ 202.9, 152.9, 142.1, 128.4 (2C), 128.2 (2C), 125.8, 93.5, 75.7, 71.4, 50.7, 44.7, 38.5, 33.3, 31.6, 25.9 (3C), 25.6 (3C), 22.6, 18.1, 18.0, -4.5 (2C), -4.8, -5.4

IR (neat) ν 2954, 2930, 2886, 2858, 1726, 1635, 1556, 1471, 1378, 1313, 1256, 1033, 839, 775, 699

HRMS (ESI): [M+Na]$^+$ calcd for $C_{29}H_{51}NO_5Si_2Na^+$: 572.3198, found: 572.3198

$[\alpha]_D^{25}$ +44.4 (*c* 1.010, CHCl$_3$)

R$_f$ (*n*-Hexane: EtOAc = 3:1, color reagent: *p*-anisaldehyde): 0.60

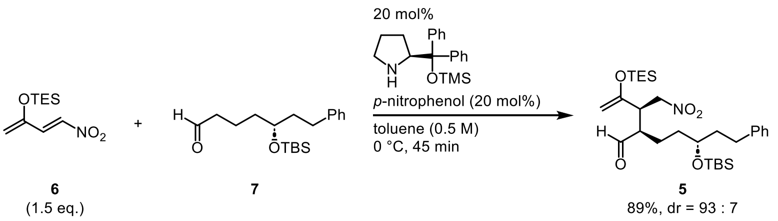

To a mixture of aldehyde **7** (1.05 g, 3.28 mmol, 1.0 eq.) in anhydrous toluene (5.25 mL) was added the toluene solution of nitroalkene **6** (43% in toluene, 2.62 g, 1.5 mmol, 1.5 eq.). The mixture was cooled to 0 °C, then *p*-nitrophenol (91.1 mg, 0.655 mmol, 20 mol%) and catalyst (213 mg, 0.655 mmol, 20 mol%) were added. After stirring for 45 min at 0 ℃, the reaction mixture was passed thorough the pad of silica and washed with ice cooled *n*-hexane/EtOAc = 20/1 and concentrated in vacuo. Purified by silica gel chromatography (*n*-Hexane/CH$_2$Cl$_2$ = 2 : 1 to *n*-Hexane/EtOAc = 30:1). Compound **5** was obtained in 89% yield (1.60 g).

(2*R*,5*R*)-5-((tert-butyldimethylsilyl)oxy)-2-((*R*)-1-nitro-3-((triethylsilyl)oxy)but-3-en-2-yl)-7-phenylheptanal

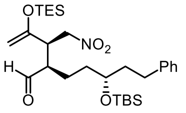

dr = 93 : 7

Physical State: yellow liquid

^{1}H NMR (400 MHz, CDCl$_3$) δ 9.65 (d, *J* = 2.8 Hz, 1H), 7.30-7.28 (m, 2H), 7.18-7.15 (m, 3H), 4.53 (dd, *J* = 12.0, 10.0 Hz, 1H), 4.42 (dd, *J* = 12.4, 4.4 Hz, 1H), 4.18 (d, *J* = 2.0 Hz, 1H), 4.14 (d, *J* = 2.0 Hz, 1H), 3.72-3.67 (m, 1H), 3.22 (td, *J* = 9.2, 4.4 Hz, 1H), 2.67-2.50 (m, 4H), 1.77-1.70 (m, 4H), 0.96 (t, *J* = 7.6 Hz, 9H), 0.90 (s, 9H), 0.71 (q, *J*

= 7.6 Hz, 6H), 0.05 (s, 3H), 0.02 (s, 3H)

^{13}C NMR (100 MHz, CDCl$_3$) δ 202.8, 153.2, 142.2, 128.3 (2C), 128.2 (2C), 125.7, 92.6, 75.5, 71.4, 50.8, 44.7, 38.5, 33.4, 31.6, 25.8 (3C), 22.4, 18.0, 6.6 (3C), 4.5 (3C), -4.5 (2C)

IR (neat) ν 2955, 2931, 2878, 2857, 1725, 1633, 1556, 1457, 1379, 1274, 1254, 1092, 1065, 1031, 1006, 836, 775, 736, 700

HRMS (ESI): [M+Na]$^+$ calcd for $C_{29}H_{51}NO_5Si_2Na^+$: 572.3198, found: 572.3198

$[\alpha]_D^{26}$ +32.7 (*c* 0.980, CHCl$_3$)

R$_f$ (*n*-Hexane: EtOAc = 3:1, color reagent: *p*-anisaldehyde): 0.60

Intramolecular Mukaiyama aldol reaction catalyzed by Me$_2$AlCl

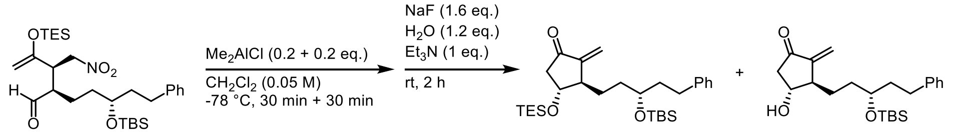

5 3 74% 17 23%

To a solution of compound **5** (1.15 g, 2.09 mmol, 1.0 eq.) (**5** were azeotropic with benzene three times.) in anhydrous CH$_2$Cl$_2$ (41.8 mL) was added Me$_2$AlCl (1.0 M in *n*-Hexane, 418 μL, 0.418 mmol, 0.20 eq.) in 10 min at -78 ℃. After stirring for 30 min at this temperature, additional Me$_2$AlCl (418 μL, 0.418 mmol, 0.20 eq.) was added in 10 min. After stirring for 30 min at this temperature, the reaction mixture was added NaF (141 mg, 3.35 mmol, 1.6 eq.) and water (45.2 μL, 2.51 mmol, 1.2 eq.), allowed to warm to room temperature. The resulting suspension was added Et$_3$N (291 μL, 2.09 mmol, 1.0 eq.). After stirring for 2 h at room temperature, the resulting suspension was filtered, washed with CH$_2$Cl$_2$ and the filtrate was concentrated in vacuo. Purification by silica gel chromatography (*n*-Hexane: Et$_2$O = 30:1 to 1:1) gave compound **3** and **17** in 74% (776 mg) and 23% (189 mg) yield respectively.

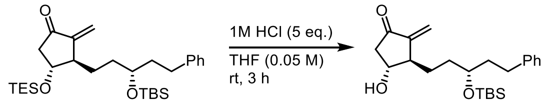

3 17 54%

To a solution of compound **3** (8.4 mg, 17 μmol, 1.0 eq.) in THF (0.33 mL) was added HCl (1 M in H$_2$O, 84 μL, 84 μmol, 5.0 eq.) at rt. After stirring for 30 min at this temperature, the reaction mixture was quenched by the addition of phosphorus buffer and extracted with Et$_2$O three times. The combined organic layer was washed with brine, dried with anhydrous MgSO$_4$, filtered and concentrated in vacuo. Purification by silica gel chromatography (*n*-Hexane: EtOAc = 9:1 to 3:1) gave compound **17** in 54% yield (3.5 mg).

(*3R*,*4R*)-4-((*tert*-butyldimethylsilyl)oxy)-3-((R)-3-((*tert*-butyldimethylsilyl)oxy)-5-phenylpentyl)-2-methylenecyclopentan-1-one

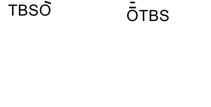

Physical State: yellow liquid

^{1}H NMR (400 MHz, CDCl$_3$) δ 7.30-7.28 (m, 2H), 7.20-7.16 (m, 3H), 6.08 (d, J = 2.0 Hz, 1H), 5.28 (d, J = 2.0 Hz, 1H), 4.09 (ddd, J = 4.8, 4.8, 4.8 Hz, 1H), 3.76-3.70 (m, 1H), 2.71-2.62 (m, 3H), 2.51 (dd, J = 18.0, 6.0, 1H), 2.30 (dd, J = 18.0, 4.8 Hz, 1H), 1.80-1.74 (m, 2H), 1.67-1.59 (m, 3H), 0.91 (s, 9H), 0.87 (s, 9H), 0.08 (s, 3H), 0.06 (s, 3H), 0.06 (s, 3H), 0.04 (s, 3H)

^{13}C NMR (100 MHz, CDCl$_3$) δ 204.5, 147.4, 142.4, 128.4 (2C), 128.3 (2C), 125.7, 118.1, 72.3, 71.8, 51.1, 46.9, 39.1, 34.1, 31.7, 27.7, 25.9 (3C), 25.7 (3C), 18.1, 17.9, -4.3, -4.4, -4.5, -4.8

IR (neat) ν 2953, 2929, 2857, 1732, 1645, 1468, 1255, 1098, 1065, 909, 836, 774, 738, 700

HRMS (ESI): [M+Na]$^+$ calcd for $C_{29}H_{50}O_3Si_2Na^+$: 525.3191, found: 525.3191

$[\alpha]_D^{29}$ -16.4 (*c* 1.540, CHCl$_3$)

R$_f$ (*n*-hexane/ EtOAc = 3:1, color reagent: *p*-anisaldehyde): 0.62

(3*R*,4*R*)-3-((*R*)-3-((tert-butyldimethylsilyl)oxy)-5-phenylpentyl)-2-methylene-4-((triethylsilyl)oxy)cyclopentan-1-one

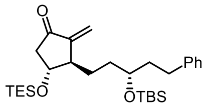

Physical State: yellow liquid

^{1}H NMR (400 MHz, CDCl$_3$) δ 7.30-7.27 (m, 2H), 7.20-7.17 (m, 3H), 6.09 (d, J = 2.0 Hz, 1H), 5.29 (dd, J = 2.4, 0.8 Hz, 1H), 4.10 (q, J = 6.0 Hz, 1H), 3.76-3.71 (m, 1H), 2.70-2.57 (5H), 2.31 (dd, J = 17.6, 4.8 Hz, 1H), 1.80-1.74 (m, 2H), 1.68-1.58 (m, 4H), 0.95 (t, J = 7.6 Hz, 9H), 0.91 (s, 9H), 0.60 (q, J = 7.6 Hz, 6H), 0.07 (s, 3H), 0.04 (s, 3H)

^{13}C NMR (100 MHz, CDCl$_3$) δ 204.5, 147.4, 142.4, 128.4 (2C), 128.3 (2C), 125.7, 118.2, 72.1, 71.7, 51.1, 46.9, 39.1, 34.1, 31.7, 27.7, 25.9 (3C), 18.1, 6.8 (3C), 4.8 (3C), -4.3, -4.4

IR (neat) ν 2954, 2932, 2877, 2857, 1733, 1254, 1095, 1067, 836, 774, 745

HRMS (ESI): [M+Na]$^+$ calcd for $C_{29}H_{50}O_3Si_2Na^+$: 525.3191, found: 525.3190

$[\alpha]_D^{29}$ -8.7 (*c* 1.540, CHCl$_3$)

R$_f$ (*n*-Hexane: EtOAc = 3:1, color reagent: *p*-anisaldehyde): 0.71

The enantiomeric ratio was determined by HPLC using CHIRALPACK® OD-H (*n*-Hexane: *i*-PrOH = 2000:1; flow rate 1.0 mL/min, major isomer t_R = 15 min, minor isomer t_R = 14 min) (>99% *ee*)

The diastereomeric ratio was determined after the deprotection of TES group.

(3*R*,4*R*)-3-((*R*)-3-((tert-butyldimethylsilyl)oxy)-5-phenylpentyl)-4-hydroxy-2-methylenecyclopentan-1-one

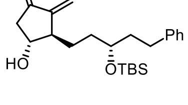

Physical State: yellow oil

^{1}H NMR (400 MHz, CDCl$_3$) δ 7.30-7.27 (m, 2H), 7.20-7.17 (m, 3H), 6.13 (d, J = 2.0 Hz, 1H), 5.33 (d, J = 2.0 Hz, 1H), 4.22-4.28 (m, 1H), 3.77 (m, 1H), 2.73-2.56 (m, 5H), 2.37 (dd, J = 17.6, 4.0 Hz, 1H), 1.80-1.75 (m, 3H), 1.68-

1.59 (m, 4H), 0.90 (s, 9H), 0.07 (s, 3H), 0.04 (s, 3H)

^{13}C NMR (100 MHz, $CDCl_3$) δ 204.0, 147.2, 142.4, 128.4 (2C), 128.3 (2C), 125.8, 118.9, 72.0, 71.6, 50.5, 46.2, 38.9, 38.8, 33.9, 27.8, 25.9 (3C), 18.1, -4.3, -4.4

IR (neat) ν 2951, 2928, 2856, 1730, 1254, 1092, 1065, 835, 774, 699

HRMS (ESI): $[M+Na]^+$ calcd for $C_{23}H_{36}O_3SiNa^+$: 411.2326, found: 411.2327

$[\alpha]_D^{29}$ +6.9 (*c* 1.110, $CHCl_3$)

R_f (*n*-Hexane: EtOAc = 3:1, color reagent: *p*-anisaldehyde): 0.13

The diastereomeric ratio was determined by HPLC using CHIRALPACK® AS-H (*n*-Hexane: *i*-PrOH = 99:1; flow rate 1.0 mL/min, major isomer t_R = 37 min, minor isomer t_R = 26 min) (dr = 97.3 : 2.6, dr = 81.6 : 18.4)

Addition of vinyl cuprate

Optimization

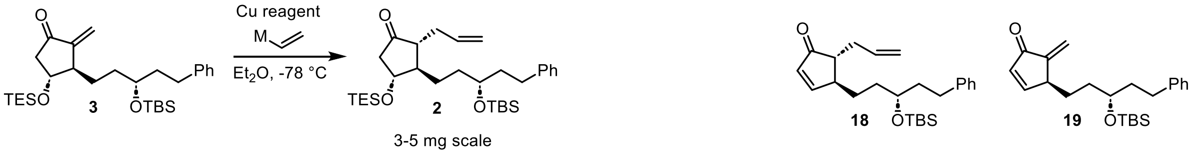

Entry	Cu reagent [eq.]	M [eq.]	Additive [eq.]	Time [min]	Yield [%]	Comments
1	CuI [7.5]	MgBr [15]	-	240	n.d.	no reaction
2	CuI [7.5]	MgBr [15]	PBu_3 [8]	20	40	-
3	$[CuI(PBu_3)]_4$ [1.25]	Li [10]	-	10	68	**18** : ~17%[c]
4	$[CuI(PBu_3)]_4$ [1.25]	Li [10]	TMSCl [5]	60	25	**18** : ~20%[c], **19** : ~28%[c]
5	$[CuI(PBu_3)]_4$ [1.25]	Li [10]	$BF_3 \cdot OEt_2$ [5]	10	74	-
6[a]	$[CuI(PBu_3)]_4$ [0.5]	Li [4]	$BF_3 \cdot OEt_2$ [2]	10	90	-
7[b]	$[CuI(PBu_3)]_4$ [0.5]	Li [4]	$BF_3 \cdot OEt_2$ [2]	10	83	-

[a] 50 mg scale. [b] 1.00 g scale. [c] estimated by crude NMR.

$[CuI(PBu)_3]_4$ and vinyl lithium were prepared according to reported procedure.[1,2)]

To a solution Cu reagent in anhydrous Et_2O (0.15 mL) was added vinyl metal reagent slowly at -45 °C. After stirring for 30 min at this temperature, the reaction mixture was cooled to -78 °C and added additive. the resulting mixture was added compound **3** (3.0 mg, 6.0 μmol) in anhydrous Et_2O (50 μL) in 5 min. After stirring for indicated time, the reaction mixture was quenched by the addition of phosphorus buffer and extracted with Et_2O three times. The combined organic layer was washed with sat. NH_4Cl aq., water and brine, dried with anhydrous $MgSO_4$, filtered and concentrated in vacuo. Purification by silica gel chromatography (*n*-Hexane: CH_2Cl_2 = 2 : 1 to *n*-Hexane: EtOAc = 30 : 1) gave compound **2**.

The yield of **18** and **19** was estimated by crude NMR (entries 3, 4) and the crude NMR of entries 3, 4, 7 were shown later page.

Optimized reaction procedure (entry 7)

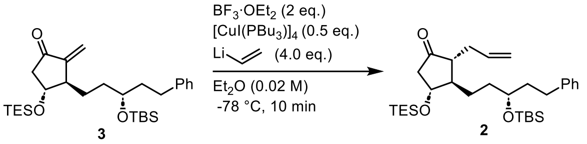

To a solution $[CuI(PBu)_3]_4$ (1.56 g, 994 μmol, 0.50 eq.) in anhydrous Et_2O (66.3 mL) was added vinyl lithium (0.84 M in Et_2O, 9.51 mL, 7.95 mmol, 4.0 eq.) slowly (over 25 min, the color of solution was changed colorless to brown, then yellow) at -45 °C. After stirring for 30 min at this temperature, the reaction mixture was cooled to -78 °C and added $BF_3{\cdot}OEt_2$ (565 μL, 3.98 mmol, 2 eq.) in anhydrous Et_2O (11.1 mL). the resulting mixture was added compound **3** (1.00 g, 1.99 mmol, 1.0 eq.) (**3** were azeotropic with benzene three times.) in anhydrous Et_2O (22.1 mL) in 15 min. After stirring for 10 min, the reaction mixture was quenched by the addition of phosphorus buffer and extracted with Et_2O three times. The combined organic layer was washed with sat. NH_4Cl aq., water and brine, dried with anhydrous $MgSO_4$, filtered and concentrated in vacuo. Purification by silica gel chromatography (*n*-Hexane: CH_2Cl_2 = 2 : 1 to *n*-Hexane: EtOAc = 30 : 1) gave compound **2** in 83% yield (878 mg).

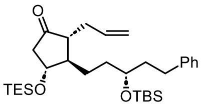

Physical State: yellow liquid

^{1}H NMR (400 MHz, $CDCl_3$) δ 7.30-7.26 (m, 2H), 7.70-7.16 (m, 3H), 5.78-5.67 (m, 1H), 5.11-5.03 (m, 2H), 4.06 (q, J = 6.4 Hz, 1H), 3.74-3.68 (m, 1H), 2.72-2.56 (m, 3H), 2.43-2.39 (m, 2H), 2.17 (dd, J = 6.0, 18.0 Hz, 1H), 1.97-1.89 (m, 2H), 1.79-1.74 (m, 2H), 1.62-1.42 (m, 4H), 0.97-0.89 (m, 18H), 0.63-0.57 (m, 6H), 0.07-0.05 (6H, m)

^{13}C NMR (100 MHz, $CDCl_3$) δ 217.2, 142.5, 135.6, 128.4 (2C), 128.3 (2C), 125.7, 117.1, 73.1, 72.0, 53.0, 48.9, 47.7, 39.0, 34.3, 33.8, 31.7, 27.8, 25.9 (3C), 18.1, 6.8 (3C), 4.8 (3C), -4.4 (2C)

IR (neat) ν 3060, 3028, 2956, 1742, 1641, 1603, 1496, 1458, 1415, 1377, 1264, 1097, 1006, 940, 918, 836, 775, 737

HRMS (ESI): $[M+Na]^+$ calcd for $C_{31}H_{54}O_3Si_2Na^+$: 553.3504, found: 553.53521

$[\alpha]_D^{24}$ -31.0 (*c* 0.44, $CHCl_3$)

R_f (*n*-Hexane: EtOAc = 9 : 1, color reagent: *p*-anisaldehyde): 0.46

Total synthesis of latanoprost

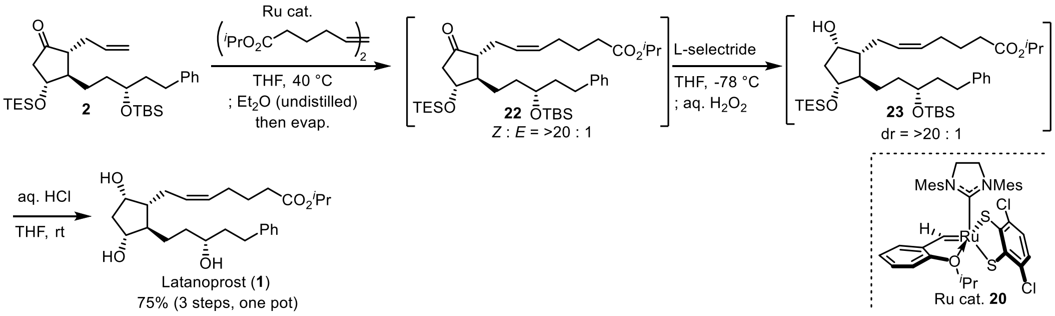

0.06 M THF solution of Ru cat. **20** was prepared according to the reported procedure.[3)]

A solution of compound **2** (9.8 mg, 18.5 μmol) and diester **21** (21.0 mg, 73.8 μmol, 4 eq.) (**2** and **21** were azeotropic with benzene three times) and anhydrous THF (0.37 mL) was added to the Schrenk flask and freeze-pump-thaw for three times. Ru catalyst **20** (0.06 M in THF, 61.5 μL, 3.69 μmol, 20 mol%) was added at rt and reaction mixture was warmed to 40 °C. After stirring at 40 °C for 9.5 h, additional Ru catalyst **20** (0.06 M in THF, 61.5 μL, 3.69 μmol, 20 mol%) was added to the reaction mixture. After stirring reaction mixture at 40 °C for 13 h, undistilled Et_2O (1 mL) was added to the mixture, then concentrated in vacuo. Remaining mixture was dissolved to anhydrous THF (0.37 mL) and cooled to -78 °C and L-selectride® (1 M in THF, 92.3 μL, 92.3 μmol, 5 eq.) was added slowly (over 5 min). After stirring this temperature for 30 min, H_2O_2 (30% in H_2O, 92.3 μL) was added to the mixture and warmed to room temperature. THF (0.37 mL) and 1M HCl aq. (0.37 mL, 20 eq.) were added and stirred at this temperature. After stirring for 22 h, the reaction mixture was quenched by the addition of phosphate buffer and extracted with EtOAc three times. The combined organic layer was washed with brine, dried with anhydrous Na_2SO_4, filtered and concentrated in vacuo. Purification by silica gel chromatography (*n*-Hexane: EtOAc = 1 : 1 to 1 : 3) gave latanoprost **1** in 75% yield (6.0 mg).

Latanoprost

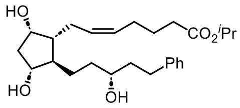

known compound

Physical State: pale yellow oil

^{1}H NMR (400 MHz, $CDCl_3$) δ 7.31–7.26 (m, 2H), 7.22–7.15 (m, 3H), 5.50–5.35 (m, 2H), 5.00 (sept, J = 6.4 Hz, 1H), 4.17 (br s, 1H), 3.94 (br s, 1H), 3.70–3.64 (m, 1H), 2.85–2.63 (m, 2H), 2.36–2.08 (m, 6H), 1.90–1.30, (m, 14H), 1.23 (d, J = 6.4 Hz, 6H)

^{13}C NMR (100 MHz, $CDCl_3$) δ 173.5, 142.0, 129.6, 129.3, 128.4 (4C), 125.8, 78.7, 74.9, 71.7, 67.7, 52.8, 52.0, 42.3, 39.3, 35.8, 34.0, 32.0, 30.3, 26.9, 26.6, 24.9, 21.8 (2C)

HRMS (ESI): $[M+Na]^+$ calcd for $C_{26}H_{40}O_5Na^+$: 455.2768, found: 455.2756

$[\alpha]_D^{26}$ +30.8 (*c* 0.03, CH_3CN), lit. $[\alpha]_D^{20}$ +32.7 (*c* 1.03, MeCN)[4)]

R_f (*n*-Hexane: EtOAc = 1 : 3, color reagent: *p*-anisaldehyde): 0.26

Reference

1) G. B. Kauffman, L. A. Teter, *Inorg. Synth.* **1963**, *7*, 9.

2) A. P. Pulis, D. J. Blair, E. Torres and V. K. Aggarwal, *J. Am. Chem. Soc.* **2013**, *135*, 16054.

3) D. S. Müller, I. Curbet, Y. Raoul, J. L. Nôtre, O. Baslé and M. Mauduit, *Org. Lett.*, **2018**, *20*, 6822.

4) G. Zanoni, A. D'Alfonso, A. Porta, L. Feliciani, S. P. Nolan, G. Vidari, *Tetrahedron*, **2010**, *66*, 7472.

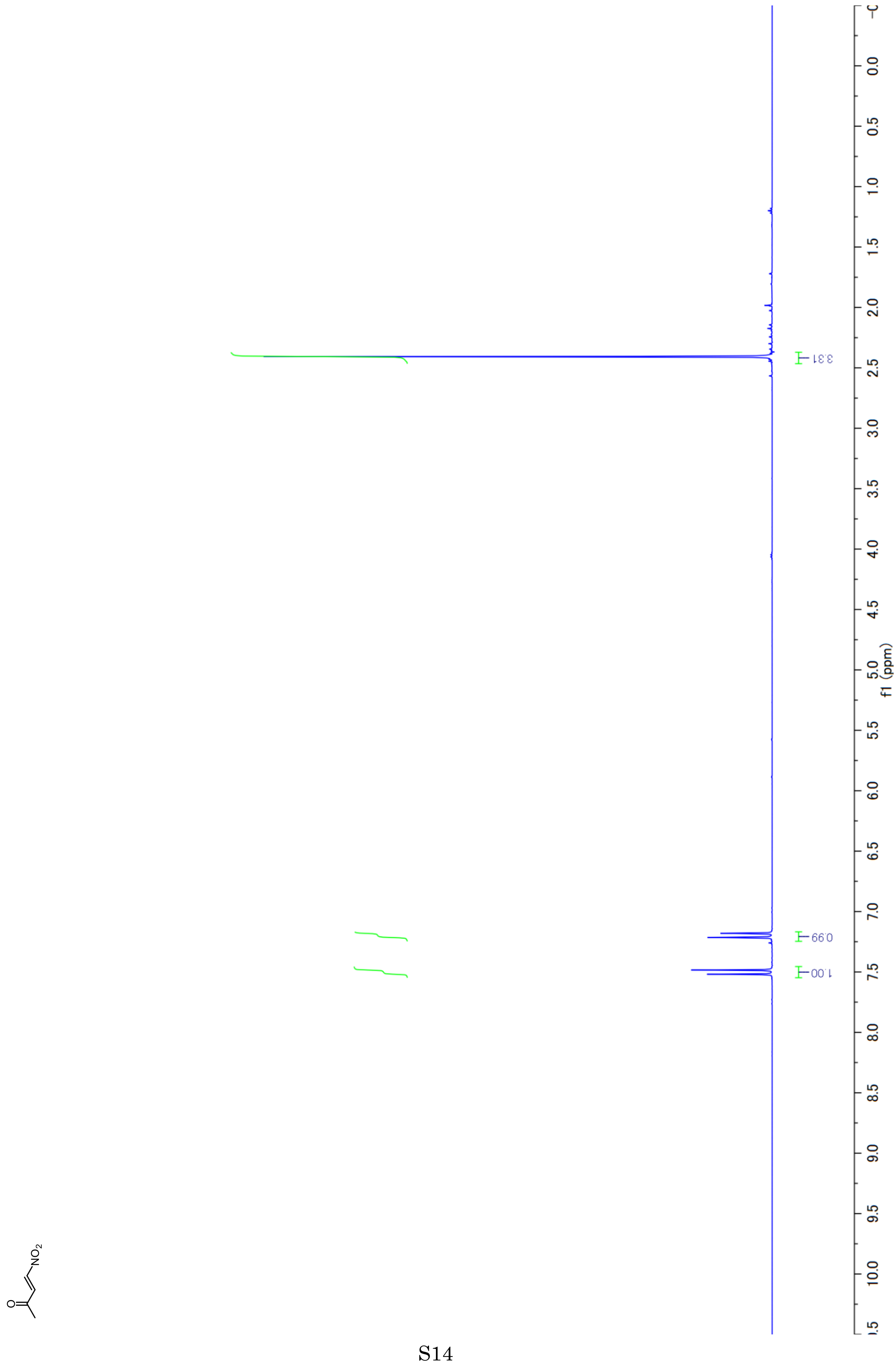

NO2
3.31
0.99
1.00
f1 (ppm)

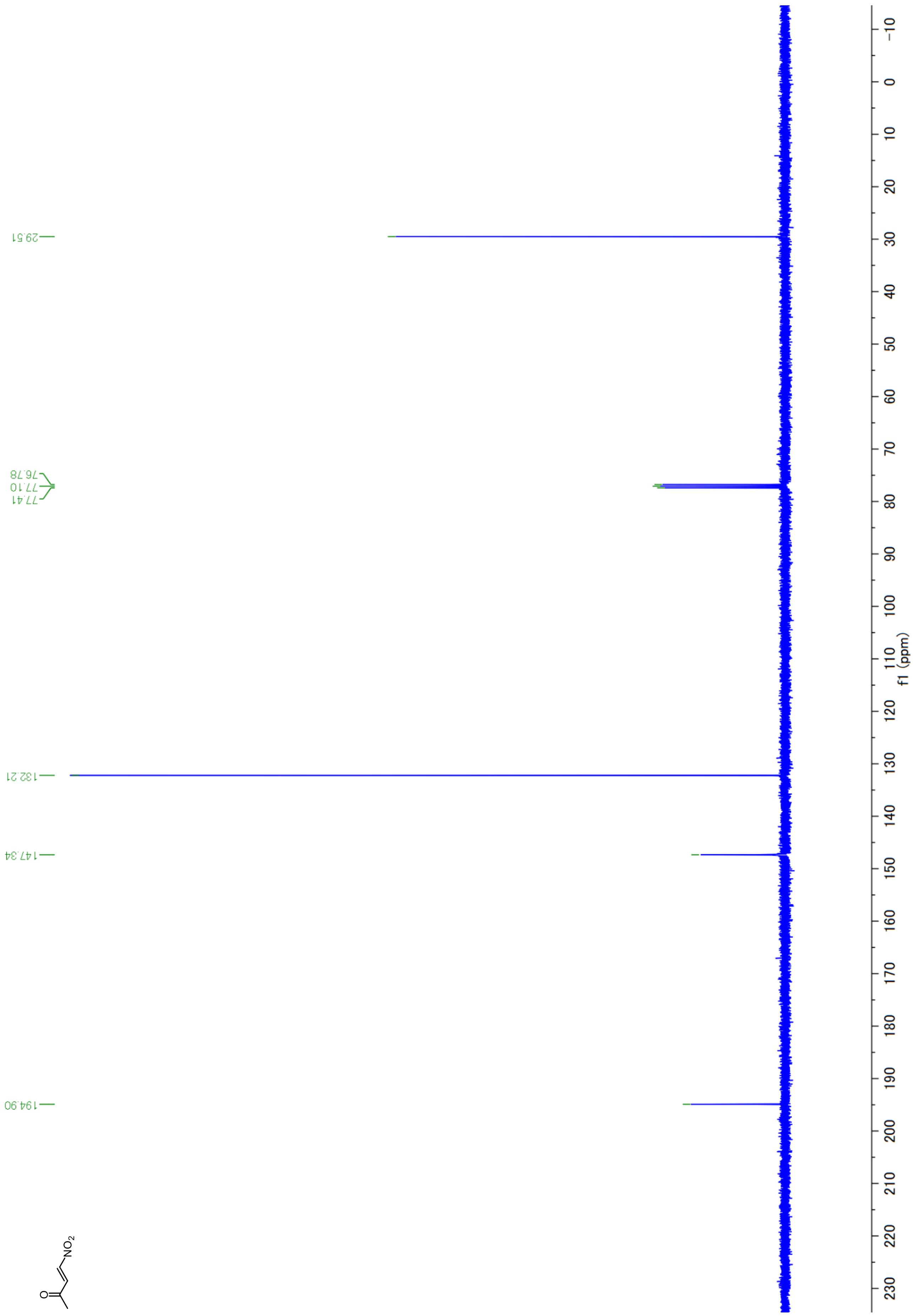
29.51
77.41
77.10
76.78
132.21
147.34
194.90
f1 (ppm)
O
NO2

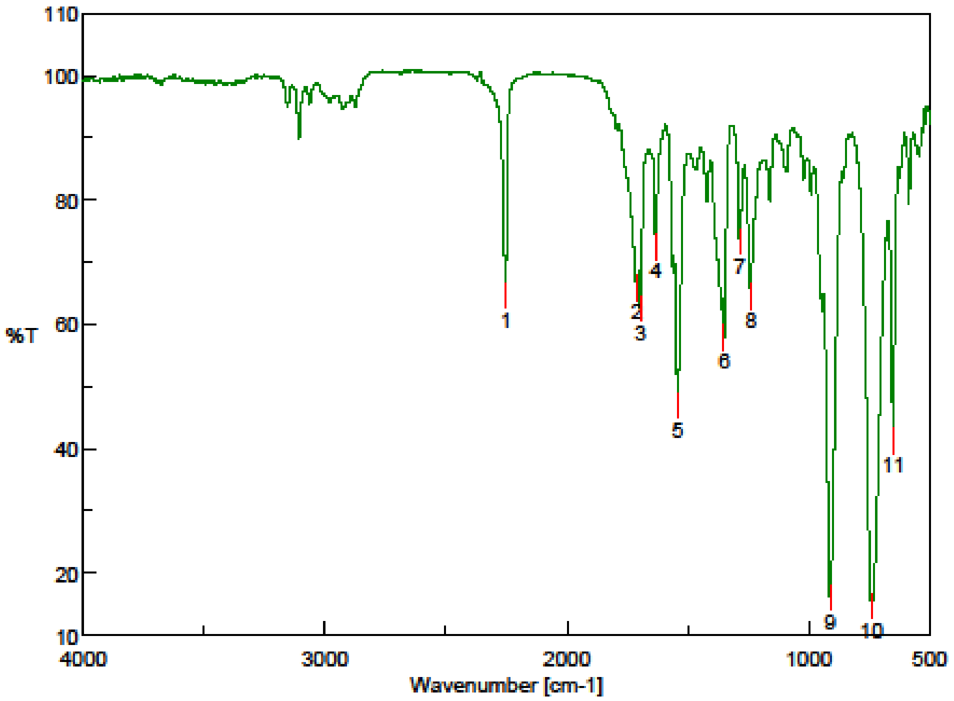

[ピーク検出結果]

No.	位置	強度	No.	位置	強度
1	2254.38	64.5463	2	1710.55	65.7703
3	1697.05	62.607	4	1632.45	72.4557
5	1541.81	46.9146	6	1349.93	57.9499
7	1285.32	73.2492	8	1240	64.4148
9	912.165	16.0714	10	740.531	14.6305
11	650.858	41.1381			

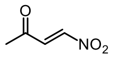

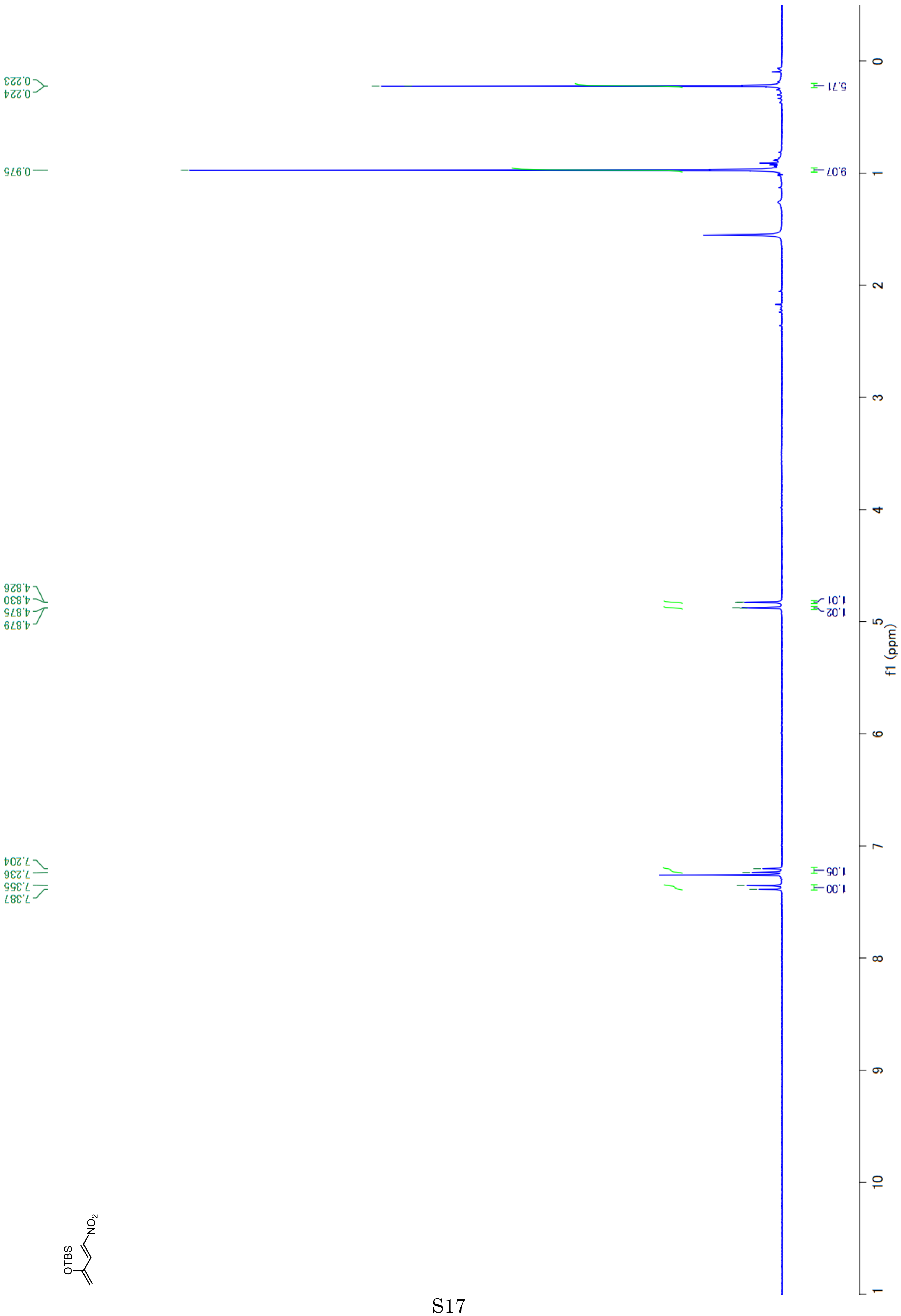

0.223
0.224
0.975
4.826
4.830
4.875
4.879
7.204
7.236
7.355
7.387
5.71
9.07
1.01
1.02
1.05
1.00
f1 (ppm)
OTBS
NO2

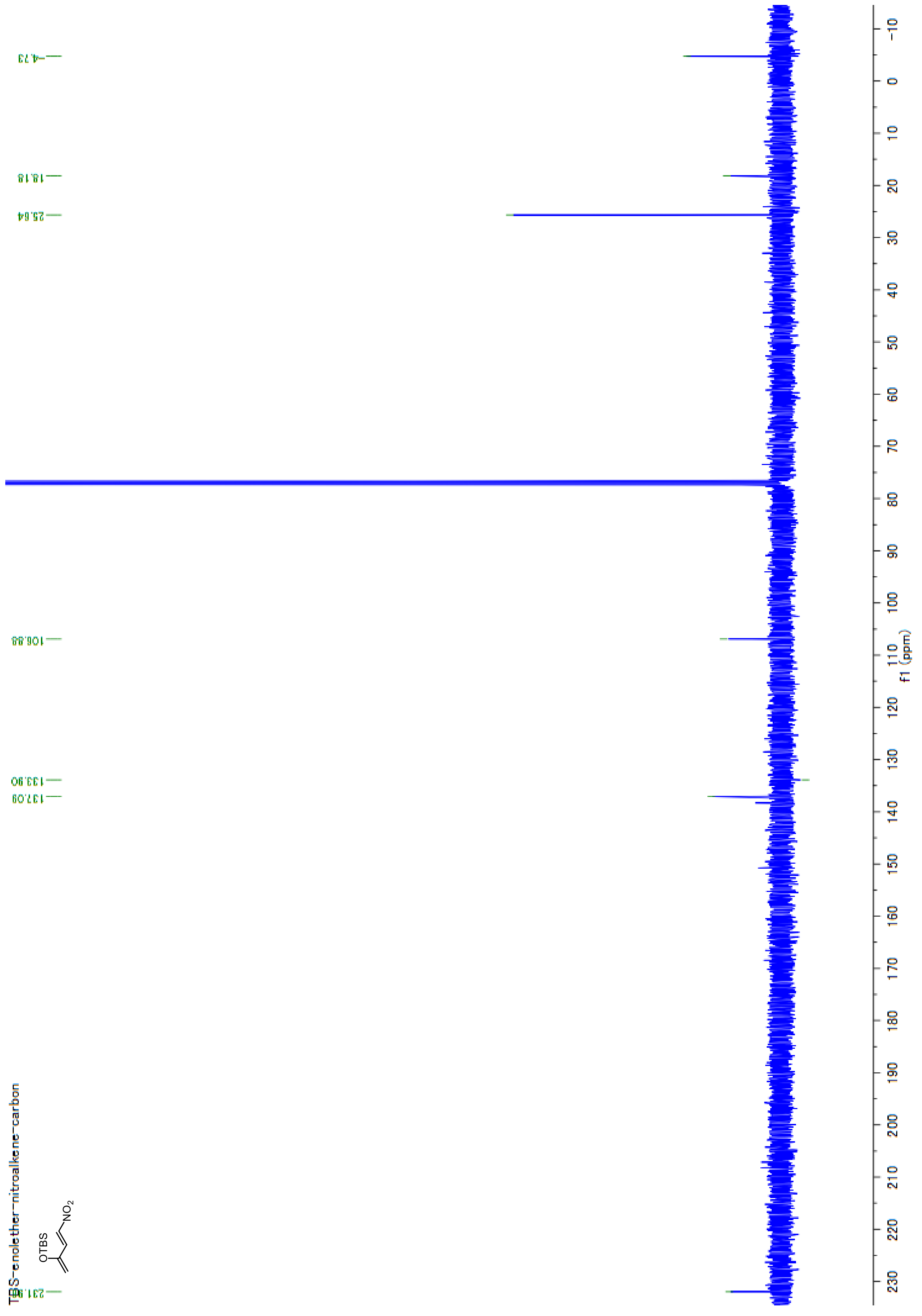
TBS-enolether-nitroalkene-carbon
OTBS
NO_2
-4.73
18.18
25.64
106.88
133.90
137.09
f1 (ppm)

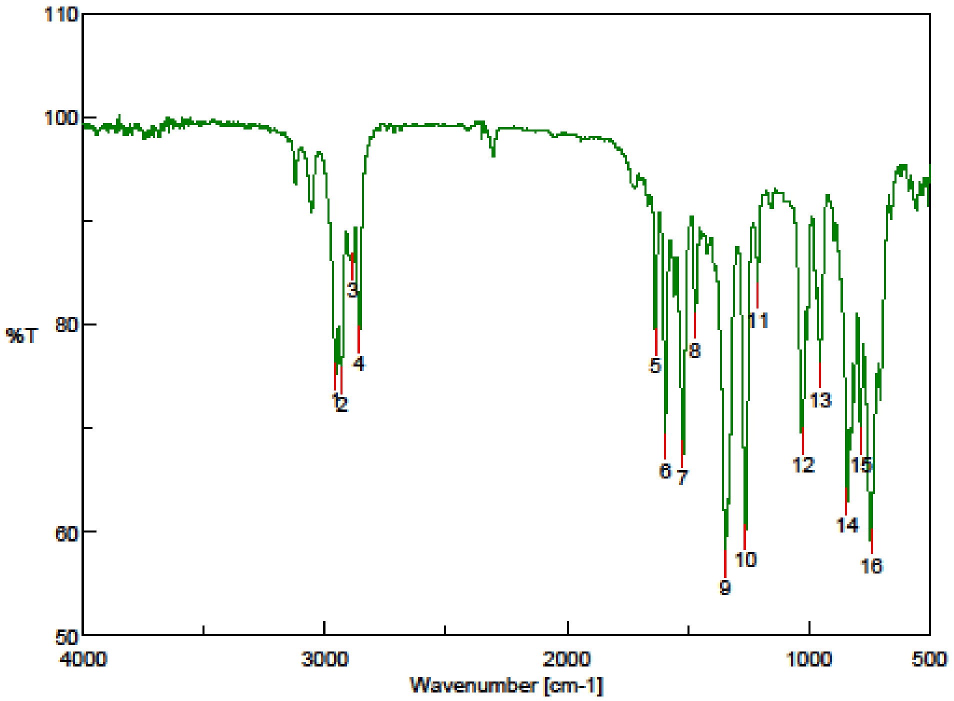

[ピーク検出結果]

No.	位置	強度	No.	位置	強度
1	2957.3	74.9934	2	2932.23	74.5682
3	2886.92	85.6297	4	2859.92	78.4563
5	1633.41	78.2852	6	1593.88	68.2127
7	1519.63	67.4483	8	1472.38	79.8557
9	1345.11	56.8591	10	1263.15	59.5051
11	1211.08	82.792	12	1025.94	68.6782
13	953.627	75.0556	14	841.776	62.9122
15	784.886	68.6799	16	742.46	59.0273

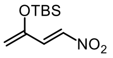

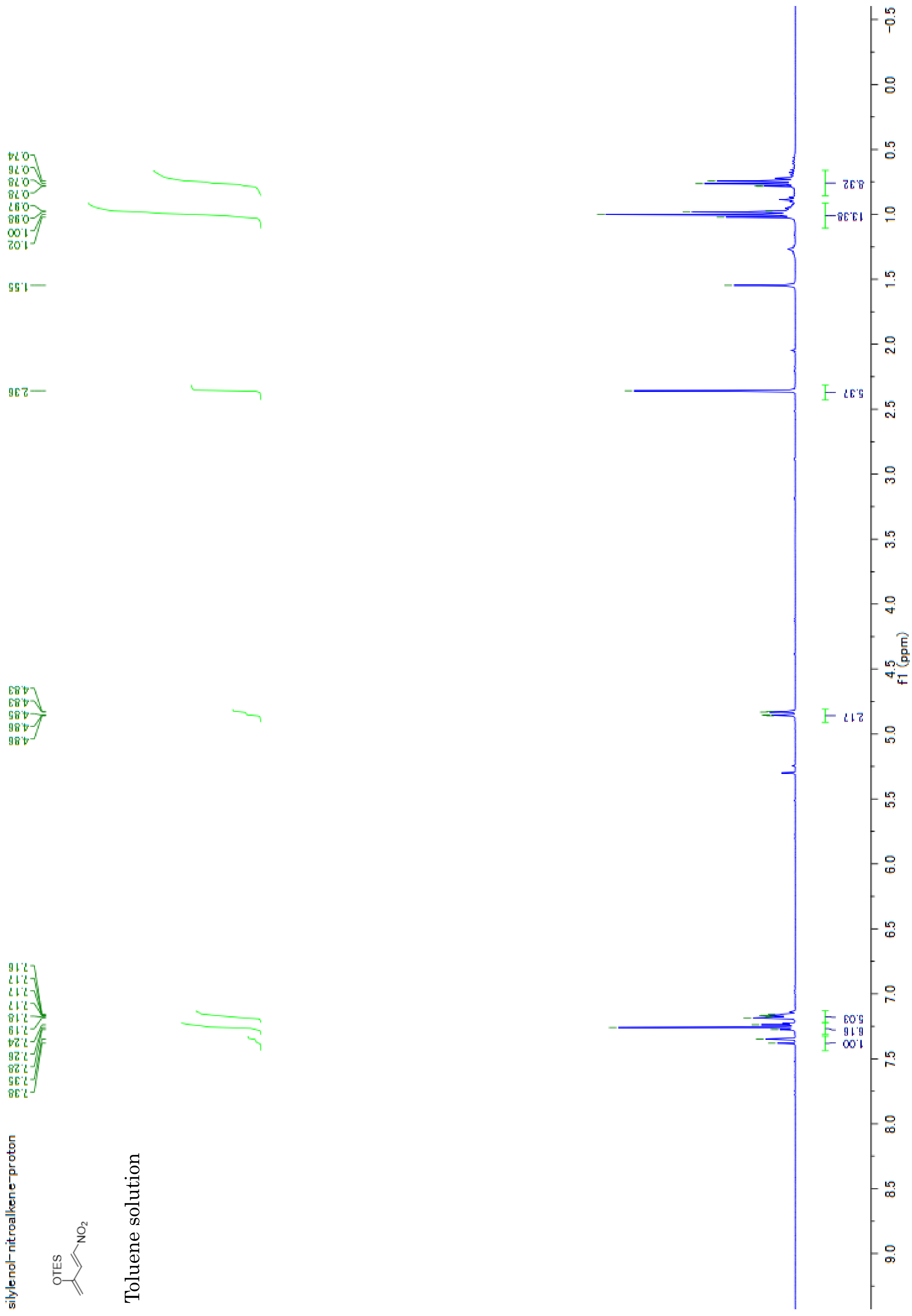
silylenol-nitroalkene-proton
OTES
NO2
Toluene solution
f1 (ppm)

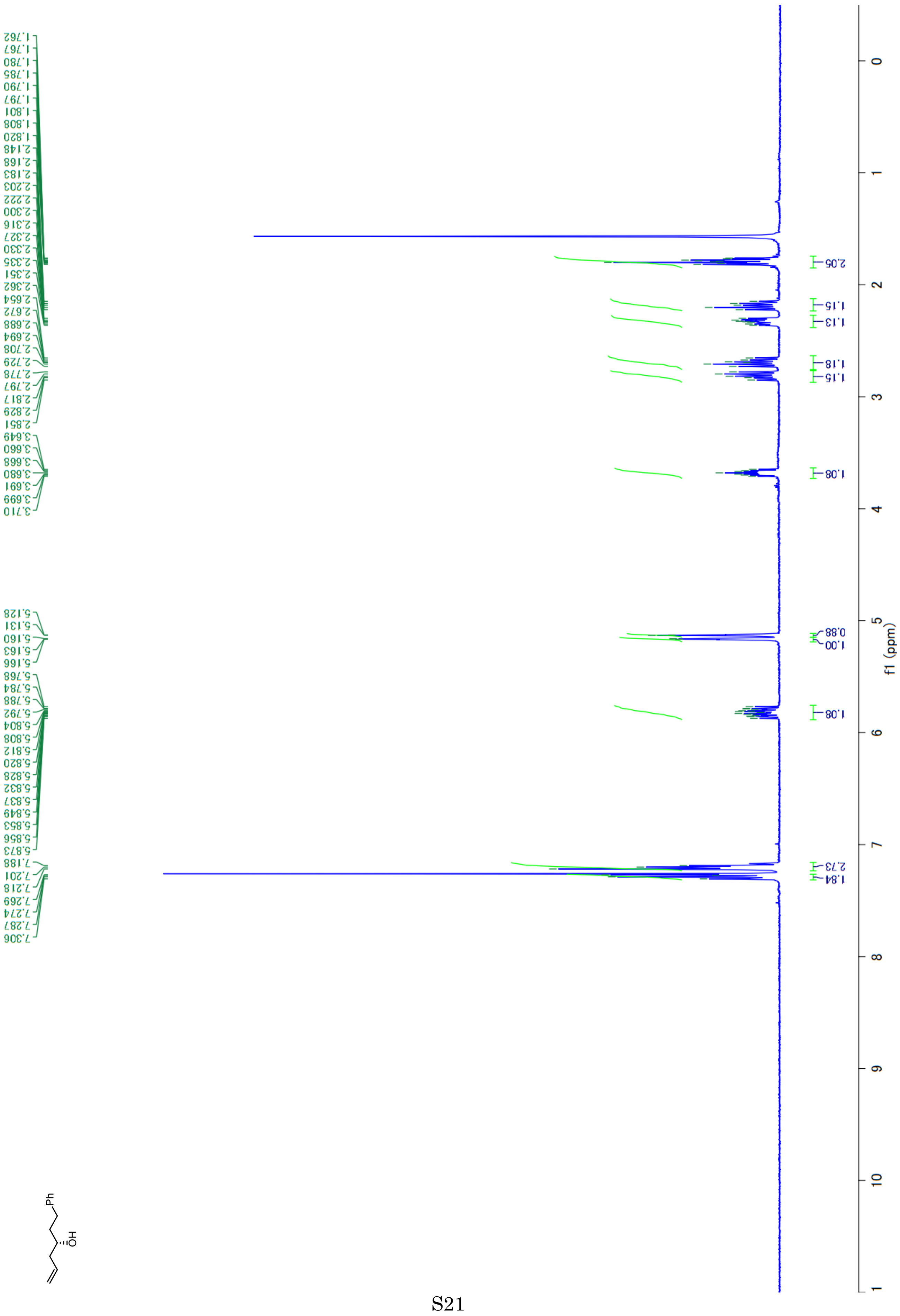
Ph
OH
1.762
1.767
1.780
1.785
1.790
1.797
1.801
1.808
1.820
2.148
2.168
2.183
2.203
2.222
2.300
2.316
2.327
2.330
2.335
2.351
2.362
2.654
2.672
2.688
2.694
2.708
2.729
2.778
2.797
2.817
2.829
2.851
3.649
3.660
3.668
3.680
3.691
3.699
3.710
5.128
5.131
5.160
5.163
5.166
5.768
5.784
5.788
5.792
5.804
5.808
5.812
5.820
5.828
5.832
5.837
5.849
5.853
5.856
5.873
7.188
7.201
7.218
7.269
7.274
7.287
7.306
2.05
1.15
1.13
1.18
1.15
1.08
0.88
1.00
1.08
2.73
1.84
f1 (ppm)

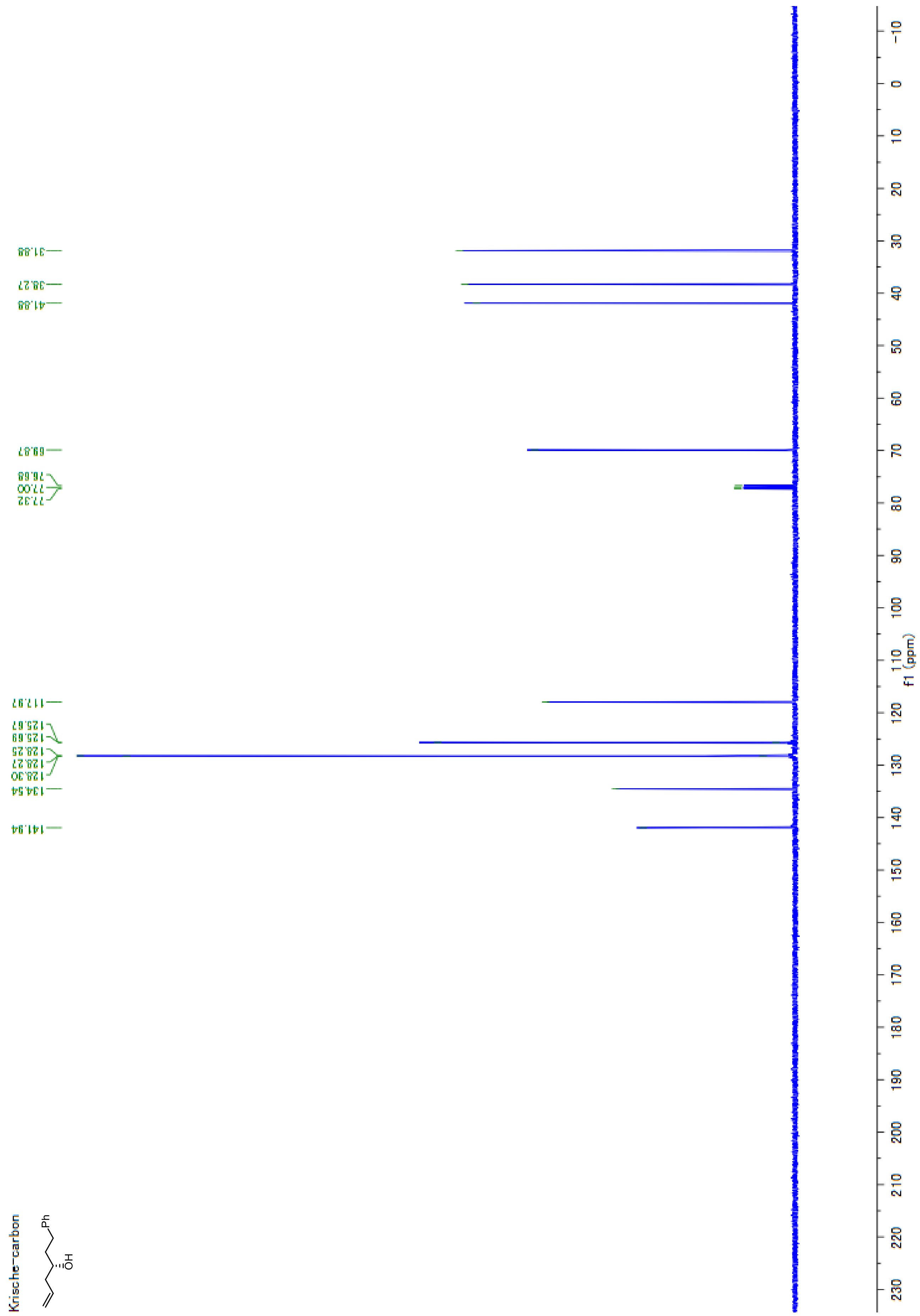
Krische-carbon
OH
Ph
141.94
134.54
128.30
128.27
128.25
125.69
125.67
117.97
77.32
77.00
76.68
69.87
41.88
38.27
31.88
f1 (ppm)

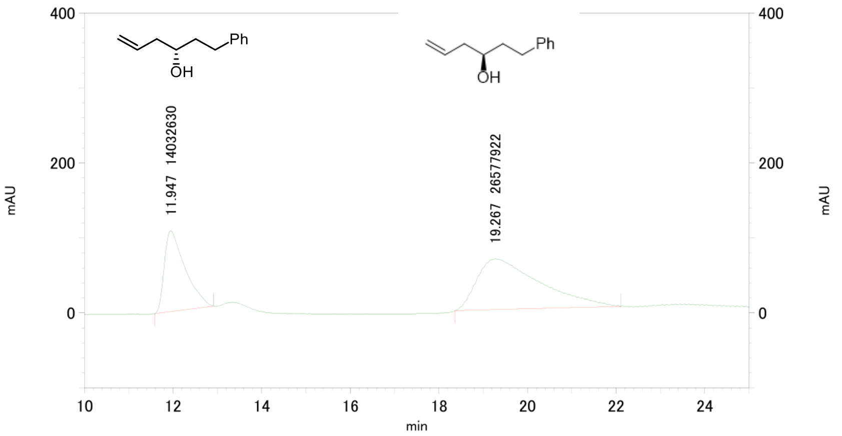

Result
1: 210 nm, 4 nm結果

Pk #	Retention time / min	Integration/ %
1	11.947	34.554
2	19.267	65.446
トータル		100.000
Total		

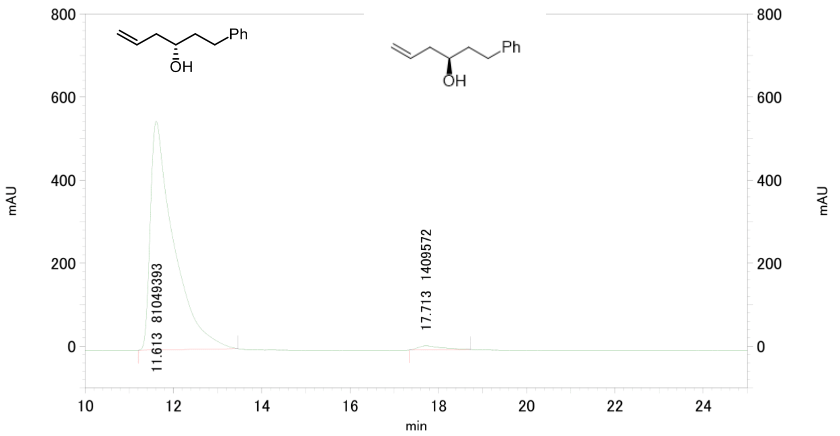

Result
1: 210 nm, 4 nm結果

Pk #	Retention time / min	Integration/ %
1	11.613	98.291
2	17.713	1.709
トータル		100.000
Total		

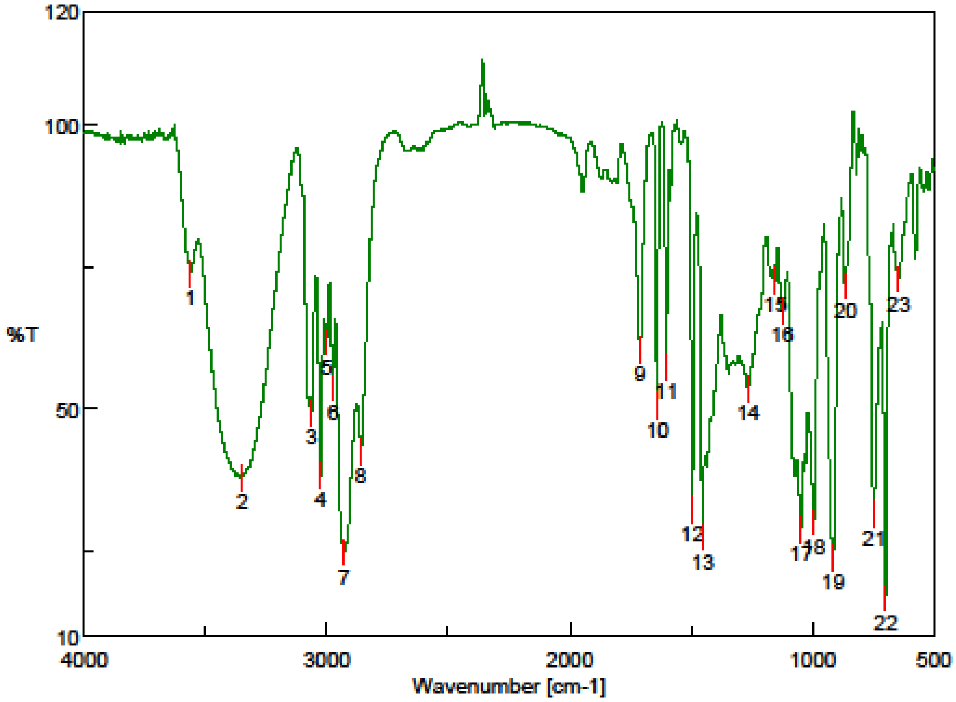

[ピーク検出結果]

No.	位置	強度	No.	位置	強度
1	3563.81	73.7693	2	3351.68	37.8263
3	3063.37	49.3237	4	3026.73	38.2094
5	3002.62	61.6704	6	2976.59	53.6172
7	2930.31	24.7289	8	2860.88	42.4297
9	1711.51	60.3626	10	1641.13	50.5014
11	1602.56	57.3764	12	1495.53	32.2052
13	1454.06	27.3404	14	1266.04	53.4044
15	1154.19	72.5597	16	1126.22	67.2882
17	1048.12	28.6954	18	995.089	29.9777
19	916.022	23.6867	20	864.917	71.6667
21	747.281	31.3747	22	700.034	16.715
23	646.036	72.8173			

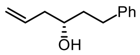

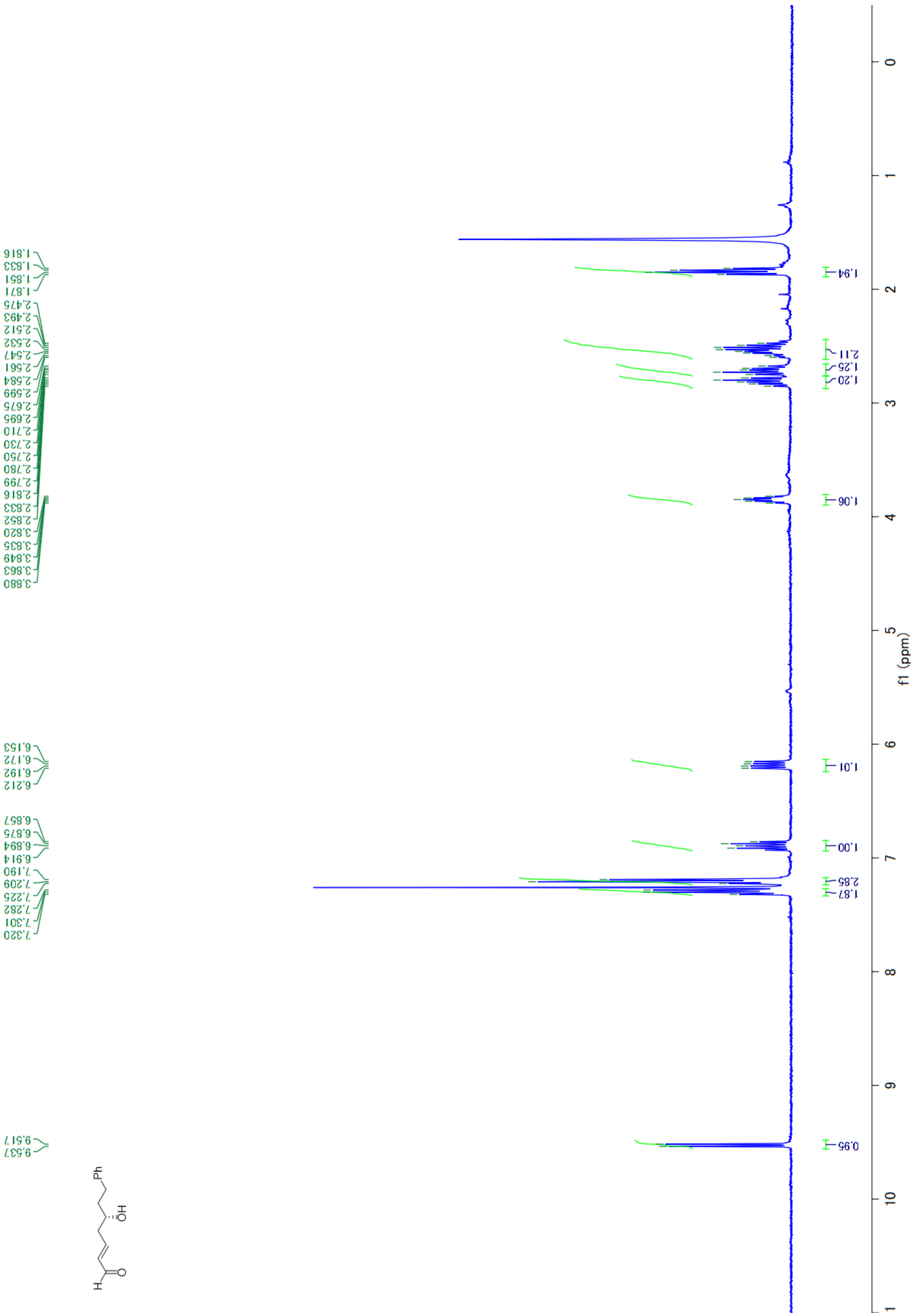

1.816
1.833
1.851
1.871
2.475
2.493
2.512
2.532
2.547
2.561
2.584
2.599
2.675
2.695
2.710
2.730
2.750
2.780
2.799
2.816
2.833
2.852
3.820
3.835
3.849
3.863
3.880
6.153
6.172
6.192
6.212
6.857
6.875
6.894
6.914
7.190
7.209
7.225
7.282
7.301
7.320
9.517
9.537
H
O
OH
Ph
0.95
1.87
2.85
1.00
1.01
1.06
1.20
1.25
2.11
1.94
f1 (ppm)
0
1
2
3
4
5
6
7
8
9
10
11

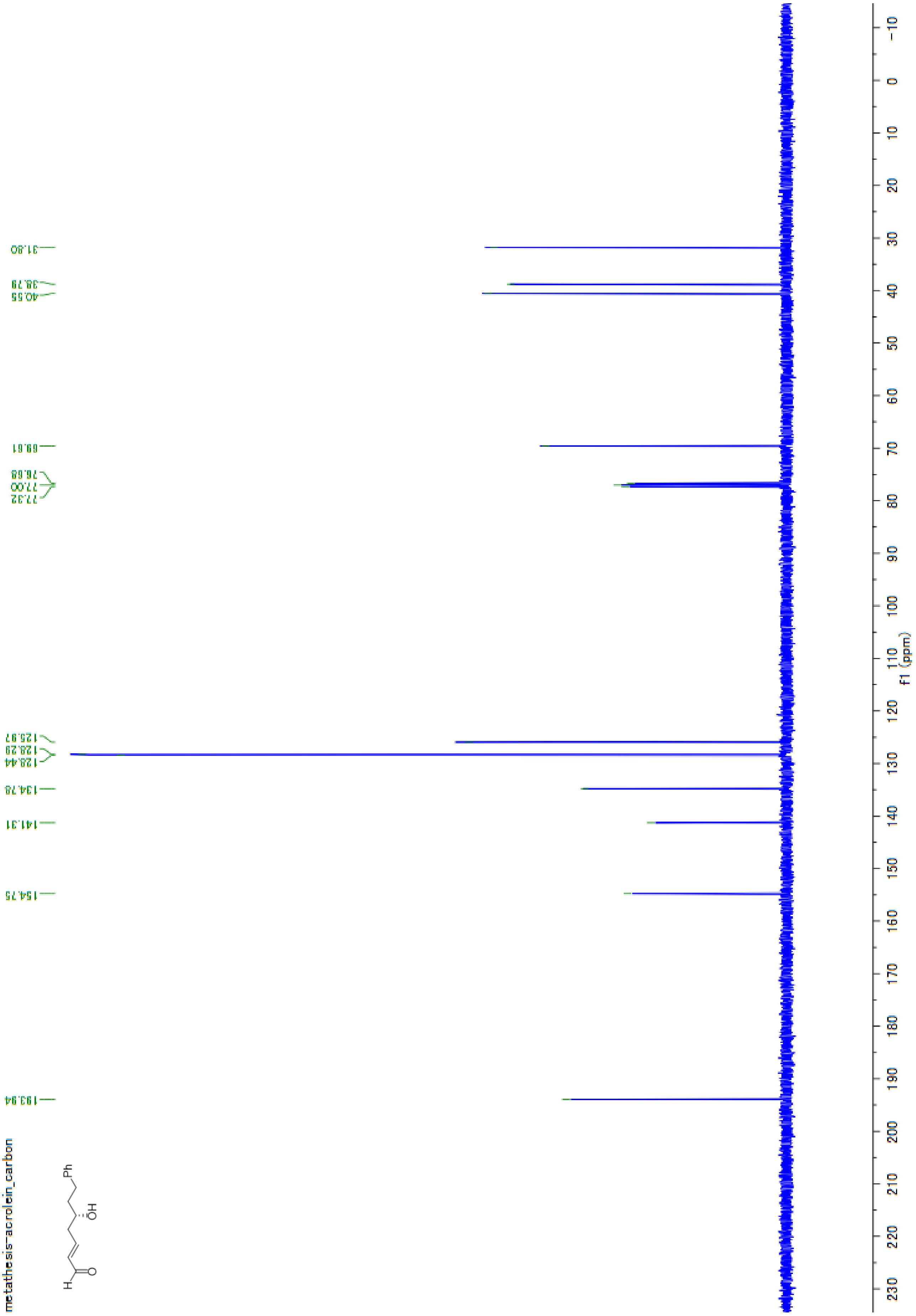
metathesis-acrolein_carbon
193.94
154.75
141.31
134.78
128.44
128.29
125.97
77.32
77.00
76.69
69.61
40.55
38.79
31.80
f1 (ppm)
Ph
OH
H
O

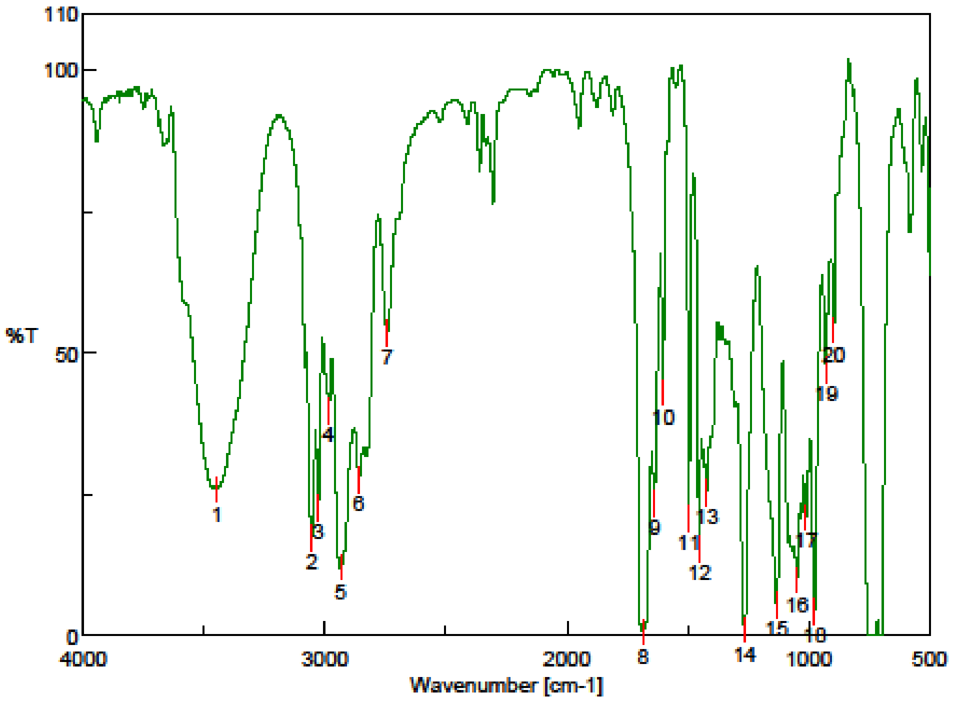

[ピーク検出結果]

No.	位置	強度	No.	位置	強度
1	3447.13	25.7086	2	3055.66	17.1215
3	3028.66	22.4544	4	2986.23	39.6233
5	2936.09	11.9679	6	2860.88	27.5279
7	2744.21	53.4862	8	1684.52	0.623924
9	1638.23	23.3397	10	1602.56	42.9173
11	1495.53	20.5119	12	1454.06	15.2369
13	1421.28	25.2412	14	1266.04	1.00845
15	1133.94	5.33556	16	1049.09	9.78159
17	1011.48	20.8949	18	975.804	4.17893
19	928.557	46.8865	20	896.737	53.9582
21	698.105	0			

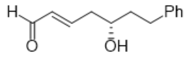

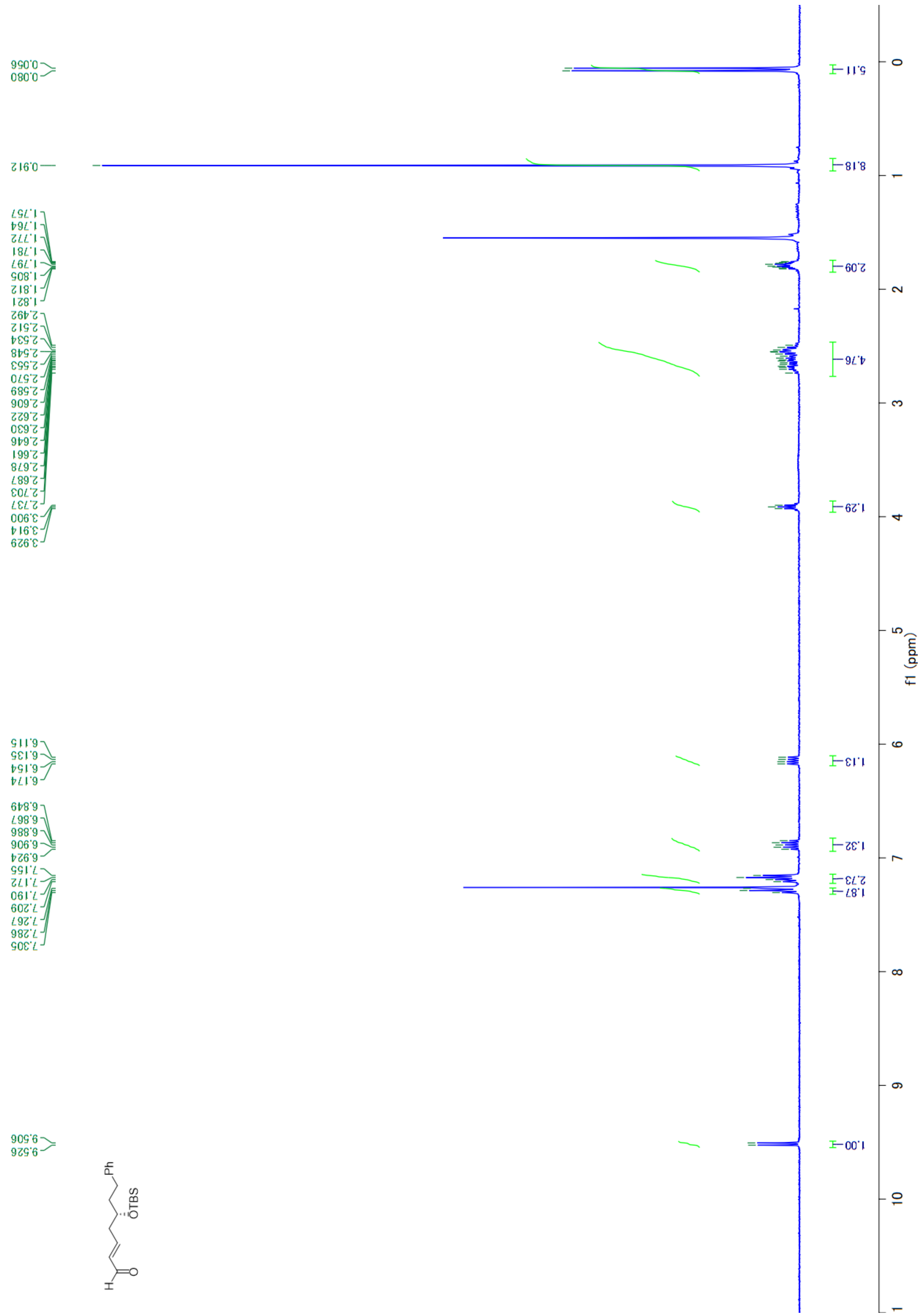
9.526
9.506
7.305
7.286
7.267
7.209
7.190
7.172
7.155
6.924
6.906
6.886
6.867
6.849
6.174
6.154
6.135
6.115
3.929
3.914
3.900
2.737
2.703
2.687
2.678
2.661
2.646
2.630
2.622
2.606
2.589
2.570
2.553
2.548
2.534
2.512
2.492
1.821
1.812
1.805
1.797
1.781
1.772
1.764
1.757
0.912
0.080
0.066
1.00
1.87
2.73
1.32
1.13
1.29
4.76
2.09
8.18
5.11
f1 (ppm)
H
O
OTBS
Ph

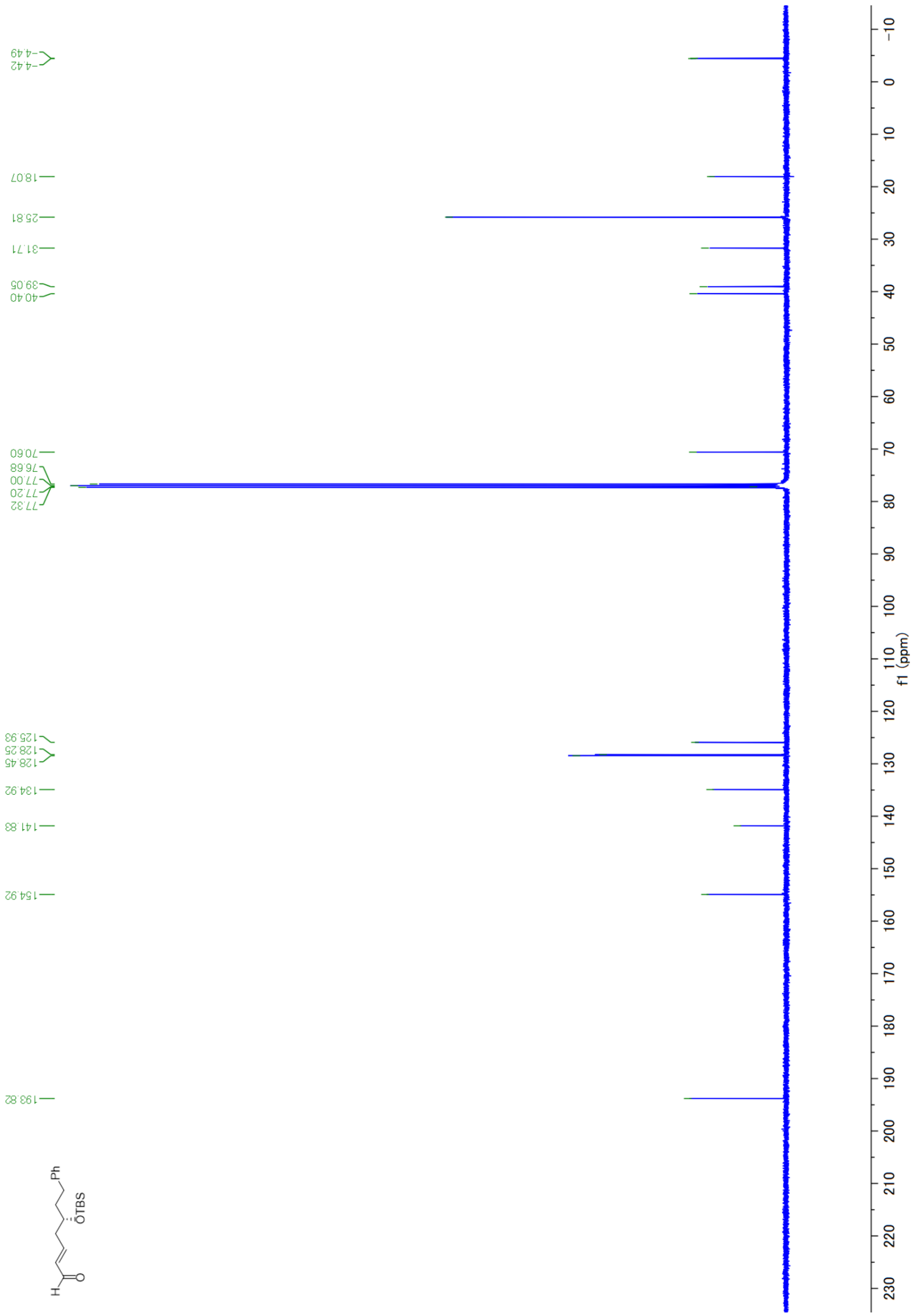
-4.42
-4.49
18.07
25.81
31.71
39.05
40.40
70.60
76.68
77.00
77.20
77.32
125.93
128.25
128.45
134.92
141.83
154.92
193.82
f1 (ppm)
Ph
OTBS
H
O

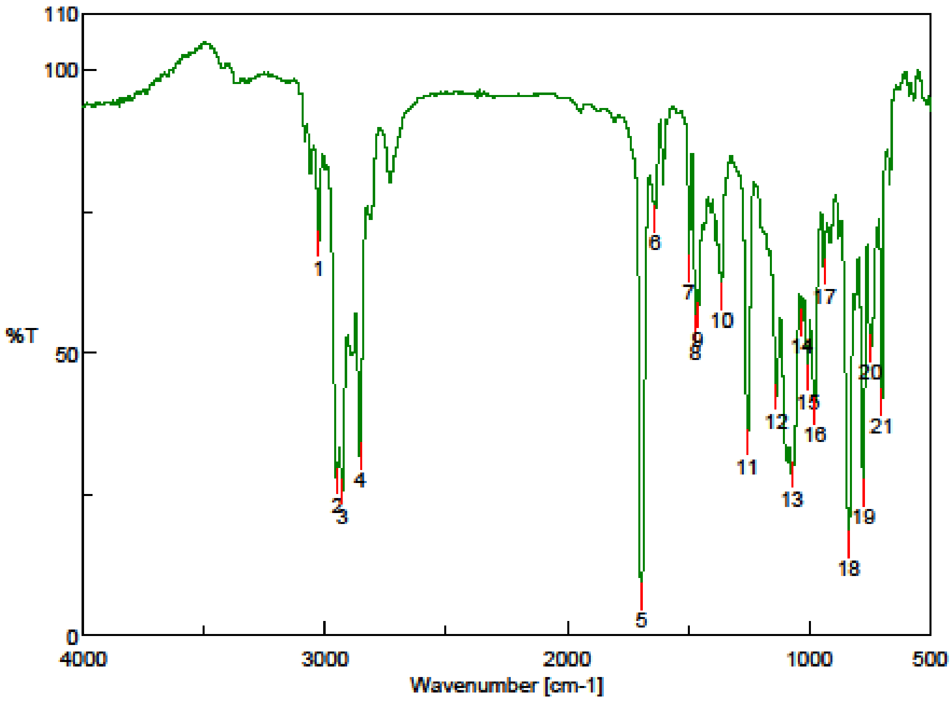

[ピーク検出結果]

No.	位置	強度	No.	位置	強度
1	3027.69	69.1925	2	2952.48	27.1838
3	2929.34	25.3321	4	2857.02	31.5927
5	1694.16	6.96263	6	1638.23	73.6879
7	1496.49	64.9111	8	1472.38	54.1699
9	1461.78	56.6433	10	1361.5	59.8246
11	1256.4	34.1983	12	1136.83	42.1384
13	1072.23	28.4041	14	1030.77	55.3496
15	1006.66	45.4907	16	977.733	39.6103
17	938.199	64.311	18	836.955	16.0342
19	776.208	25.2536	20	747.281	50.7213
21	700.034	41.2974			

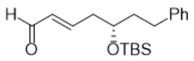

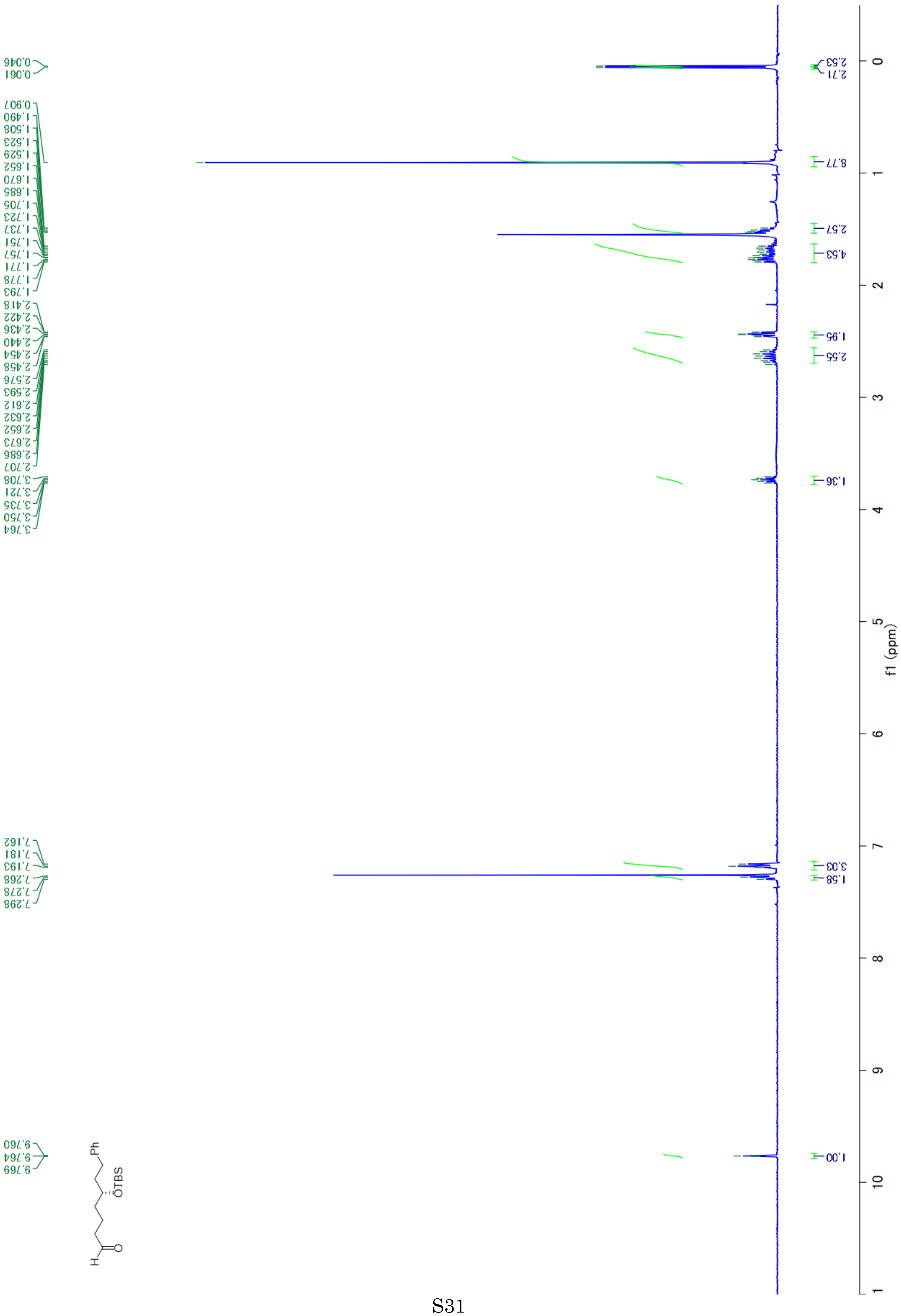

Ph
OTBS
H
O
f1 (ppm)

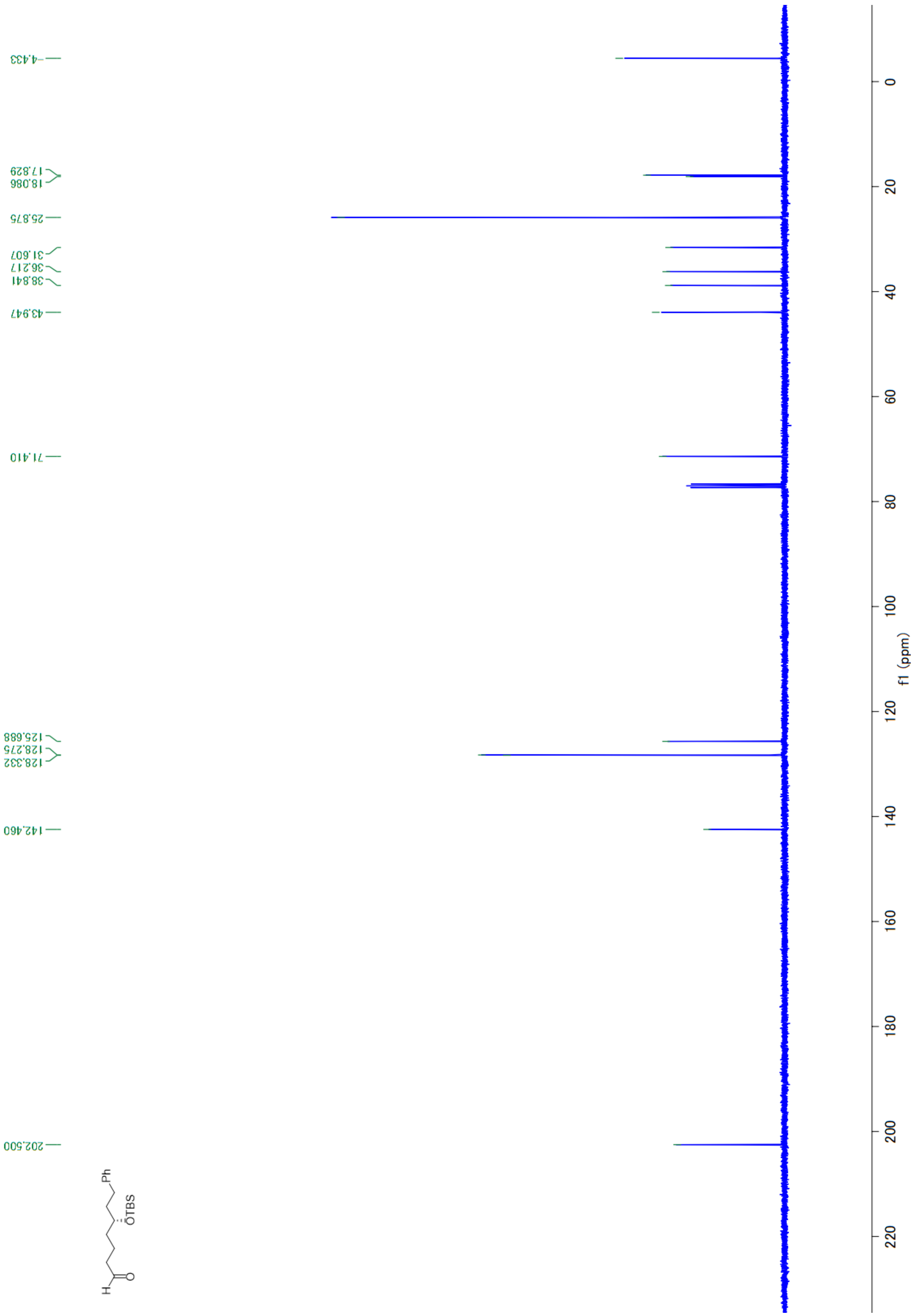

-4.433
17.829
18.086
25.875
31.607
36.217
38.841
43.947
71.410
125.688
128.275
128.332
142.460
202.500
OTBS
Ph
H
O
f1 (ppm)

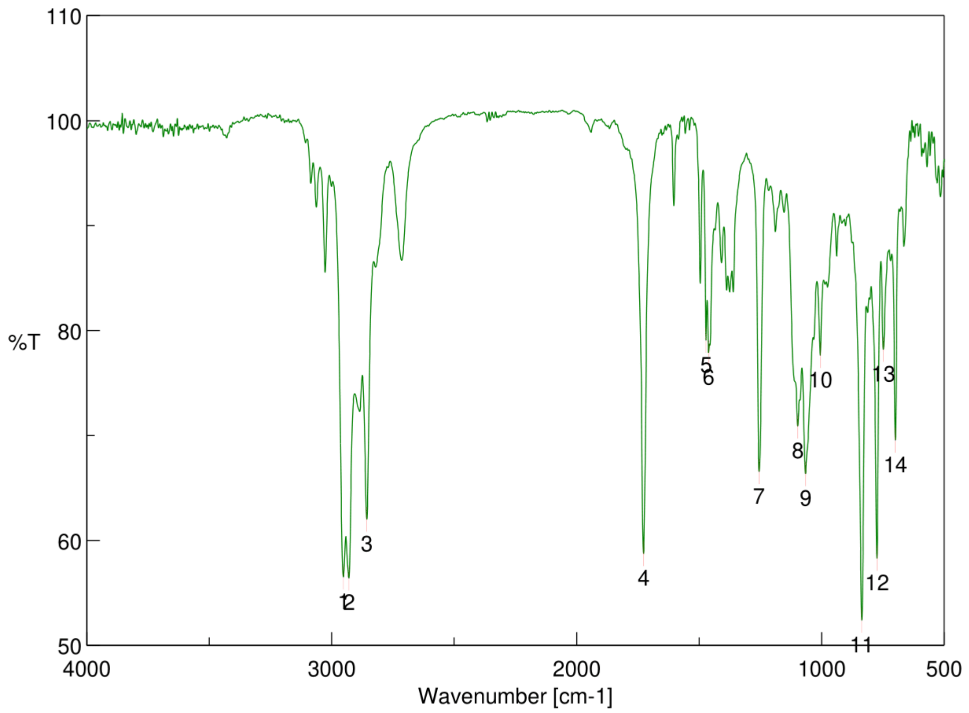

[ピーク検出結果]

No.	位置	強度	No.	位置	強度
1	2952.48	56.5203	2	2929.34	56.429
3	2857.02	62.0003	4	1726.94	58.731
5	1472.38	79.0522	6	1460.81	77.8931
7	1255.43	66.56	8	1097.3	70.8879
9	1065.48	66.3433	10	1005.7	77.6204
11	835.99	52.3991	12	774.279	58.2819
13	748.245	78.1816	14	699.069	69.5435

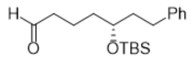

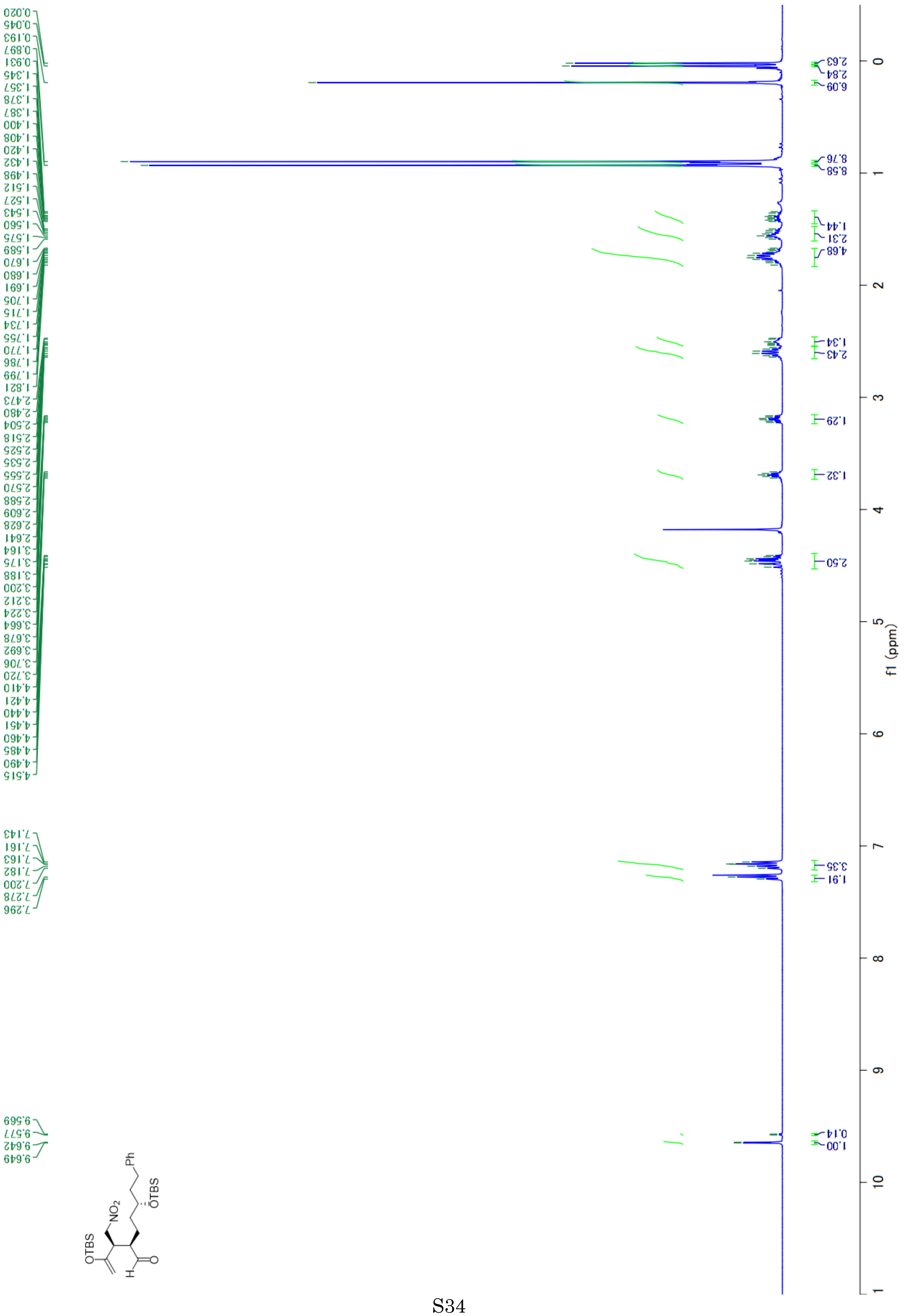

0.020
0.045
0.193
0.897
0.931
1.345
1.357
1.378
1.387
1.400
1.408
1.420
1.432
1.498
1.512
1.527
1.543
1.560
1.575
1.589
1.670
1.680
1.691
1.705
1.715
1.734
1.755
1.770
1.786
1.799
1.821
2.473
2.480
2.504
2.518
2.525
2.535
2.555
2.570
2.588
2.609
2.628
2.641
3.164
3.175
3.188
3.200
3.212
3.224
3.664
3.678
3.692
3.706
3.720
4.410
4.421
4.440
4.451
4.460
4.485
4.490
4.515
7.143
7.161
7.163
7.182
7.200
7.278
7.296
9.569
9.577
9.642
9.649
2.63
2.84
6.09
8.76
8.58
1.44
2.31
4.68
1.34
2.43
1.29
1.32
2.50
3.35
1.91
0.14
1.00
f1 (ppm)
OTBS
NO_2
Ph
OTBS
H
O

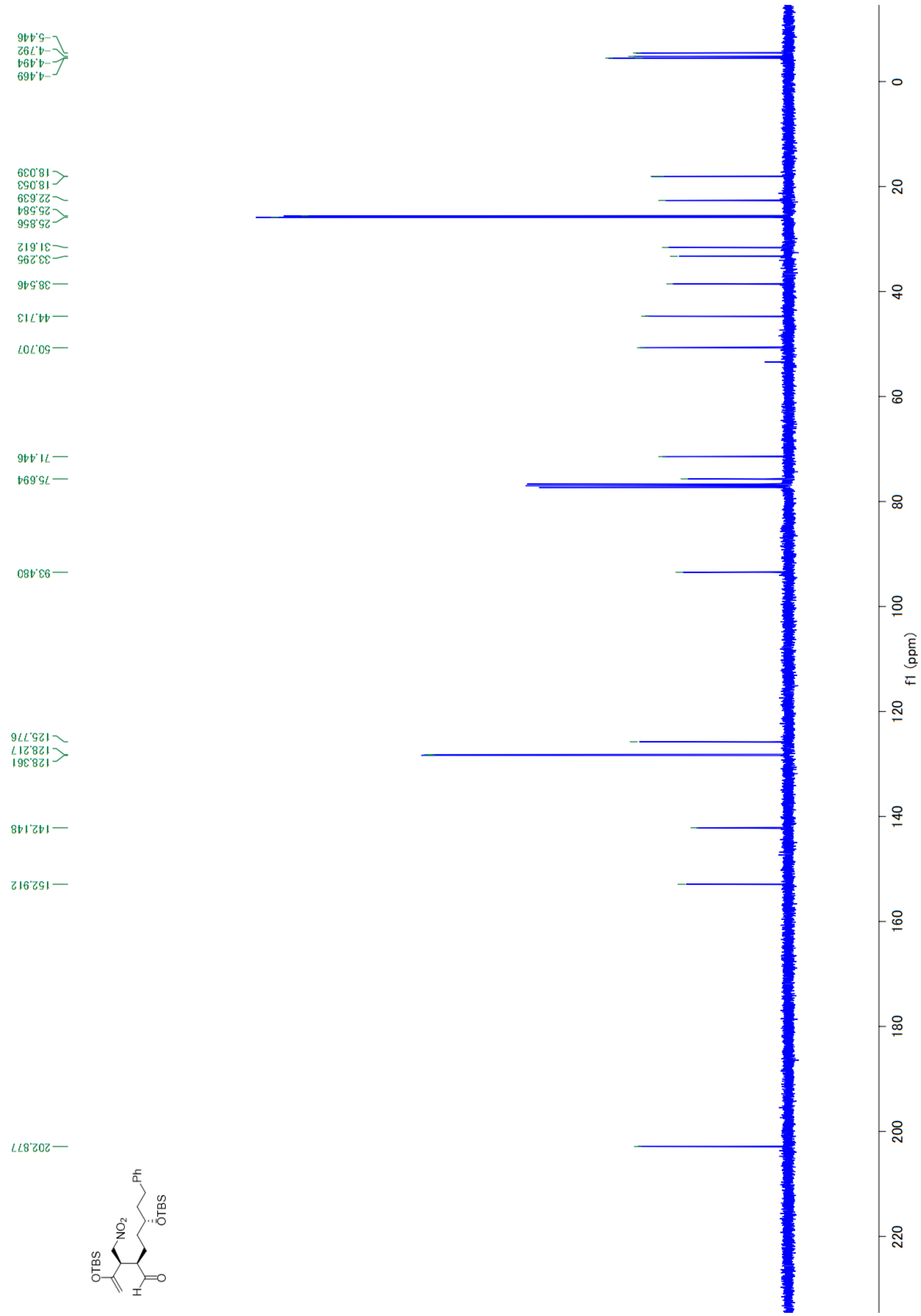

-5.446
-4.792
-4.494
-4.469
18.039
18.053
22.639
25.584
25.856
31.612
33.295
38.546
44.713
50.707
71.446
75.694
93.480
125.776
128.217
128.361
142.148
152.912
202.877
f1 (ppm)
OTBS
NO2
Ph
OTBS
H
O

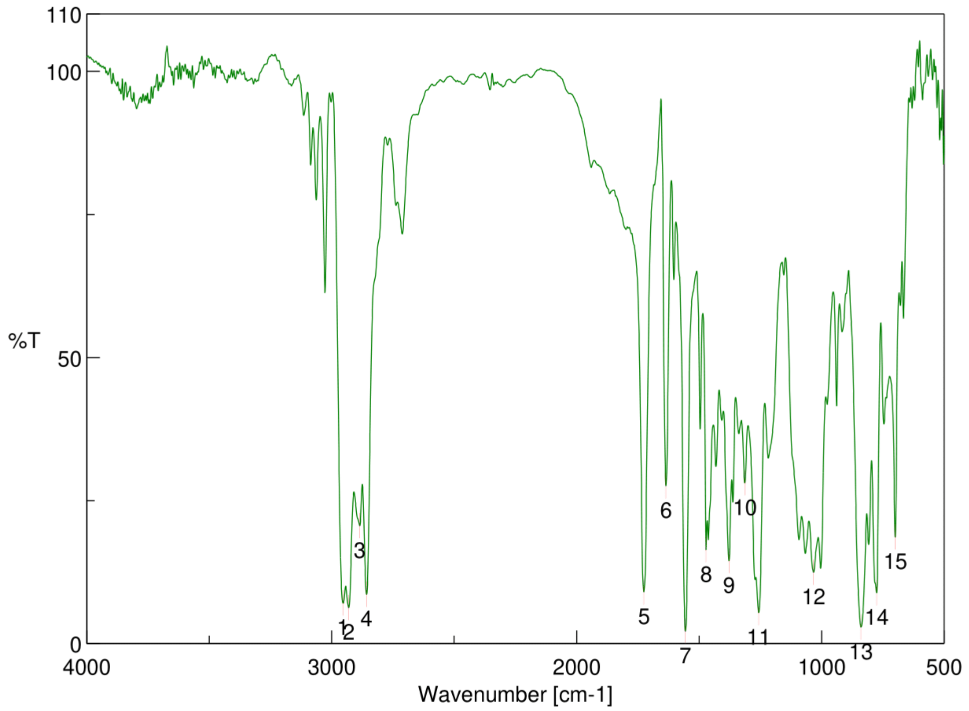

[ピーク検出結果]

No.	位置	強度	No.	位置	強度
1	2954.41	7.08602	2	2930.31	6.2795
3	2885.95	20.6093	4	2857.99	8.60048
5	1725.98	9.0135	6	1635.34	27.5647
7	1556.27	2.16618	8	1471.42	16.387
9	1377.89	14.4652	10	1313.29	28.0542
11	1256.4	5.39997	12	1032.69	12.4636
13	838.883	2.90289	14	775.244	8.86751
15	699.069	18.6111			

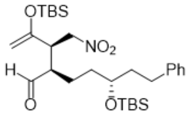

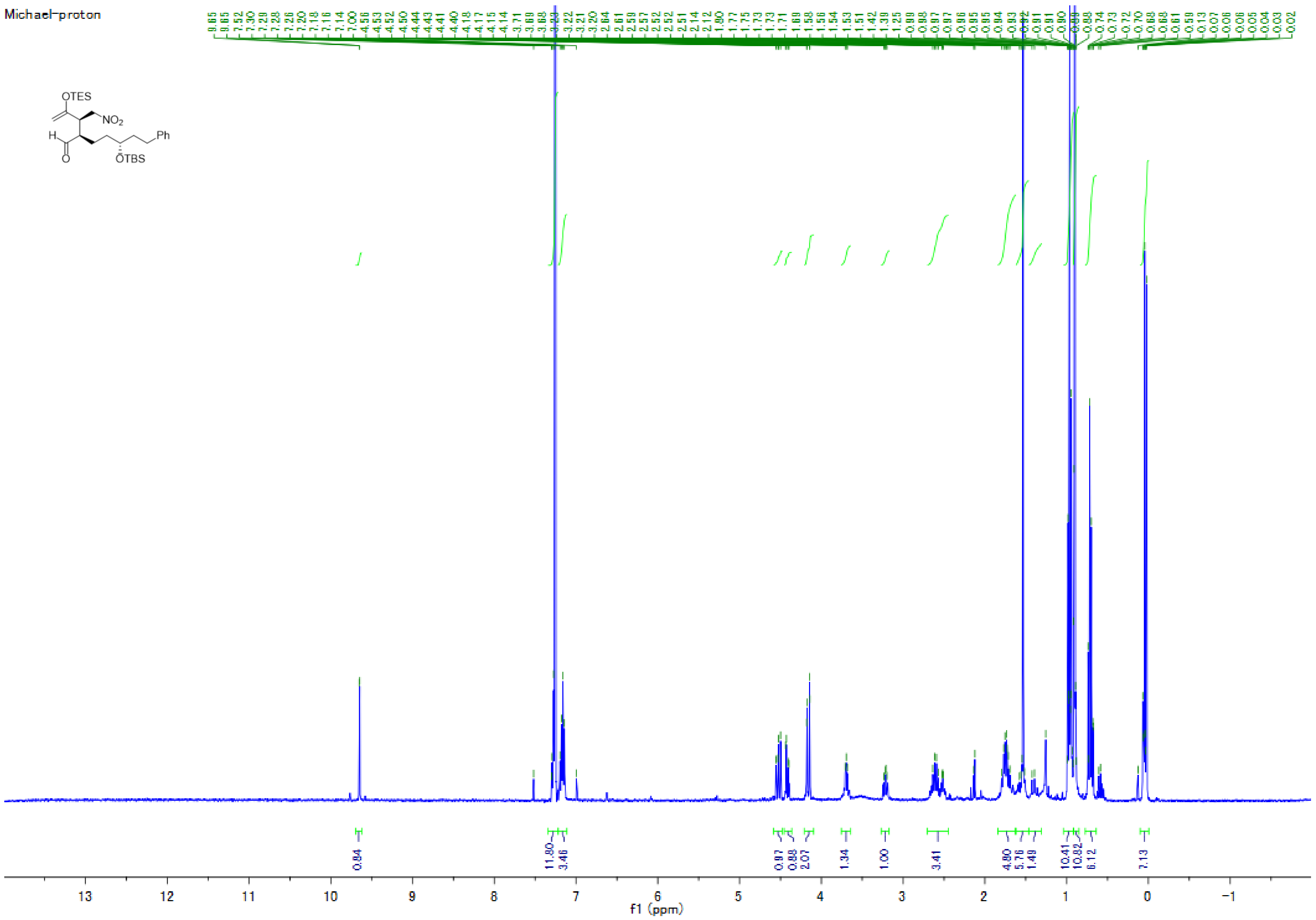
Michael-proton
OTES
NO2
H
O
OTBS
Ph
0.84
11.80
3.46
0.97
0.88
2.07
1.34
1.00
3.41
4.80
5.76
1.49
10.41
10.82
6.12
7.13
f1 (ppm)

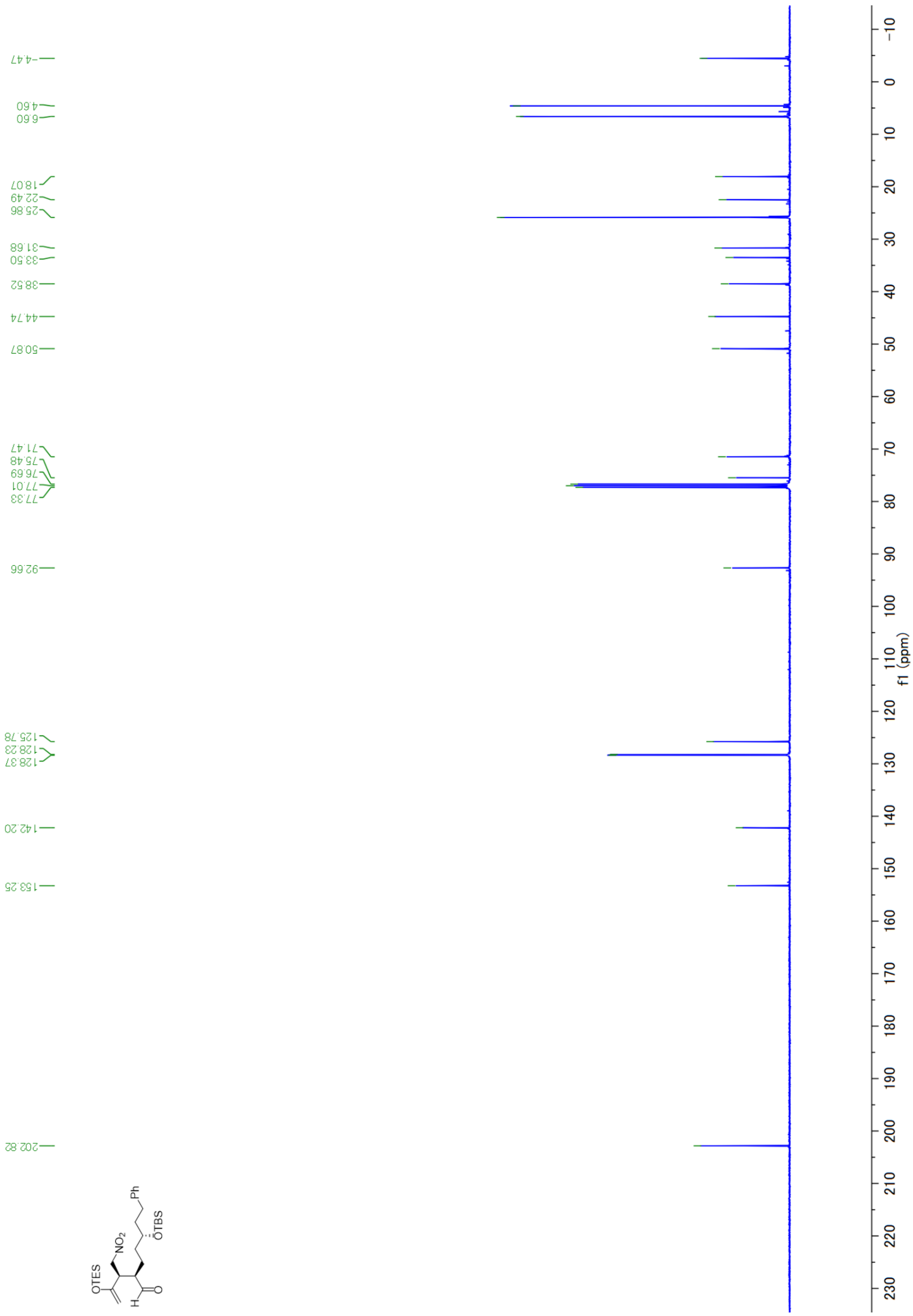

-4.47
4.60
6.60
18.07
22.49
25.86
31.68
33.50
38.52
44.74
50.87
71.47
75.48
76.69
77.01
77.33
92.66
125.78
128.23
128.37
142.20
153.25
202.82
f1 (ppm)
OTES
NO2
Ph
OTBS
H
O

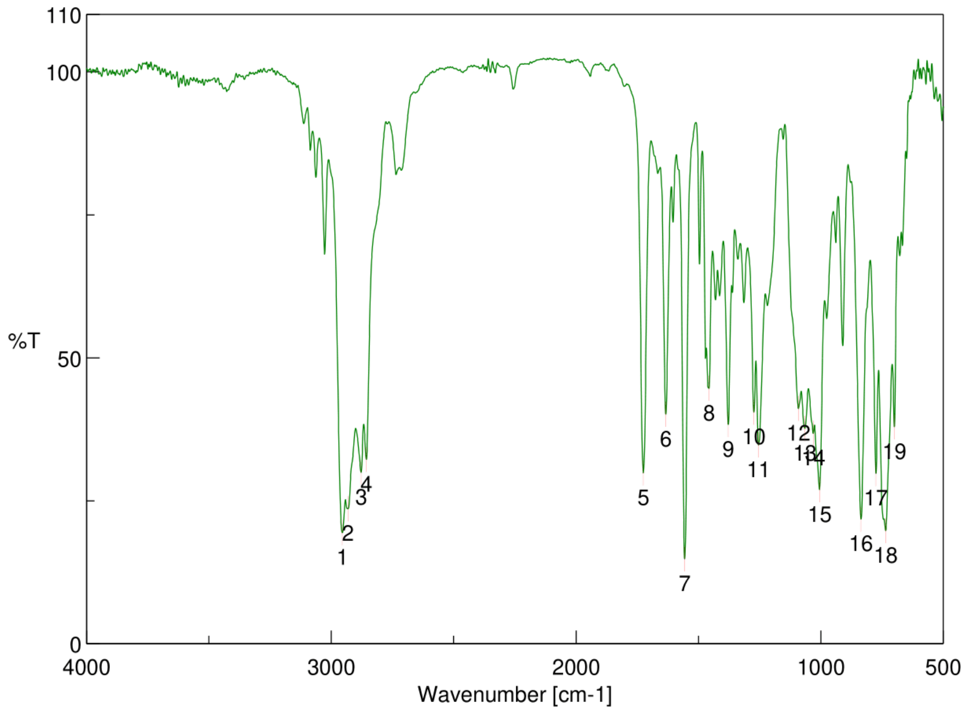

[ピーク検出結果]

No.	位置	強度	No.	位置	強度
1	2955.38	19.3965	2	2931.27	23.5927
3	2878.24	29.9575	4	2857.02	32.2007
5	1725.01	29.843	6	1633.41	40.0903
7	1556.27	14.8317	8	1456.96	44.6393
9	1378.85	38.3373	10	1273.75	40.4666
11	1254.47	34.7418	12	1091.51	41.1178
13	1065.48	37.6384	14	1030.77	36.7461
15	1005.7	26.907	16	835.99	21.7833
17	775.244	29.7326	18	735.71	19.773
19	700.034	37.8804			

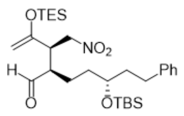

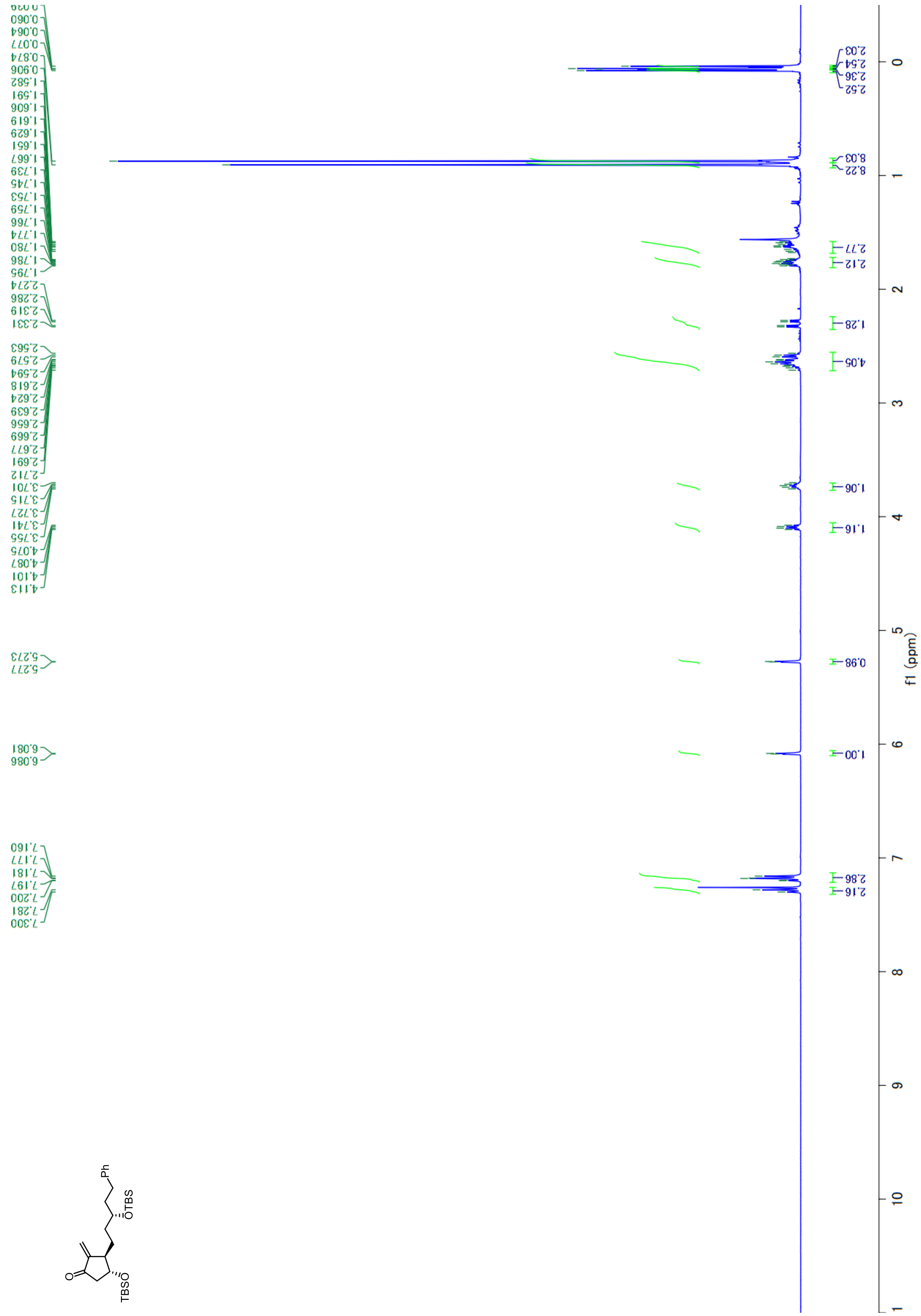
0.049
0.060
0.064
0.077
0.874
0.906
1.582
1.591
1.606
1.619
1.629
1.651
1.667
1.739
1.745
1.753
1.759
1.766
1.774
1.780
1.786
1.795
2.274
2.286
2.319
2.331
2.563
2.579
2.594
2.618
2.624
2.639
2.656
2.669
2.677
2.691
2.712
3.701
3.715
3.727
3.741
3.755
4.075
4.087
4.101
4.113
5.273
5.277
6.081
6.086
7.160
7.177
7.181
7.197
7.200
7.281
7.300
2.03
2.54
2.36
2.52
8.03
8.22
2.77
2.12
1.28
4.05
1.06
1.16
0.98
1.00
2.86
2.16
f1 (ppm)
O
TBSO
OTBS
Ph

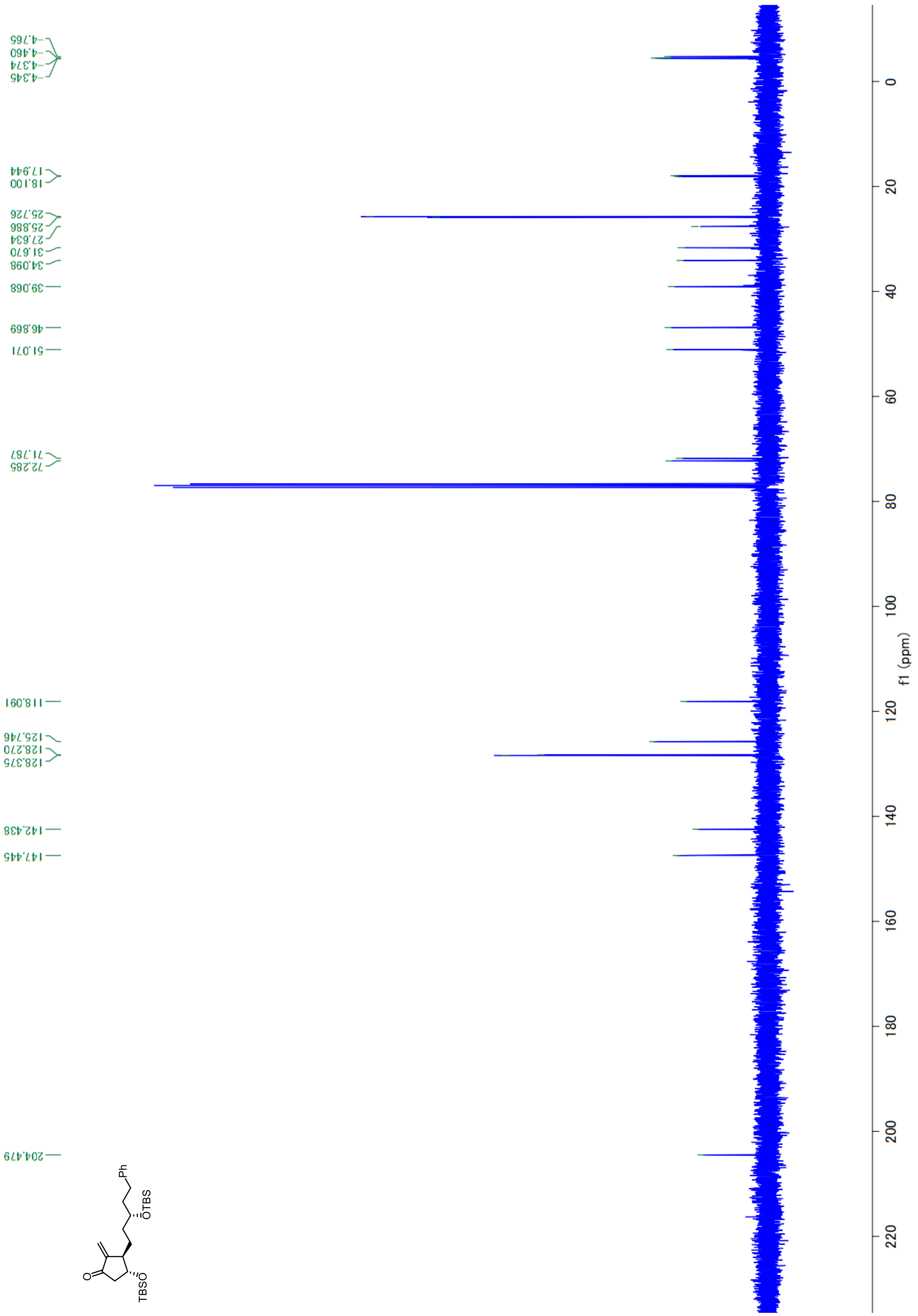

-4.765
-4.460
-4.374
-4.345
17.944
18.100
25.726
25.886
27.634
31.670
34.098
39.068
46.869
51.071
71.787
72.285
118.091
125.746
128.270
128.375
142.438
147.445
204.479
f1 (ppm)
Ph
OTBS
TBSO
O

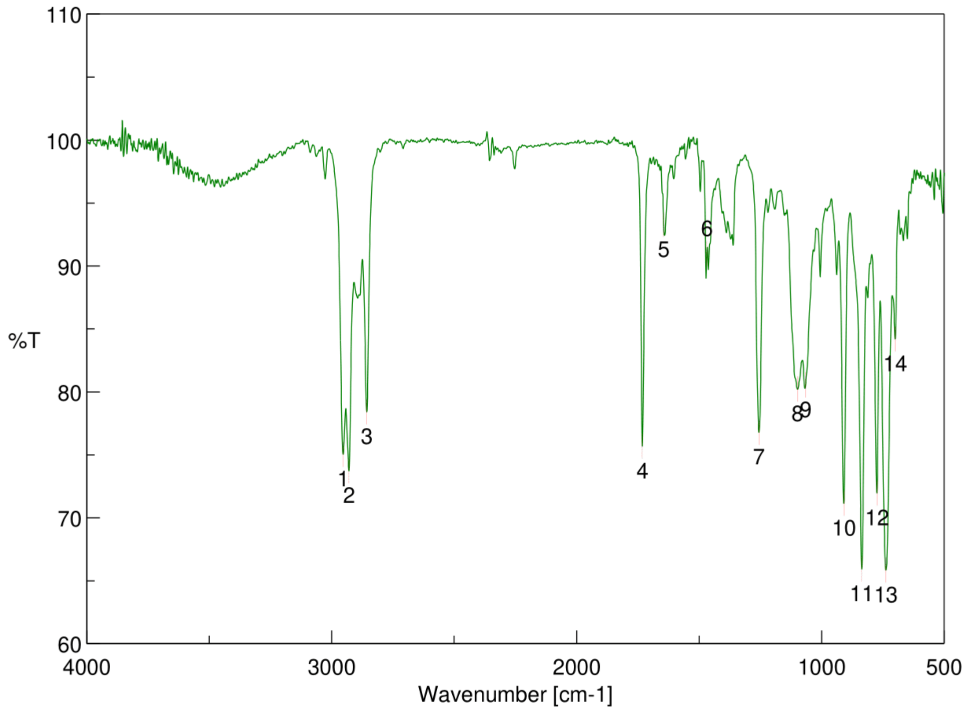

[ピーク検出結果]

No.	位置	強度	No.	位置	強度
1	2953.45	75.0153	2	2929.34	73.717
3	2857.02	78.4041	4	1731.76	75.6663
5	1644.98	93.2402	6	1468.53	90.9814
7	1255.43	76.7797	8	1098.26	80.2095
9	1065.48	80.5316	10	909.272	71.1243
11	835.99	65.908	12	774.279	71.94
13	737.639	65.8254	14	700.034	84.184

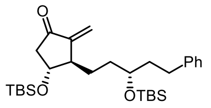

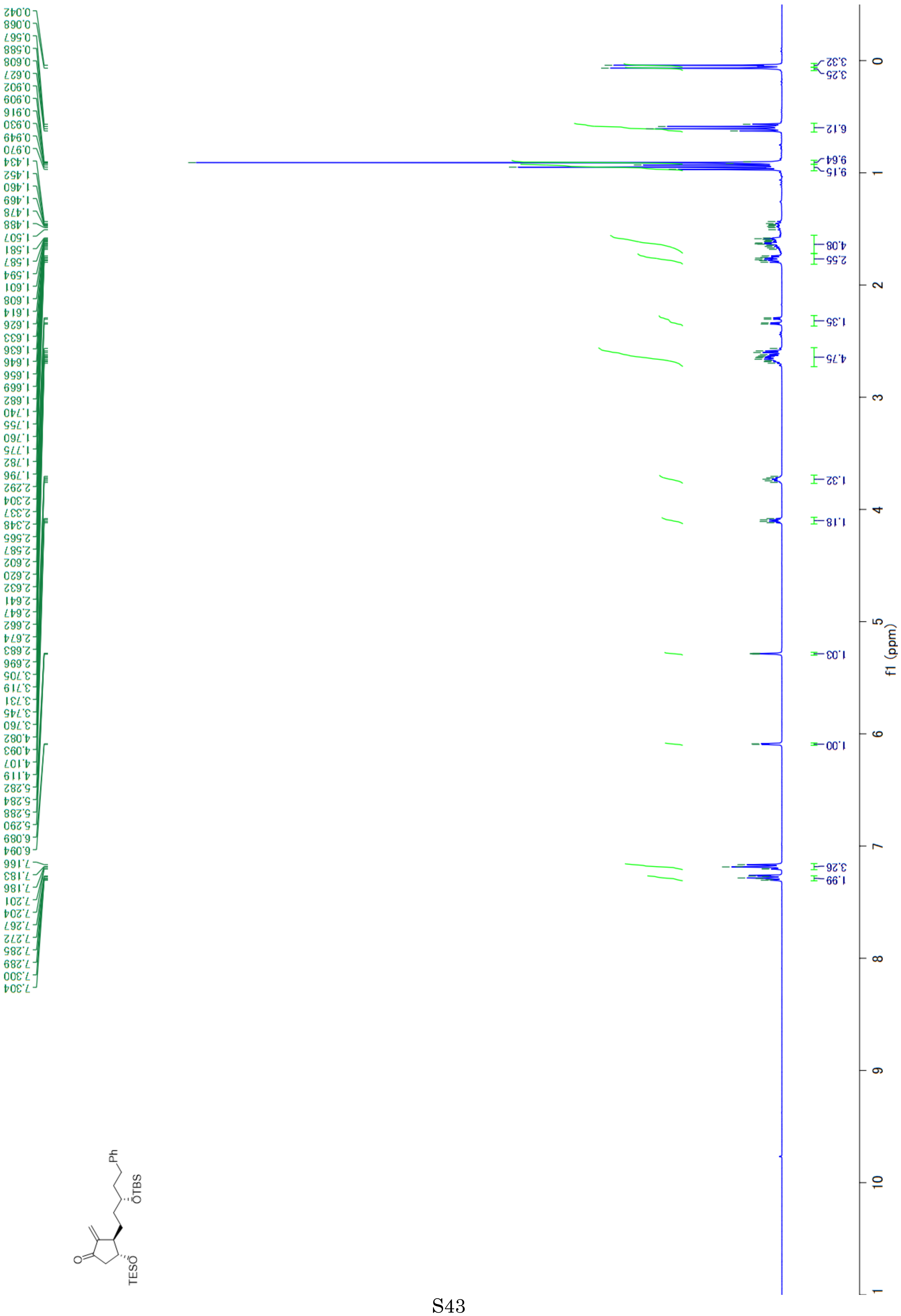
f1 (ppm)
TESO
OTBS
Ph

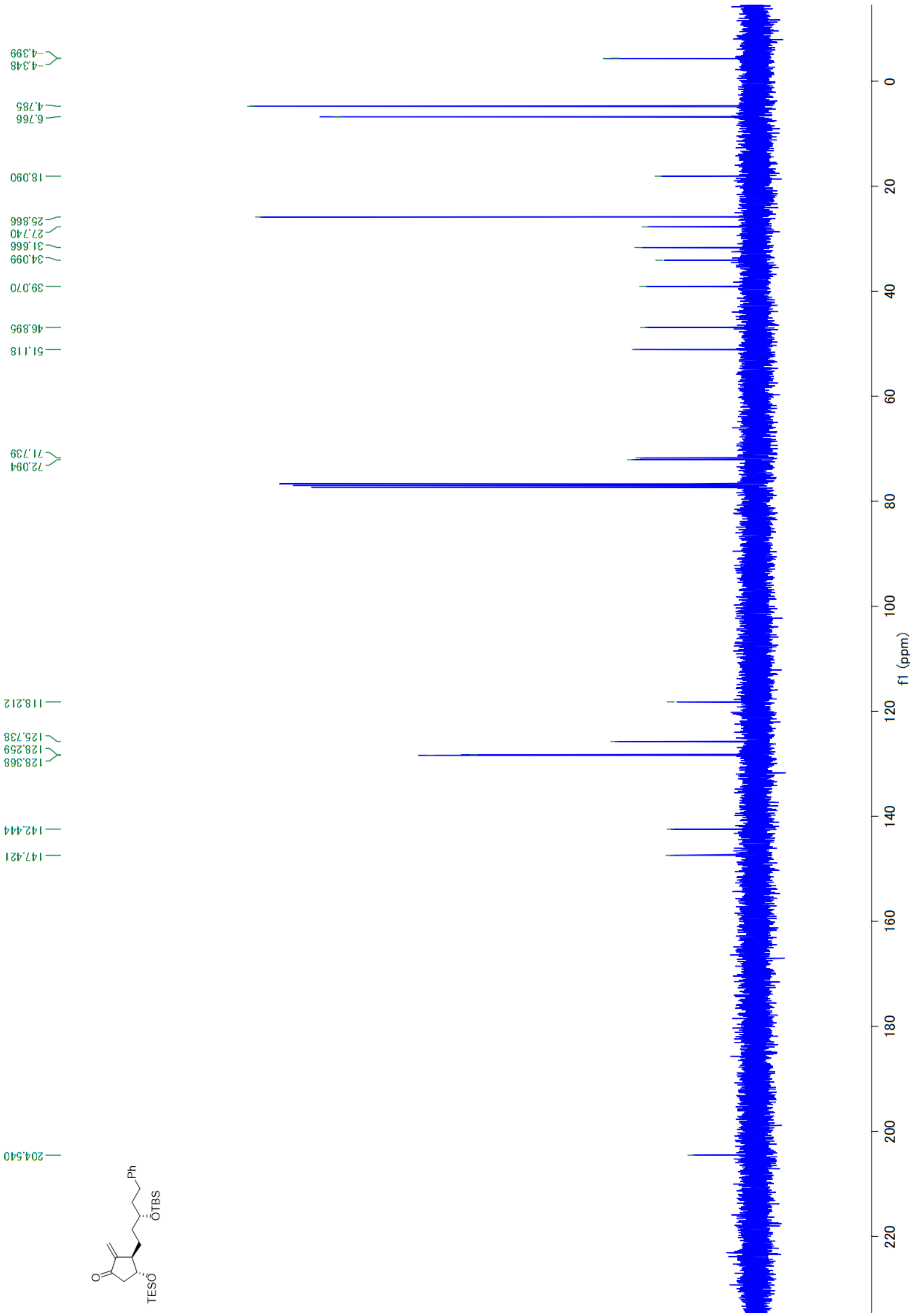
-4.399
-4.348
4.785
6.766
18.090
25.866
27.740
31.666
34.099
39.070
46.895
51.118
71.739
72.094
118.212
125.738
128.259
128.368
142.444
147.421
204.540
f1 (ppm)
O
TESO
OTBS
Ph

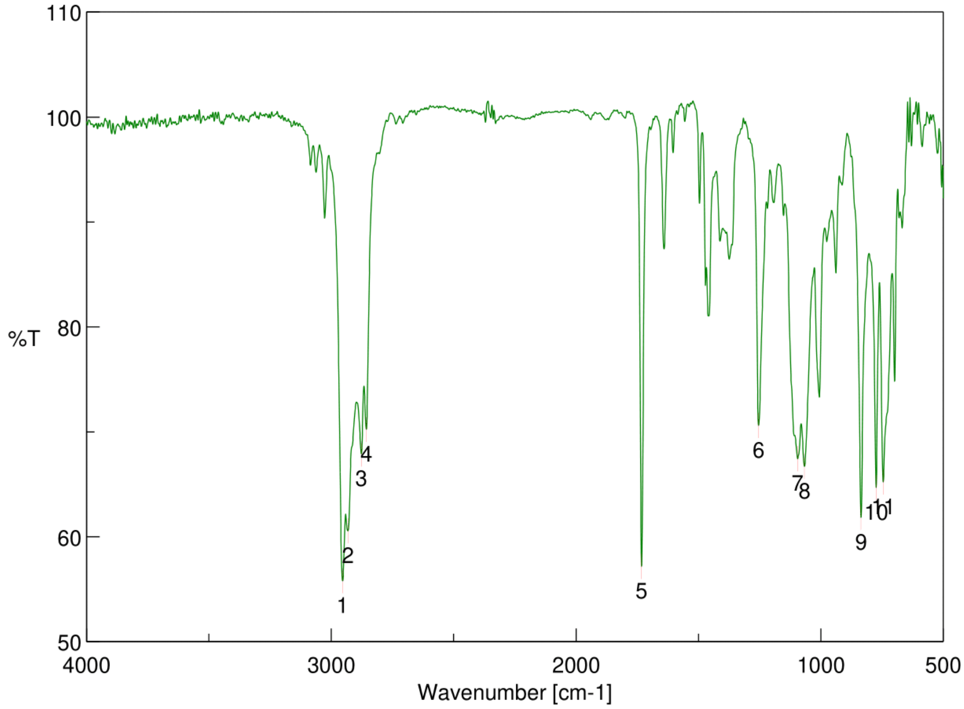

[ピーク検出結果]

No.	位置	強度	No.	位置	強度
1	2954.41	55.7936	2	2932.23	60.5617
3	2877.27	67.8722	4	2857.02	70.224
5	1732.73	57.1651	6	1254.47	70.5878
7	1095.37	67.4285	8	1067.41	66.6962
9	835.99	61.8414	10	774.279	64.6805
11	745.352	65.2037			

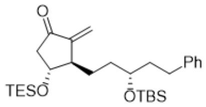

ﾍﾟｰｼﾞ 1/1

面積%レポート

ﾃﾞｰﾀﾌｧｲﾙ名 : C:¥Documents and Settings¥東京理科大学¥デスクトップ¥HPLCデータ¥kawauchi¥2023-07-26 17-04-27 kawauchi Mukaiyamaaldol OD-H 2000vs1 1mL chi.dat
ﾒｿｯﾄﾞﾌｧｲﾙ名: C:¥EZChrom Elite¥Enterprise¥Projects¥Default¥Method¥100vs1.met
ﾕｰｻﾞｰ名: System
分析日時: 2023/07/26 17:05:04
印刷日時: 2023/07/26 17:57:32

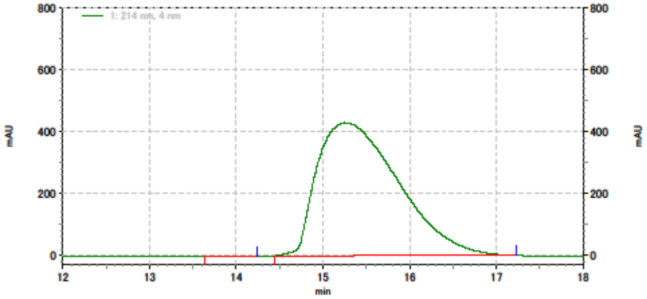

1: 214 nm, 4 nm結果

Pk #	名前	保持時間	面積	面積%	ﾍﾞｰｽﾗｲﾝｺｰﾄﾞ
1		13.97	75482	0.067	MM
2		15.25	112229977	99.933	MM
ﾄｰﾀﾙ			112305459	100.000	

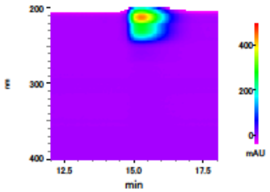

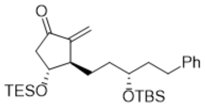

ﾍﾟｰｼﾞ 1/1

面積%レポート

ﾃﾞｰﾀﾌｧｲﾙ名 : C:¥Documents and Settings¥東京理科大学¥デスクトップ¥HPLCデータ¥kawauchi¥2023-07-26 17-58-30 kawauchi Mukaiyamaaldol OD-H 2000vs1 1mL rac.dat
ﾒｿｯﾄﾞﾌｧｲﾙ名: C:¥EZChrom Elite¥Enterprise¥Projects¥Default¥Method¥100vs1.met
ﾕｰｻﾞｰ名: System
分析日時: 2023/07/26 17:59:08
印刷日時: 2023/07/26 18:21:43

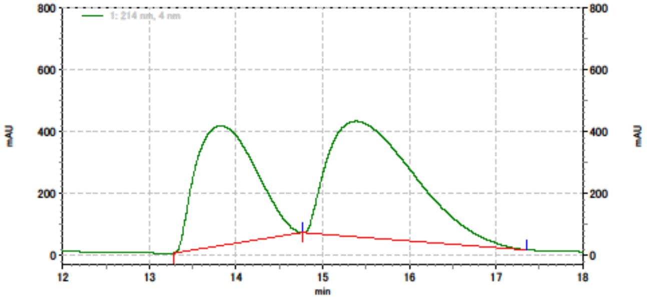

1: 214 nm, 4 nm結果

Pk #	名前	保持時間	面積	面積%	ﾍﾞｰｽﾗｲﾝｺｰﾄﾞ
1		13.83	74560763	41.595	MM
2		15.38	104692772	58.405	MM
ﾄｰﾀﾙ			179253535	100.000	

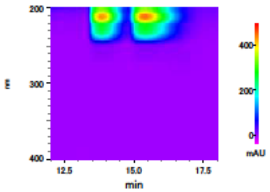

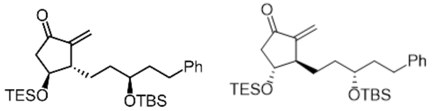

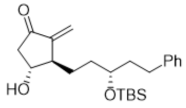
O
HO
OTBS
Ph

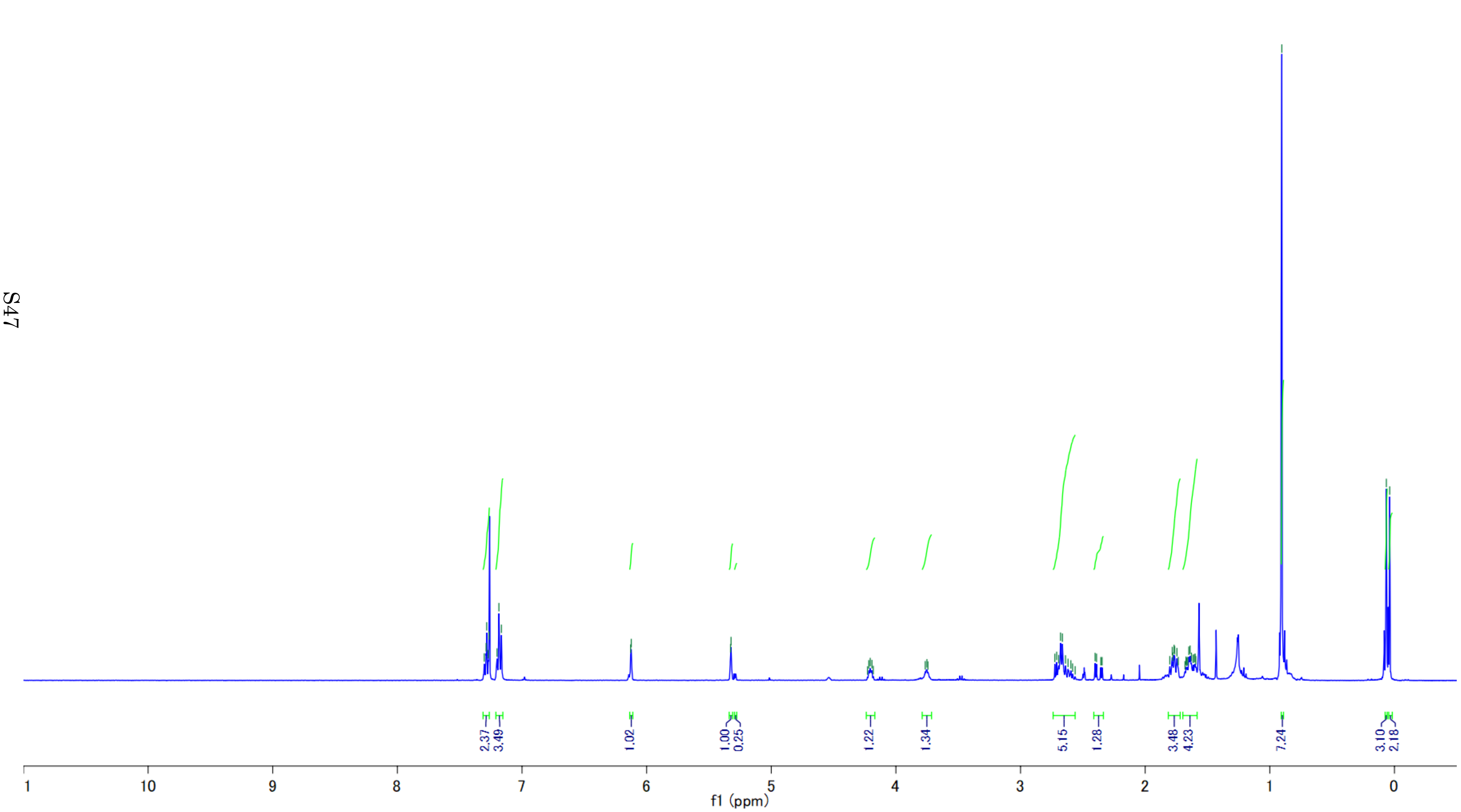
7.303
7.288
7.283
7.271
7.266
7.200
7.185
7.165
6.128
6.123
5.327
5.323
4.228
4.217
4.206
4.192
4.182
3.765
3.751
3.743
2.725
2.710
2.694
2.679
2.664
2.639
2.619
2.596
2.582
2.562
2.402
2.390
2.356
2.346
1.802
1.783
1.778
1.768
1.763
1.745
1.735
1.680
1.672
1.667
1.658
1.650
1.640
1.629
1.615
1.605
1.599
1.594
0.904
0.065
0.038
2.37
3.49
1.02
1.00
0.25
1.22
1.34
5.15
1.28
3.48
4.23
7.24
3.10
2.18
1
10
9
8
7
6
5
4
3
2
1
0
f1 (ppm)

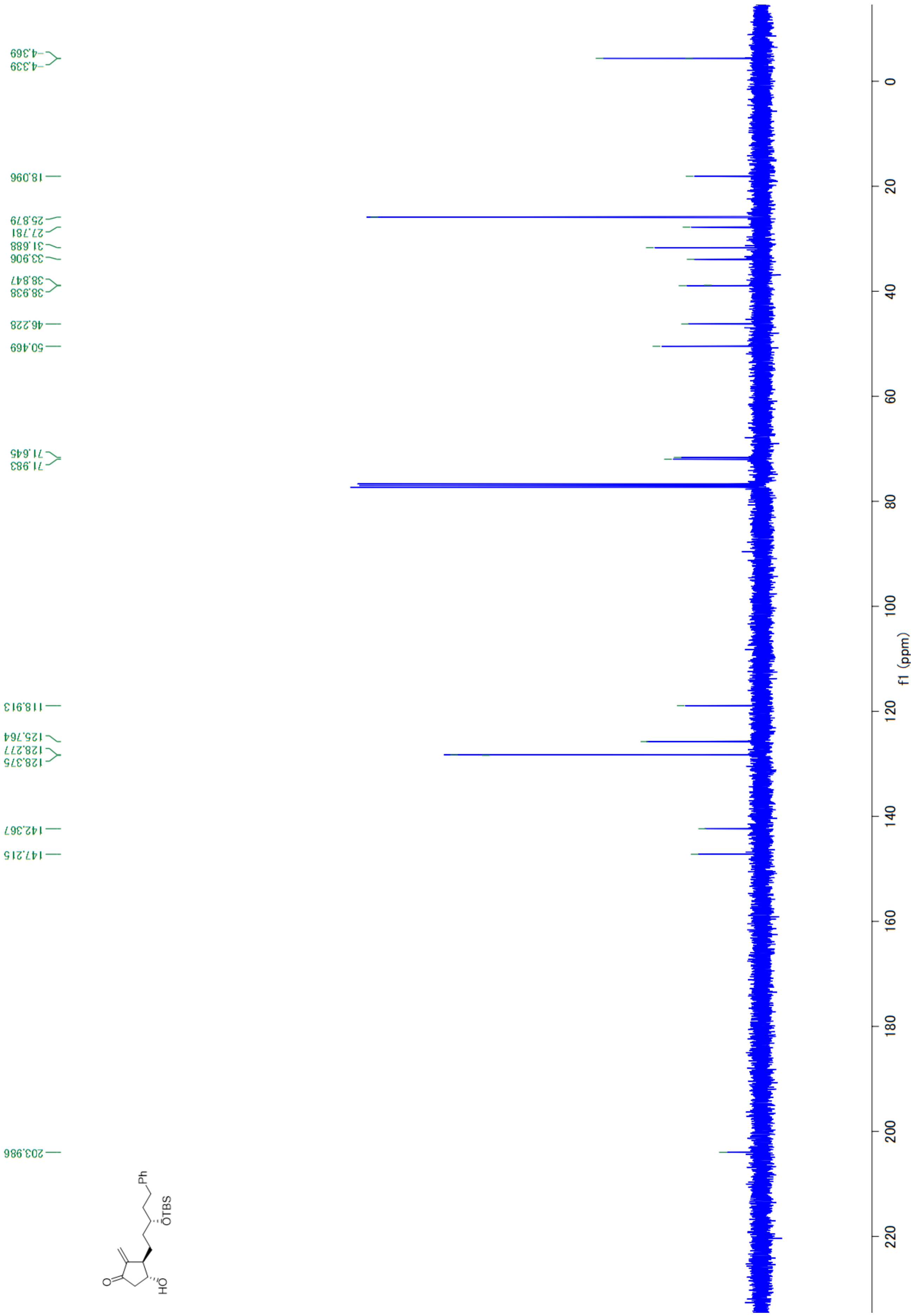

-4.369
-4.339
18.096
25.879
27.781
31.688
33.906
38.847
38.938
46.228
50.469
71.645
71.983
118.913
125.764
128.277
128.375
142.367
147.215
203.986
f1 (ppm)
OTBS
Ph
HO
O

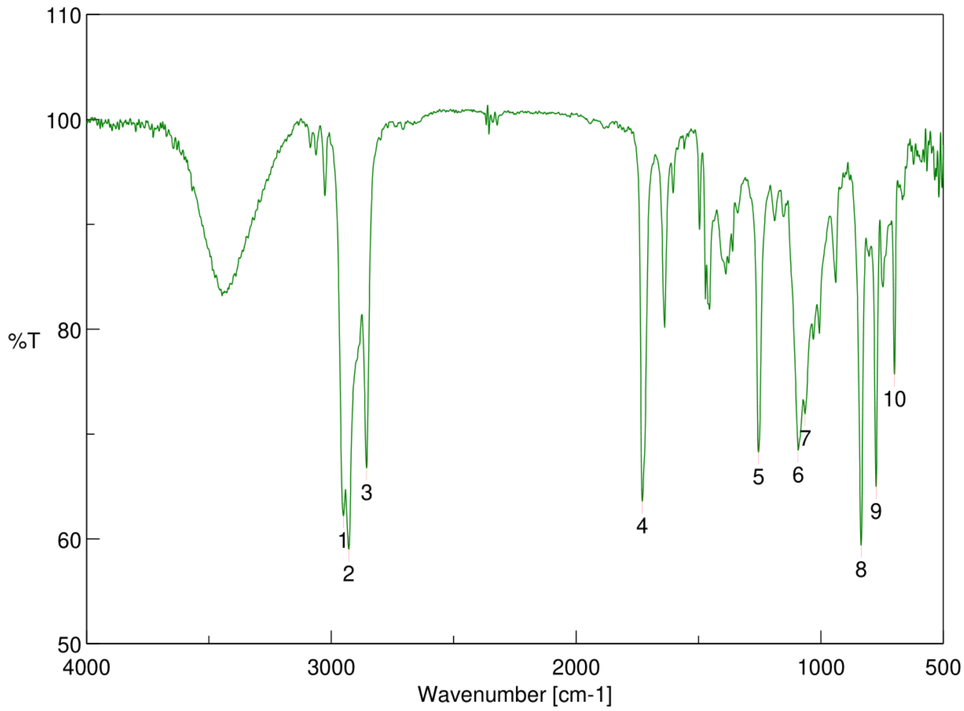

[ピーク検出結果]

No.	位置	強度	No.	位置	強度
1	2950.55	62.1975	2	2928.38	59.013
3	2856.06	66.7459	4	1729.83	63.5885
5	1254.47	68.2806	6	1092.48	68.4626
7	1064.51	71.9217	8	835.026	59.4085
9	774.279	64.9788	10	699.069	75.6887

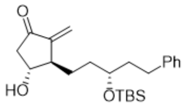

HPLC chart of compound **17** after deprotection of **3**

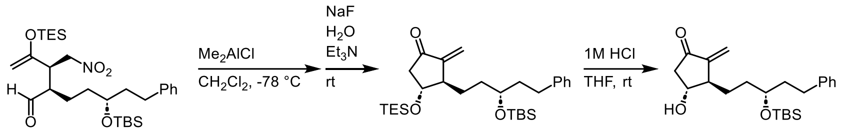

5 **3** 74% **17** 54%

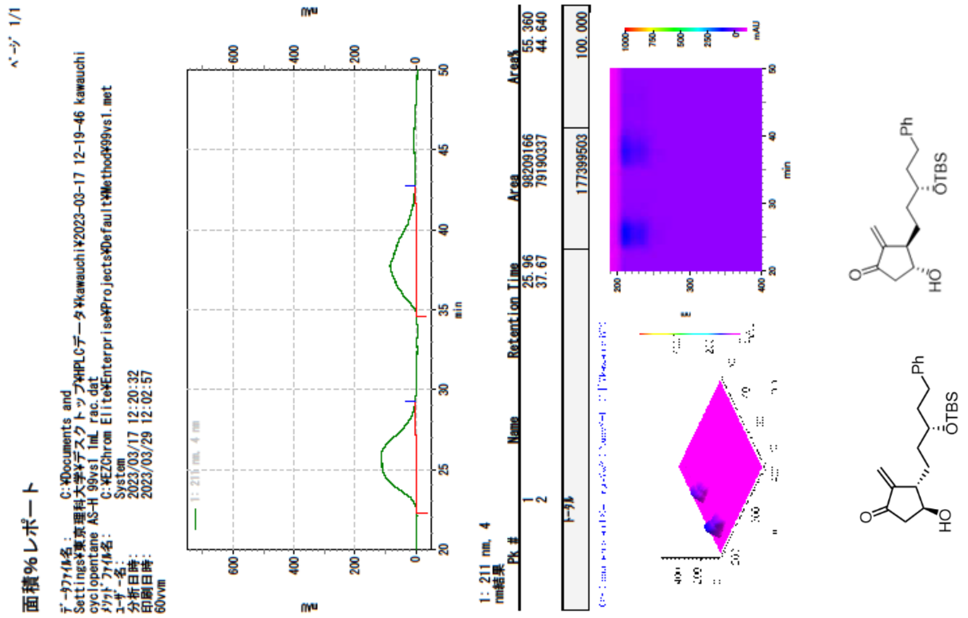

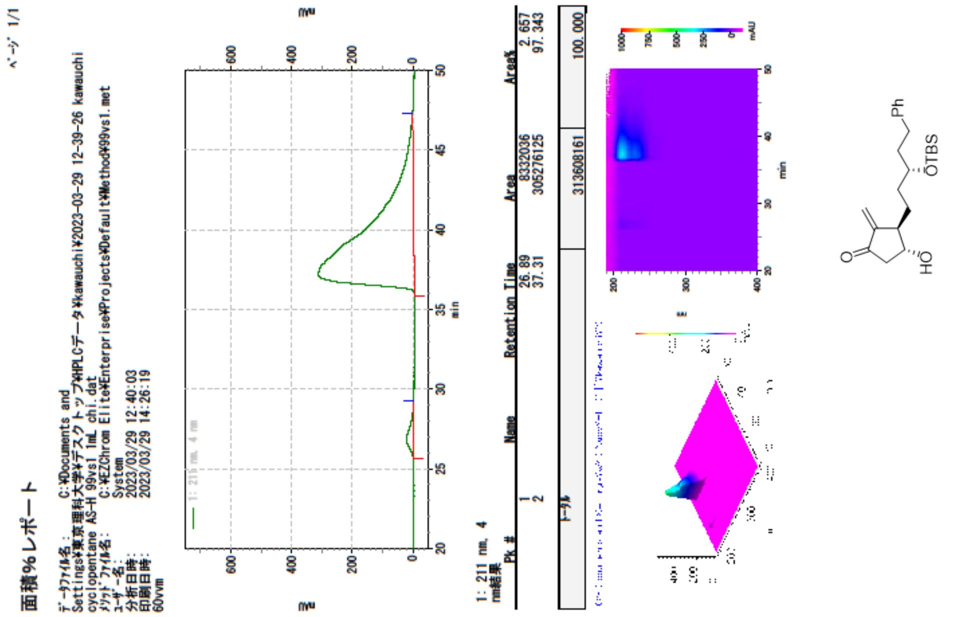

HPLC chart of **17** after Mukaiyama aldol reaction

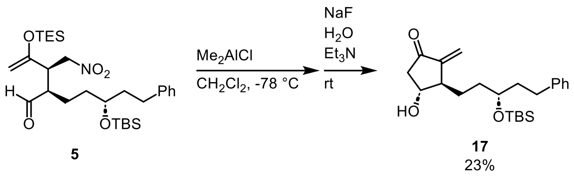

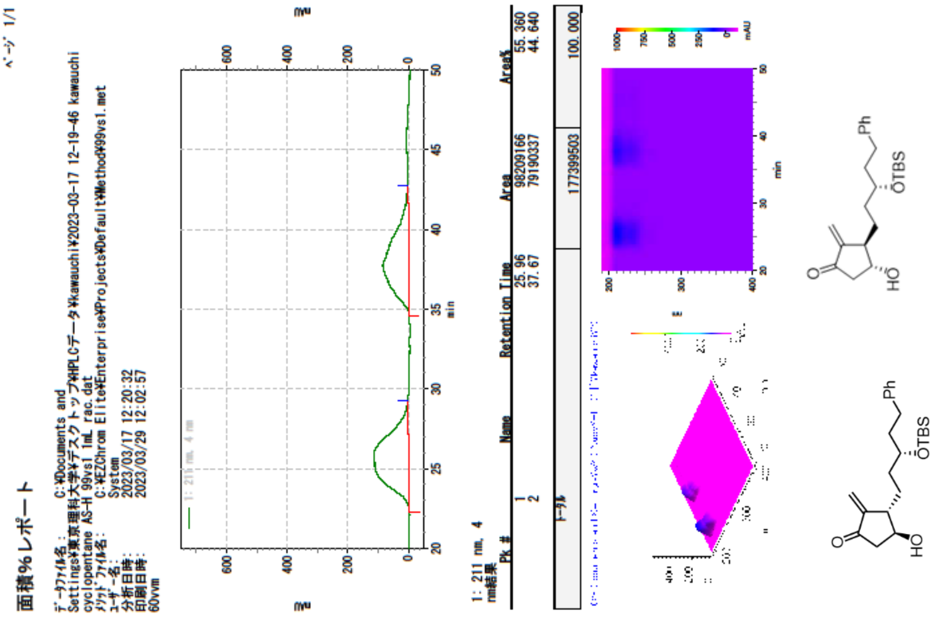

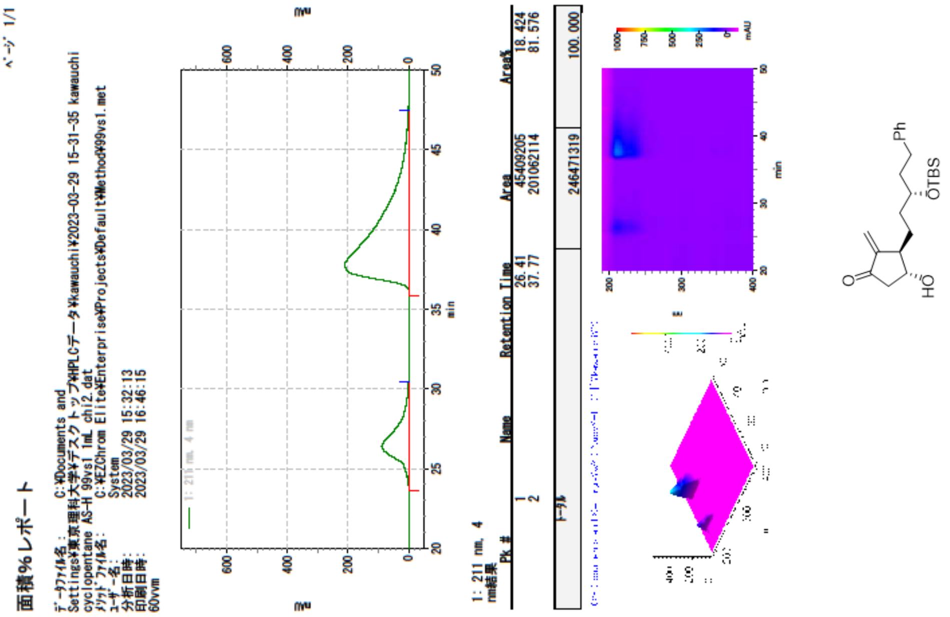

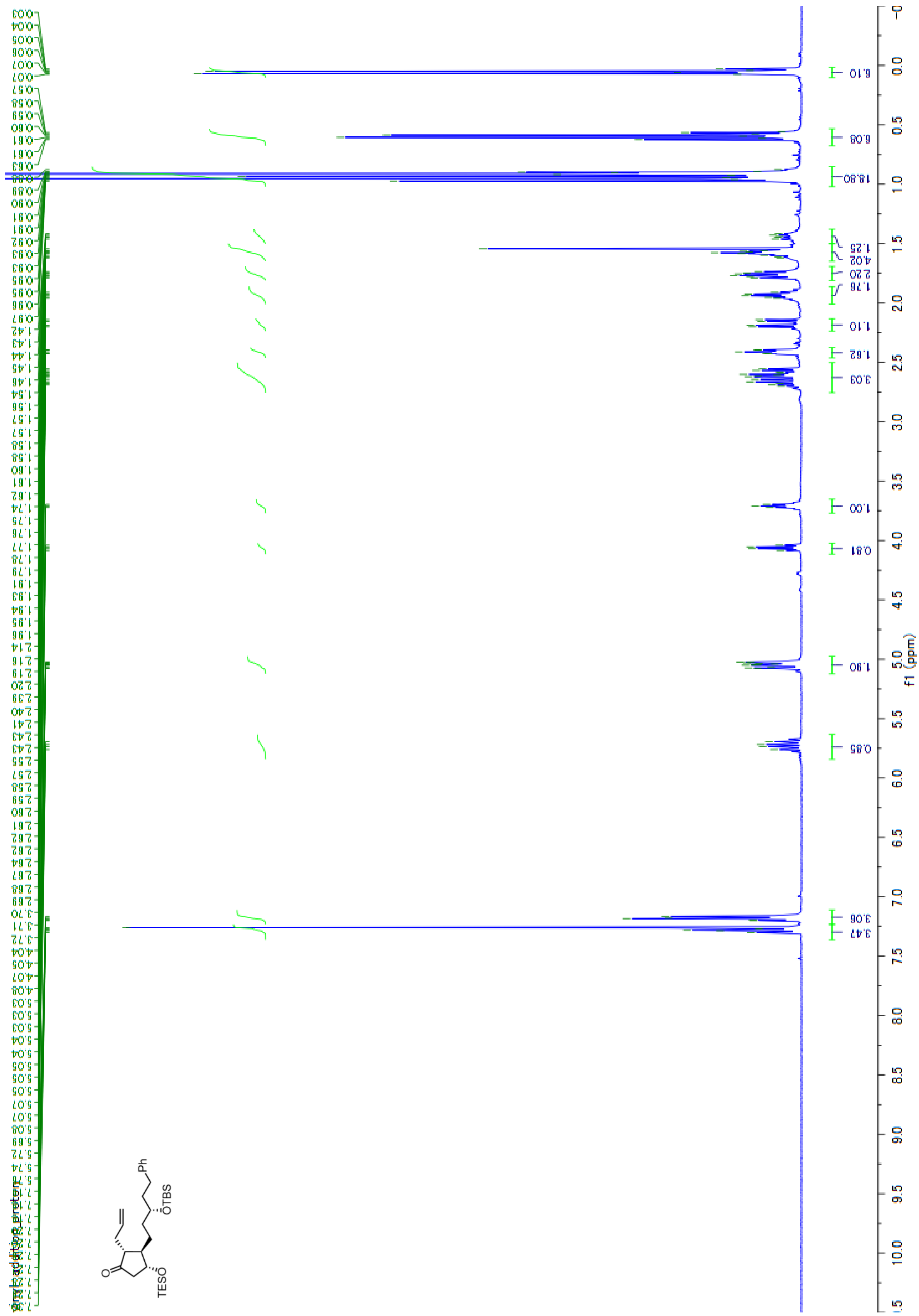
f1 (ppm)

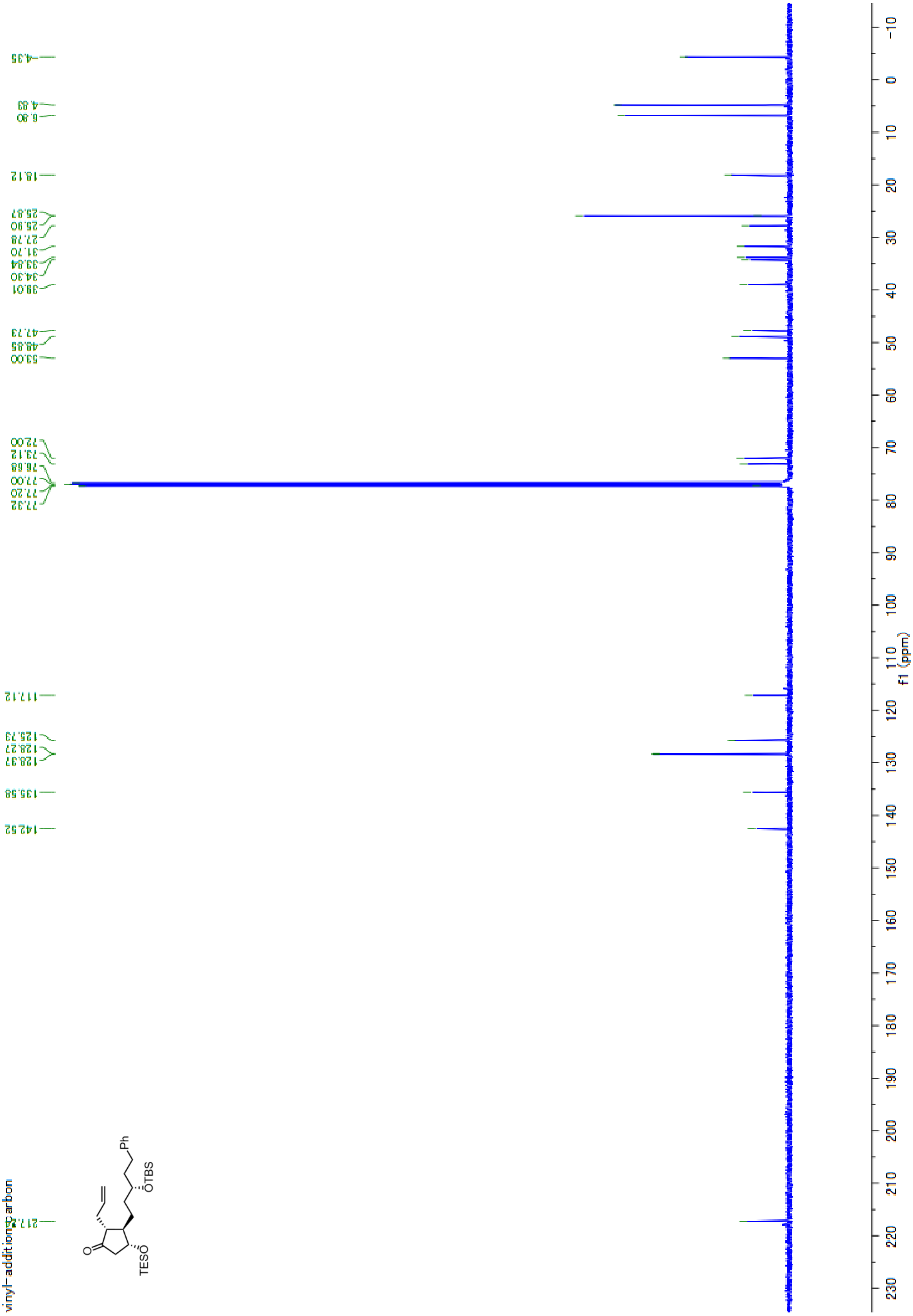
vinyl-addition carbon
TESO
OTBS
Ph
f1 (ppm)
-4.35
4.83
6.80
18.12
25.87
25.90
27.78
31.70
33.84
34.30
39.01
47.73
48.95
53.00
72.00
73.12
76.68
77.00
77.20
77.32
117.12
125.73
128.27
128.37
135.58
142.52

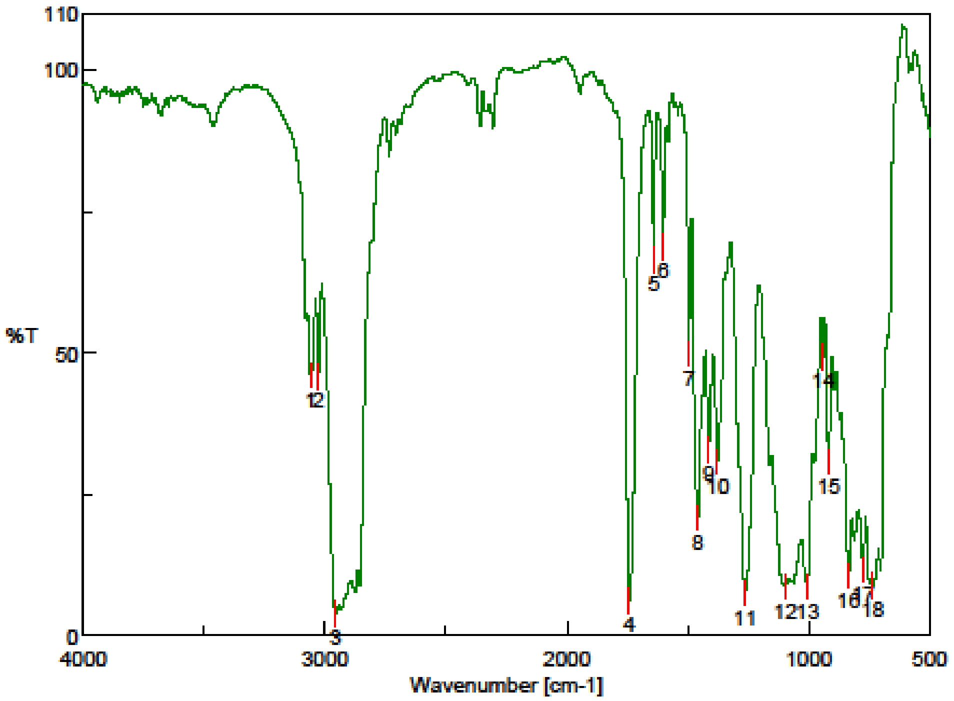

[ピーク検出結果]

No.	位置	強度	No.	位置	強度
1	3060.48	45.9578	2	3027.69	45.6808
3	2956.34	3.75561	4	1742.37	6.12336
5	1641.13	66.4076	6	1602.56	68.5953
7	1495.53	49.7077	8	1457.92	20.7747
9	1414.53	32.7735	10	1376.93	30.7499
11	1264.11	7.42121	12	1097.3	8.66136
13	1005.7	8.63374	14	940.128	49.2395
15	917.95	30.6132	16	835.99	10.4936
17	775.244	11.6453	18	736.674	8.71372

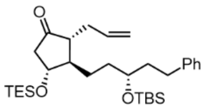

Comparison of ^{1}H NMR of vinyl addition

entry 3

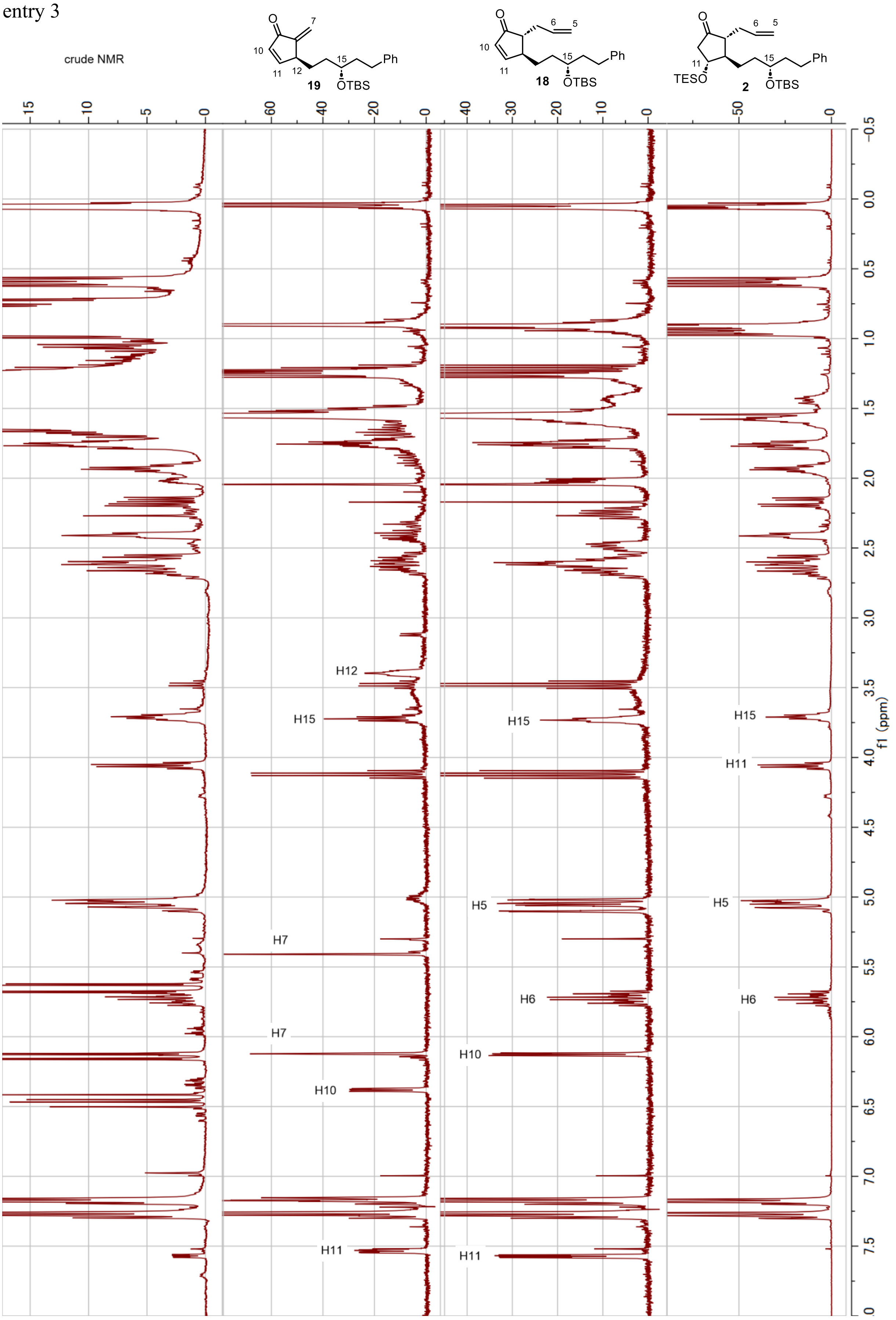

entry 4

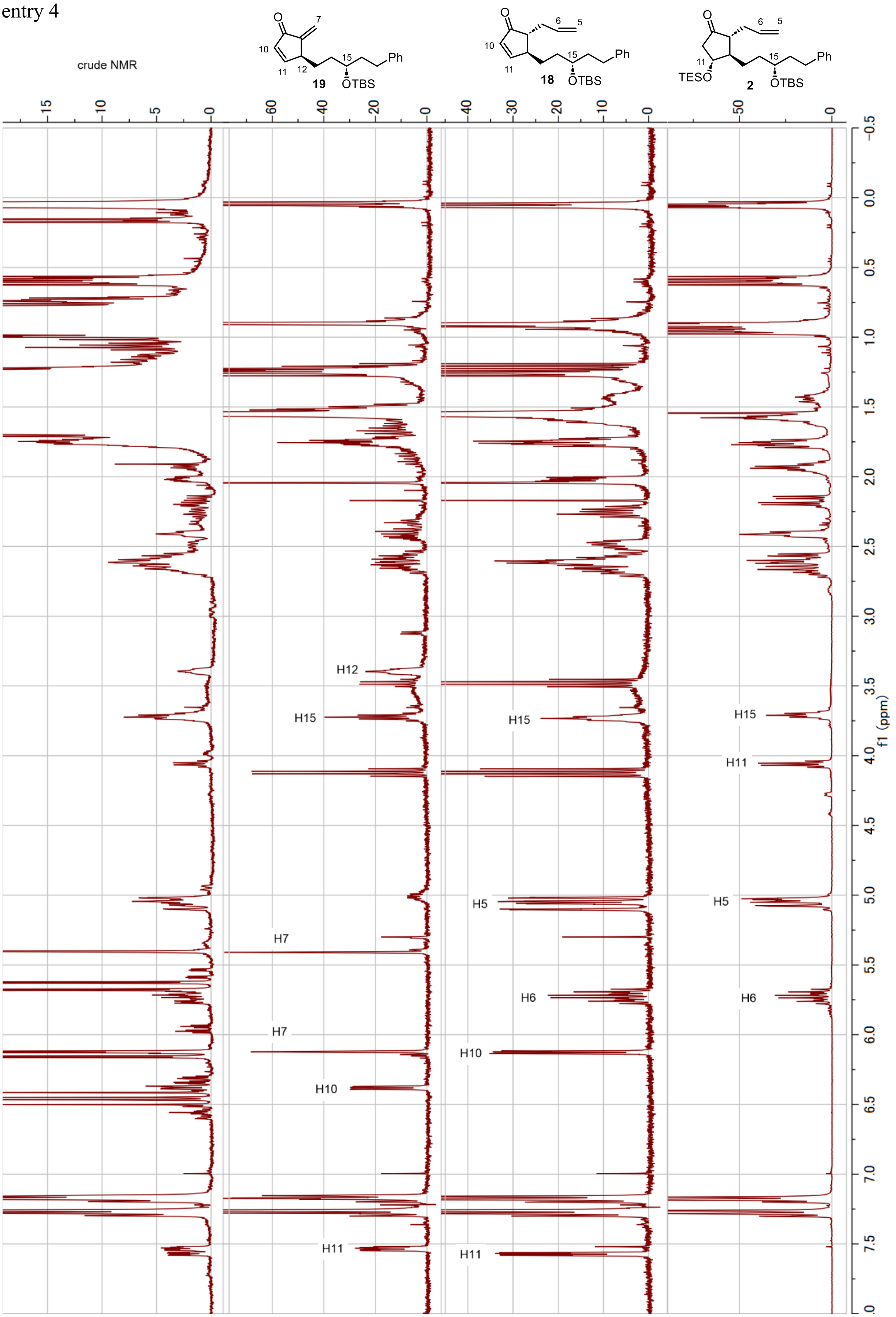

entry 7

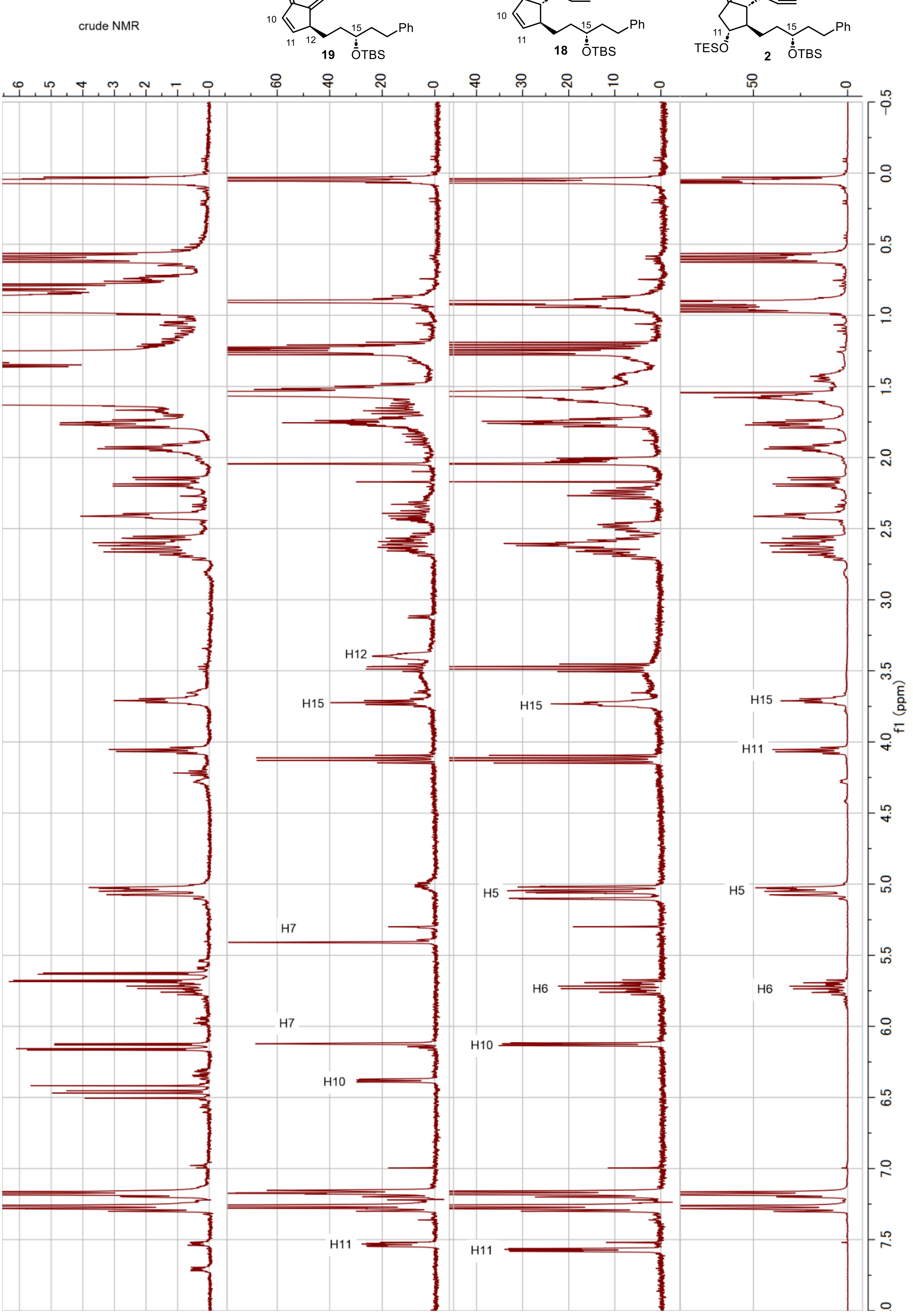

crude NMR
19
OTBS
Ph
18
OTBS
Ph
TESO
2
OTBS
Ph
H12
H15
H7
H7
H10
H11
H5
H15
H6
H10
H11
H15
H11
H5
H6
f1 (ppm)

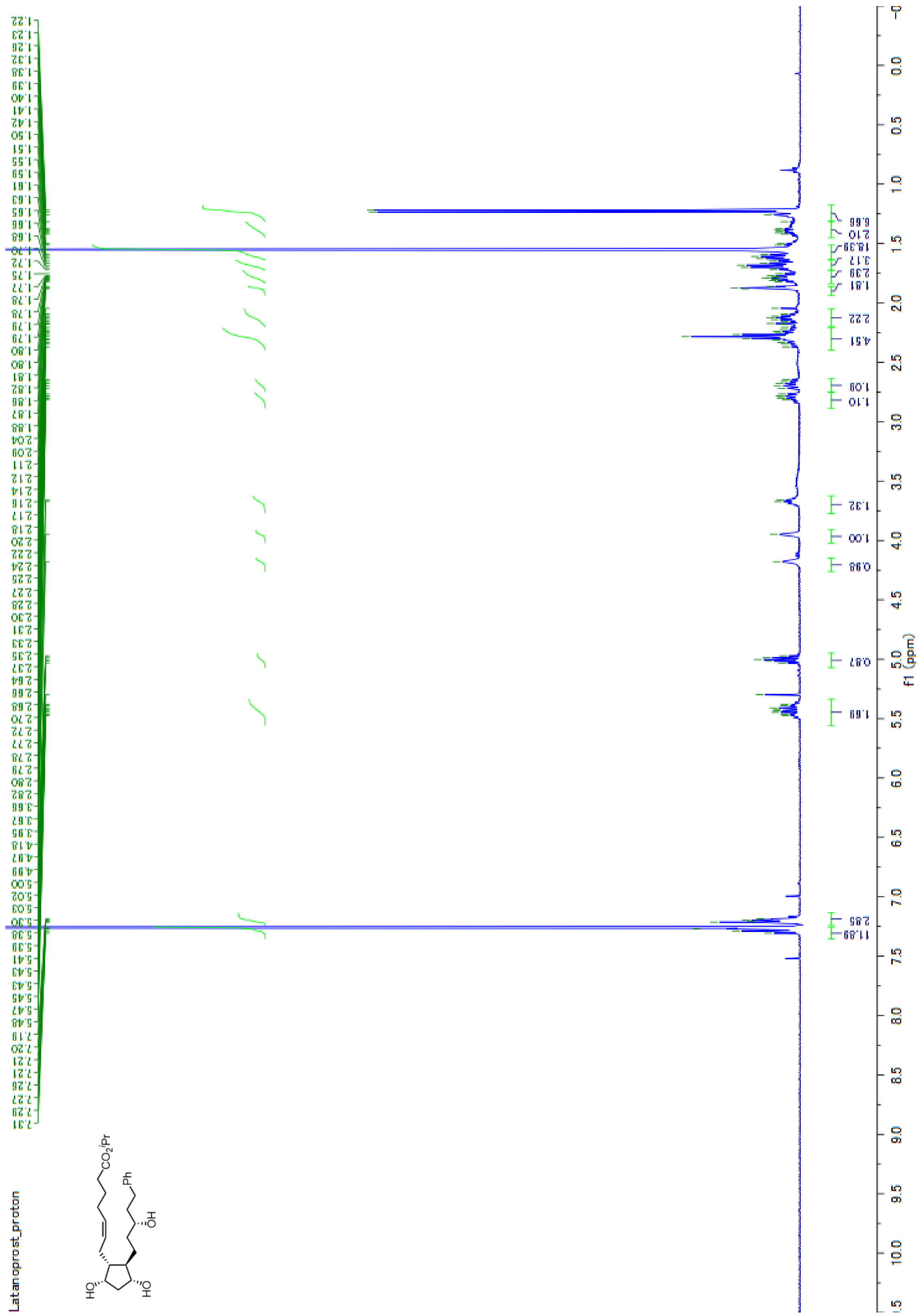
Latanoprost_proton
HO
HO
OH
Ph
CO2iPr
f1 (ppm)

Coparison of ^{1}H NMR of latanoprost

Reported[4]

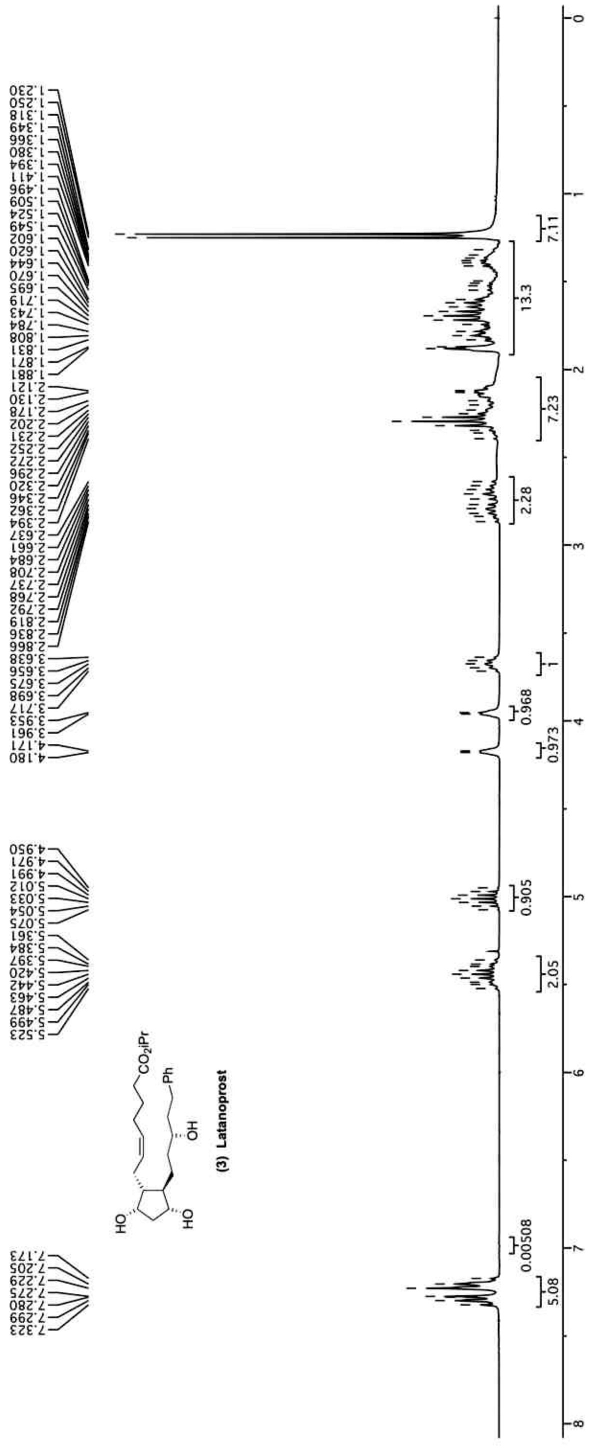

Synthesized

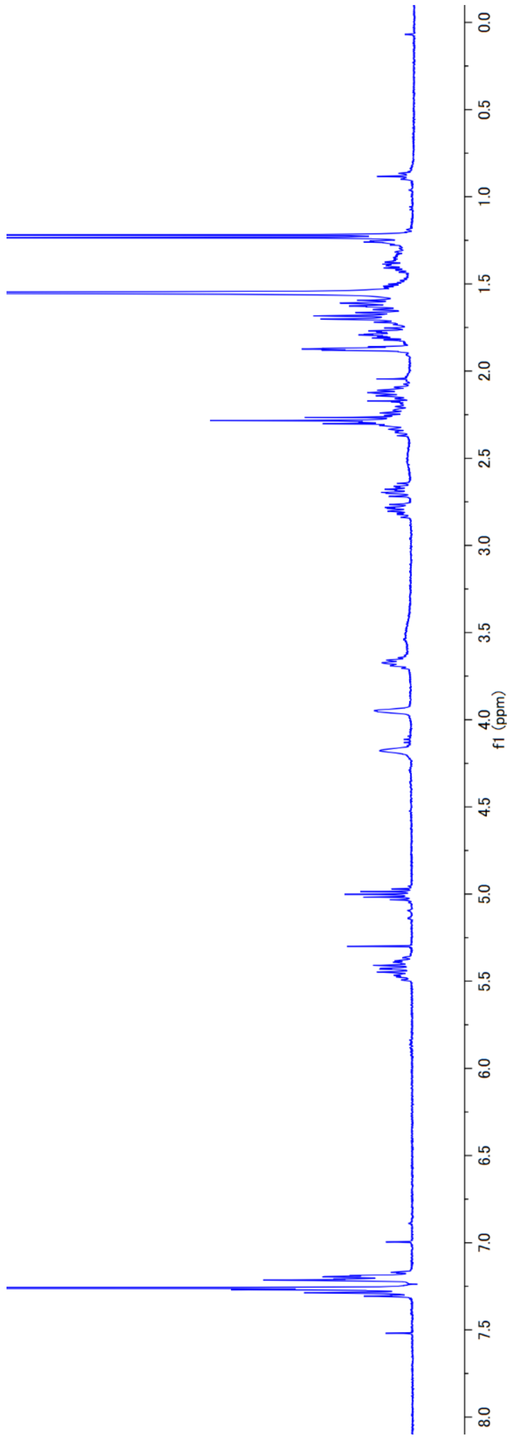

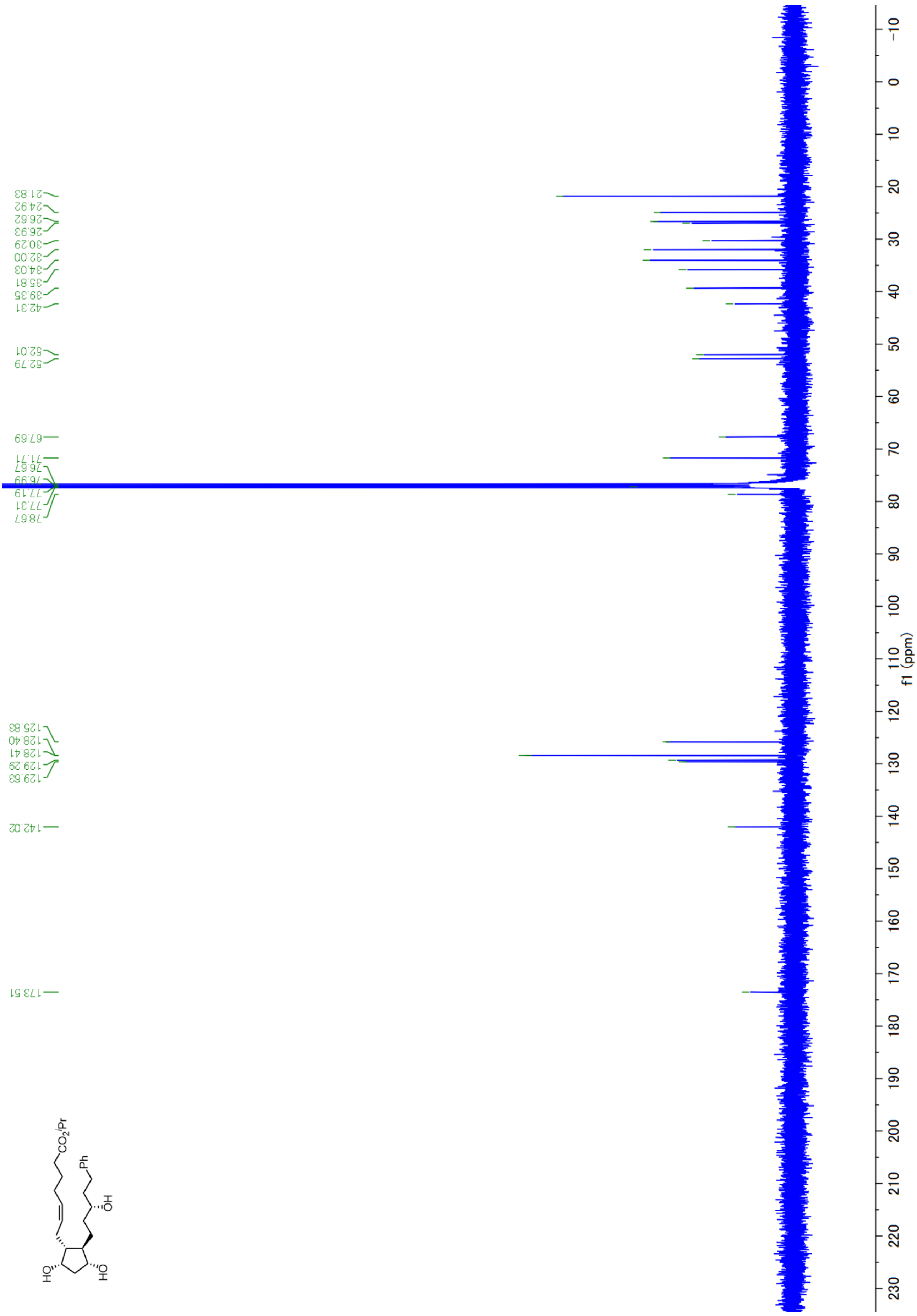
21.83
24.92
26.62
26.93
30.29
32.00
34.03
35.81
39.35
42.31
52.01
52.79
67.69
71.71
76.67
76.99
77.19
77.31
78.67
125.83
128.40
128.41
129.29
129.63
142.02
173.51
HO
HO
OH
$CO_2{}^iPr$
Ph
f1 (ppm)
